# Neurogenomic Profiling Reveals Distinct Gene Expression Profiles Between Brain Parts That Are Consistent in *Ophthalmotilapia* Cichlids

**DOI:** 10.3389/fnins.2018.00136

**Published:** 2018-03-09

**Authors:** Sofie Derycke, Loic Kéver, Koen Herten, Koen Van den Berge, Maarten Van Steenberge, Jeroen Van Houdt, Lieven Clement, Pascal Poncin, Eric Parmentier, Erik Verheyen

**Affiliations:** ^1^Operational Direction Taxonomy and Phylogeny, Royal Belgian Institute for Natural Sciences, Brussels, Belgium; ^2^Department of Biology, Ghent University, Ghent, Belgium; ^3^Laboratory of Functional and Evolutionary Morphology, University of Liège, Liège, Belgium; ^4^Behavioural Biology Unit, Ethology and Animal Psychology, University of Liège, Liège, Belgium; ^5^Department of Human Genetics, Genomics Core Facility, KU Leuven, Leuven, Belgium; ^6^Department of Applied Mathematics, Computer Science and Statistics, Ghent University, Ghent, Belgium; ^7^Bioinformatics Institute Ghent, Ghent University, Ghent, Belgium; ^8^Section Vertebrates, Ichthyology, Royal Museum for Central Africa, Tervuren, Belgium

**Keywords:** cerebellum, social behavior, cichlid fish, equivalence testing, gene-level FDR, neurogenomics

## Abstract

The detection of external and internal cues alters gene expression in the brain which in turn may affect neural networks that underly behavioral responses. Previous studies have shown that gene expression profiles differ between major brain regions within individuals and between species with different morphologies, cognitive abilities and/or behaviors. A detailed description of gene expression in all macroanatomical brain regions and in species with similar morphologies and behaviors is however lacking. Here, we dissected the brain of two cichlid species into six macroanatomical regions. *Ophthalmotilapia nasuta* and *O. ventralis* have similar morphology and behavior and occasionally hybridize in the wild. We use 3′ mRNA sequencing and a stage-wise statistical testing procedure to identify differential gene expression between females that were kept in a social setting with other females. Our results show that gene expression differs substantially between all six brain parts within species: out of 11,577 assessed genes, 8,748 are differentially expressed (DE) in at least one brain part compared to the average expression of the other brain parts. At most 16% of these DE genes have |log_2_FC| significantly higher than two. Functional differences between brain parts were consistent between species. The majority (61–79%) of genes that are DE in a particular brain part were shared between both species. Only 32 genes show significant differences in fold change across brain parts between species. These genes are mainly linked to transport, transmembrane transport, transcription (and its regulation) and signal transduction. Moreover, statistical equivalence testing reveals that within each comparison, on average 89% of the genes show an equivalent fold change between both species. The pronounced differences in gene expression between brain parts and the conserved patterns between closely related species with similar morphologies and behavior suggest that unraveling the interactions between genes and behavior will benefit from neurogenomic profiling of distinct brain regions.

## Introduction

Behavioral responses to external and internal cues are essential for organismal survival and reproductive success, because they allow organisms to find food, flee from predators and find mating partners, amongst others. The detection of external cues is mediated by sensory structures (e.g., eye, ear, olfactory membranes, taste, and pain receptors) which transduce the information to the central nervous system via neurons and synaptic junctions. There is a tight interaction between electrical activity, hormones and gene expression in neural networks underlying behavior (Newman, 1999; Robinson et al., 2008; Oliveira, 2012) and physiological responses (O'Connell and Hofmann, 2011; Albert et al., 2012; Heyne et al., 2014; Di Poi et al., 2016).

The vertebrate brain is divided in morphologically distinct but interconnected structures that are well conserved across different taxa (Northcutt, 2002). These brain parts are functionally distinct: the nuclei of two behavioral neural networks (the social behavior network and mesolimbic reward system) are mainly located in the diencephalon and in the telencephalon, respectively (O'Connell and Hofmann, 2011; Bshary et al., 2014). Neurons and receptors mediating the production of hormones related to reproduction are located in the forebrain (diencephalon, telencephalon, and olfactory bulbs) (Zohar et al., 2010; Gopurappilly et al., 2013; Prasad et al., 2015). The telecephalon is mostly involved in learning, memory and social behavior and the diencephalon regulates hormone production and signaling. The cerebellum has an important role in implementing motory programs (Roberts et al., 1992) and spatial and emotional learning (Yoshida et al., 2004). These functional differences are reflected in brain region specific gene expression profiles in different model organisms (Khaitovich et al., 2004; Lein et al., 2007; Myers et al., 2015).

At the molecular level, temporal and spatial variation in gene expression change the structure of the neural network by rewiring or biochemically switching nodes of the neural network (Cardoso et al., 2015). Different neurogenomic states correspond to different behavioral states and the switches between states are mediated by signaling pathways that interface the environment and the genotype (Oliveira, 2012). Immediate early genes (IEG) represent the earliest genomic response to environmental cues and have proven valuable as candidate genes for understanding behavioral responses (Burmeister et al., 2005; Wood et al., 2011; Cummings, 2015). Yet, many behaviors are generated through a network of genes with most of them only showing minor changes in gene expression (Sih et al., 2004). Moreover, reponses to social stimuli can be massive, involving thousands of genes potentially in many brain regions at once (Robinson et al., 2008). As such, behavioral responses are probably better linked to changes in brain neurogenomic states than to candidate genes. Comparison of differential gene expression in the brain within and between individuals with different behaviors has pointed to the genetic basis of tameness/agressiveness in rats (Heyne et al., 2014). Comparisons between species have shown that genes underlying domestication of dogs, pigs and rabbits are species-specific (Albert et al., 2012) and that behavioral differences between humans and other primates are probably linked to species-specific gene expression in the brain (Enard et al., 2002). In contrast, neurogenomic profiles between sister species that are similar in morphology and behavior are, to our knowledge, not yet available.

The same neural circuits, or the same genes within neural circuits, can be involved in conflicting behaviors (Sih et al., 2004), sometimes with opposing gene expression levels (Cummings et al., 2008; Wong and Hofmann, 2010; Sanogo et al., 2012; Sanogo and Bell, 2016). Moreover, gene expression can be cell type- and brain region specific (Sanogo et al., 2012) and extensive variation in gene expression between tissues, individuals and populations occurs (Whitehead and Crawford, 2005). Adequately linking gene expression in the brain to behavior therefore requires accurate profiling of the expression of all genes in each of the brain regions and in several individuals. At the same time, statistical models need to account for correlation between the different brain regions from the same individual. In addition, detecting differential expression between and across treatments requires methods that allow gene-level FDR control as often complex experimental designs typically involve many research hypotheses that have to be assessed for every individual gene (Van den Berge et al., 2017).

Here we assess differential expression in six morphologically defined brain regions of two congeneric cichlid fish species from Lake Tanganyika using 3′ mRNA sequencing (Moll et al., 2014) and a stage-wise statistical data analysis procedure (Hanssens et al., 1999; Nevado et al., 2011; Van den Berge et al., 2017). We study differential gene expression in the brain of mature female *Ophthalmotilapia nasuta* and *O. ventralis* that have been kept in a social setting with conspecific females under controlled laboratory conditions. The two species have similar morphology (Hanssens et al., 1999), similar behavior (Kéver et al., 2017) and occasionally hybridize in the field (Nevado et al., 2011). They are maternal mouthbrooders and females take care of the fry (Konings, 2014). Mating behavior is therefore assumed to be largely controlled by the female. We hypothesize that (1) gene expression profiles between brain parts within each species will be highly different because neural circuits, receptors for neurotransmitters and hormone production are located in specific brain regions and that (2) gene expression profiles across brain parts between species will reflect their similar morphology, behavior, and cognitive abilities. We also use the 3′ mRNAseq data to investigate differential expression between brain parts of 36 genes that have been linked in previous studies to fish behavior and physiology.

## Materials and methods

### Specimen collection

Wild-caught female fish of *O. ventralis* (OV) and *O. nasuta* (ON) were bought from Cichlidenstadl (Allerheim, Germany) and arrived at the lab on February 24, 2015. The individuals of *O. ventralis* and *O. nasuta* were collected at Ulwile Island and Mtosi, respectively.

### Experimental setup

Five female fish from each species were kept in aquaria containing aerated freshwater of 28°C, and received a light-dark cycle of 12:12 h for 51 days after arrival in the lab. The *O. nasuta* females were kept in an aquarium with a water volume of 270 L (W40 × H50 × L135 cm), while *O. ventralis* females were kept in a slightly smaller aquarium containing a water volume of 216 L (W50 × H39 × L111 cm). The two aquaria were located in the same room, and recieved the same maintenance throughout the 51 day period. All experimental procedures were approved by the University of Liège Institutional Animal Care and Use Committee (protocol number 1759).

### RNA extraction

Five females per species were caught, tapped on the neurocranium to knock them out and killed by cutting the spine just after the neurocranium. All ten females were killed on the same day (April 16th, 2015) between 11.30 h and 16 h. The brain atlas from *Oreochromis mossambicus* (Simões et al., 2012) was used to locate the main brain regions. For each specimen, the cerebellum (CE) was dissected first, after which the telencephalon (TE) + olfactory bulbs (OB) were removed and then separated on a glass slide and stored in RNA later. Subsequently, the two lobes of the optic tectum (OT), the brain stem (BS) and the diencephalon (DI, including the preoptic area, hypothalamus and pituitary) were removed and stored in RNA *later* Stabilization Solution (Invitrogen). Time between catching the fish and dissection of the last brain part varied between 14 and 21 min. The five specimens of *O. nasuta* were dissected first, after which the five specimens of *O. ventralis* were dissected. The parts (6 parts × 10 specimens = 60 samples in total) were stored in RNA later at room temperature for one night, after which they were transferred to −80°C until further processing. After dissection of the brain, the specimen was dissected further to verify the presence of ovaria with eggs to ensure all females were mature.

RNA extraction was performed using the RNeasy Lipid Tissue Mini Kit from Qiagen following the manufacturer's protocol. Brain tissues were homogenized using pestles and a cordless motor (Sigma Aldrich). RNA extractions were performed in batches of 12 samples that were randomly taken from the total collection of 60 samples. Hence, samples from different specimens and different brain parts were extracted per batch. Quality and quantity of the RNA samples were checked using the Bioanalyzer (Agilent Technologies). Out of the 60 samples, 50 samples had RIN values above nine and six samples had RIN values between 7.6 and 8.9. All 56 samples showed two clear peaks corresponding to the 18S and 28S ribosomal RNA, indicating a low level of degradation. Four OB samples had too low amount of RNA (<10 ng/μl).

### RNA library preparation and sequencing

Library preparation for next generation sequencing was performed by the Genomics Core facility of KULeuven. The 60 RNA samples were prepared using the QuantSeq 3′ mRNA-Seq Library Prep Kit for Illumina (Lexogen). The method has high strand specificity (>99.9%) and most sequences are generated from the last exon and the 3′ untranslated region. The method generates only one fragment per transcript and the number of reads mapped to a given gene is proportional to its expression. Fewer reads than classical RNAseq are needed to determine unambiguous gene expression levels, allowing a high level of multiplexing (Moll et al., 2014). Library preparation involved reverse transcription of RNA with oligodT primers, followed by removal of RNA and second strand cDNA synthesis with random primers. The resulting fragments containing both linker fragments were PCR amplified with primers that also contain the Illumina adaptors and sample specific barcodes. All 60 libraries were pooled and sequenced (single-end 50 bp) on one lane of the Illumina Hiseq 2500.

### Data analysis

#### Read trimming and mapping

Using Trimmomatic 0.36 (Bolger et al., 2014), we trimmed the first 10 bp to remove possible introduced errors due to the second strand synthesis based on random priming and removed poly A tails. Only trimmed reads with a length >20 bp were retained. The trimmed reads were mapped to the *Oreochromis niloticus* genome version ASM185804v2 using default settings in STAR 2.5.2b (Dobin et al., 2013). Mapped reads were then processed with SAMtools 1.1 (Li et al., 2009; Li, 2011) and Picard tools 2.2.2 (cite site: https://broadinstitute.github.io/picard/), resulting in a position-sorted bam file containing all read metadata information. Read counting was done using HTSeq-count 0.6.1p1 (Anders et al., 2015) using gene features defined in ensemble annotation version 103. Counting was done using a strict strategy, which uses the strand information (reads had to be on the same strand as the feature). The mapping quality was set to 10. Reads were discarded when they overlapped two features.

#### Statistical analysis

One sample (Co1Na_OB_5) contained a substantially lower number of reads (62,681) compared to all other samples (min 325 869; 57 out of the 60 samples had > 500,000 reads) and was removed. Genes with low overall counts (threshold of at least 15 cpm in at least four samples) were removed from the analysis. We corrected for differences in sequencing depth and RNA population using a weighted trimmed mean of the log expression ratios (TMM) normalization (Robinson and Oshlack, 2010). A multidimensional scaling (MDS) plot of Euclidean distances based on the gene expression profiles of the top 500 genes was created using the limma package (Ritchie et al., 2015).

We fitted gene-wise negative binomial generalized linear models (GLMs) implemented in edgeR v3.12.1 (Robinson et al., 2010; Lund et al., 2012) with fixed effects for individual, species (*O. nasuta* and *O. ventralis*), brain parts (BS, OB, OT, CE, TE, DI) and species x brain part interactions. The individual effects are necessary to account for the clustered design, i.e., different brain parts are sampled within each individual. The interaction effect is required to accomodate for species-specific expression in different brain regions. The quasi-likelihood framework was used for parameter estimation and statistical inference (Lund et al., 2012). We assessed two sets of hypotheses: (1) gene expression does not differ between brain parts within each species and (2) gene expression differences between brain parts do not differ between species (involving differences between species × brain part interaction terms). Hypothesis tests were assessed using a stage-wise testing procedure implemented in the R package stageR to allow for gene-level false discovery rate (FDR) control when assessing multiple research hypotheses for each gene (Van den Berge et al., 2017). In the screening stage, the global null hypothesis (i.e., testing whether any of the null hypotheses of interest are false) was tested on a 5% FDR level using the Benjamini-Hochberg method (Benjamini and Hochberg, 1995). In the second stage, all hypotheses were tested separately only for the significant genes from the screening stage and the within-gene family-wise error rate (FWER) was controlled on the adjusted FDR level from the screening stage using Holm's method (Holm, 1979). The procedure guarantees to control the gene-level FDR at 5% (Heller et al., 2009). In addition, using the DE genes from the screening stage we tested for |log_2_FC| significantly larger than 2 using a test for differential expression relative to a threshold (McCarthy and Smyth, 2009) on a 5% gene-level FDR. For the first set of hypotheses, differential expression between brain parts was tested by comparing the gene expression in a particular brain part with the average gene expression across the remaining five brain parts within each species. We defined 12 contrasts: BS-avg_Na, CE-avg_Na, DI-avg_Na, OB-avg_Na, OT-avg_Na, TE-avg_Na, BS-avg_Ve, CE-avg_Ve, DI-avg_Ve, OB-avg_Ve, OT-avg_Ve, TE-avg_Ve. For the second set of hypotheses, differential expression between brain parts across species was investigated by defining 15 contrasts (BSvCE_Na-BSvCE_Ve, BSvDI_Na-BSvDI_Ve, BSvOB_Na-BSvOB_Ve, BSvOT_Na-BSvOT_Ve, BSvTE_Na-BSvTE_Ve, CEvDI_Na-CEvDI_Ve, CEvOB_Na-CEvOB_Ve, CEvOT_Na-CEvOT_Ve, CEvTE_Na-CEvTE_Ve, DIvOB_Na-DIvOB_Ve, DIvOT_Na-DIvOT_Ve, DIvTE_Na-DIvTE_Ve, OBvOT_Na-OBvOT_Ve, OBvTE_Na-OBvTE_Ve, OTvTE_Na-OTvTE_Ve). We did not consider differential expression between species within the same brain part because statistical tests implemented in state-of-the-art RNA-seq data analysis tools cannot correct for variability within and between individuals, moreover, main effects between species are also confounded with the aquarium effect. The 200 genes with lowest screening test adjusted *p*-values were used to generate a heatmap in the R library gplots (Warnes et al., 2016).

Finally, we performed an equivalence test to investigate whether differences in gene expression profiles between both species are conserved. We consider an equivalence interval of log_2_FC from −2 to 2. The design matrix is reparametrized such that one contrast corresponds to one coefficient and perform two one-side tests (TOST) by adjusting the offset for the coefficient of interest by −2 and 2. The equivalence test *p*-value then corresponds to the maximum *p*-value from the two one-sided tests (Schuirmann, 1987). We test for equivalence within each contrast by controlling the FDR at each contrast at a 5% level. Moreover, we also test for genes that are equivalent across all 15 contrasts by controlling the FDR on the within-gene maximum *p*-value across all 15 contrasts.

Gene ontology (GO) terms of all DE genes after the confirmation step were obtained using the *Oreochromis niloticus* dataset in BiomaRt (Durinck et al., 2005). GO terms related to “Biological Processes” were selected for further analysis. Enriched GO terms for each brain part were determined using a competitive gene set test implemented in CAMERA (Wu and Smyth, 2012). This test determines whether genes in the set are highly ranked in terms of differential expression (DE) relative to genes not in the set. The enrichment analysis was performed using genes that were DE in only one brain part in both species (these genes are likely to contribute most to the functional differences between brain parts) relative to all DE genes in the dataset. Two-tailed *p*-values were corrected on a 5% FDR level using the Benjamini-Hochberg method (Benjamini and Hochberg, 1995). GO terms that are significant in both species were visualized with the GOBubble and GOHeat function of R package GOplot (Wencke et al., 2015).

#### Expression of genes linked to fish behavior and physiology

We screened the 3′ mRNA sequencing data for the presence of five immediate early genes, 15 behavioral genes and 16 receptor genes that have been linked to fish behavior and physiological networks in previous studies (full list of genes and the studies are presented in Table S4). This allows to investigate their expression in the six brain regions. The log_2_ fold changes and adjusted *p*-values from the statistical test of hypothesis 1 were used to assess differential expression between brain parts.

## Results

### Data exploration

The number of trimmed reads per sample ranged between 62,681 and 3,249,822. On average, 53.4% of the reads uniquely mapped against the *O. niloticus* genome and only a small fraction (2.5–5.3%) of the reads were mapped to multiple loci (Figure S1).

After filtering and normalization, 11,577 genes were kept in the dataset, the bulk of which were expressed in all 59 samples (7,224 genes, 62.4%). The total number of genes expressed in each sample was very similar and ranged between 9,565 and 11,499 (median 11,324). Not a single gene was uniquely expressed in one brain part.

**Figure 1:**
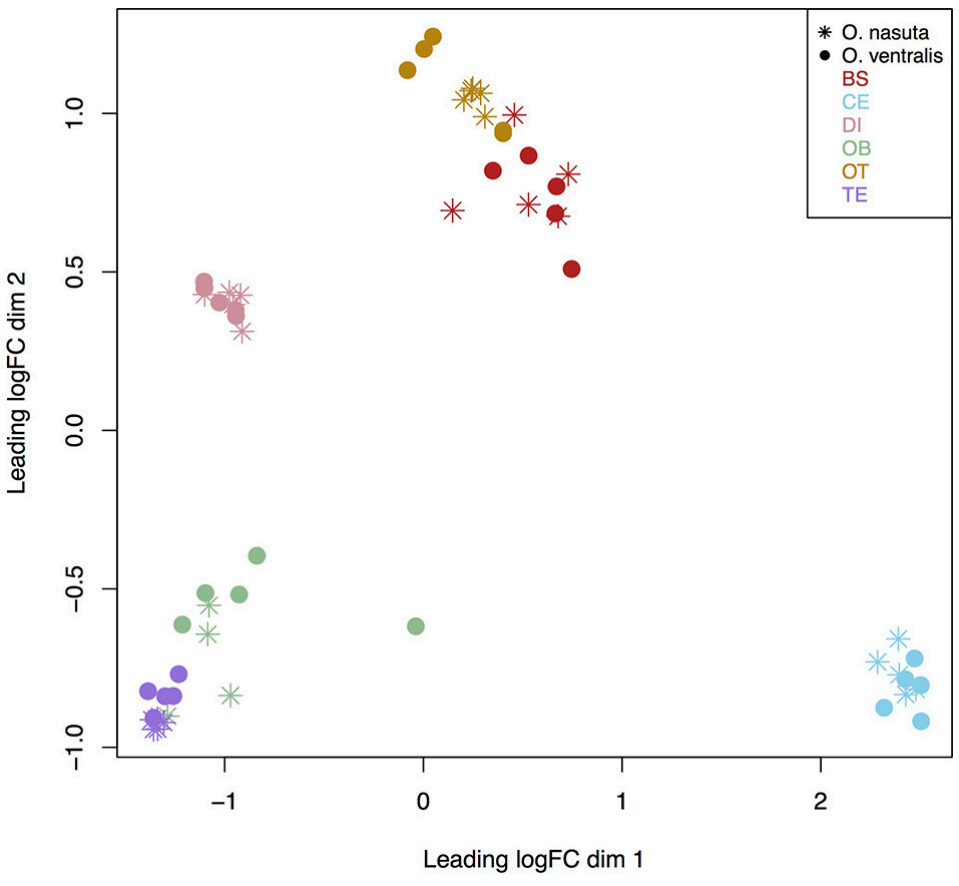
MDS plot of all 59 samples used for analysis. Distances between samples reflect log_2_fold changes between the 500 most variable genes. The MDS plot clearly shows a separation based on brain part for both species. BS, brain stem; CE, cerebellum; DI, diencephalon; OB, olfactory bulb; OT, optic tectum; TE, telencephalon.

The MDS plot revealed a clear clustering based on brain part for both species. The cerebellum was the most distinct brain part (Figure 1). The diencephalon was also separated from all other brain parts, while the telencephalon and olfactory bulbs on the one hand, and the optic tectum and brain stem on the other hand, were more similar in gene expression to each other than to the other brain parts.

### Differential gene expression between brain parts within species

**Figure 2:**
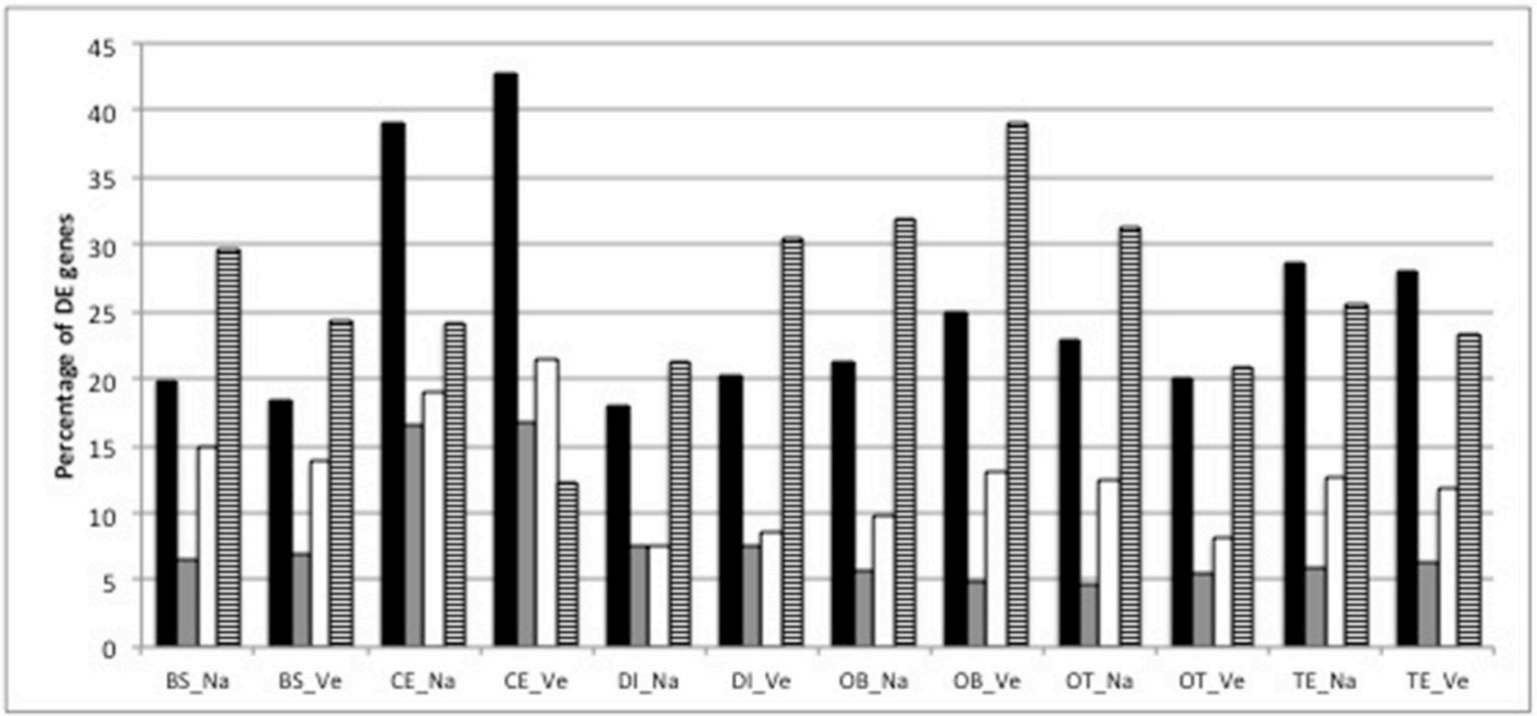
Percentage of DE genes (black bars), percentage of DE genes with |log_2_ FC| >2 (gray bars), percentage of DE genes that are unique for each brain part within each species (white bars) and percentage of DE genes that are unique for each species and brain part (striped bar) (BS, brainstem; CE, cerebellum; DI, diencephalon; OB, olfactory bulb; OT, optic tectum; TE, telencephalon; Na: *O. nasuta*; Ve: *O. ventralis*). The percentage of DE genes was calculated as the number of DE genes divided by the average number of expressed genes across the biological replicates (four for OB_Na, five for the other brain parts) for each brain part.

The two-stage testing procedure yielded 8748 genes that show differential expression between at least one brain part and the average of the remaining brain parts on a 5% gene-level FDR. These DE patterns are consistent between species (Figure 2): the cerebellum contains the highest number of DE genes (average 4,455 DE genes), followed by the telencephalon (average 3,193). The majority (61–88%) of genes that were DE in a particular brain part were shared between both species (Figure 2). The heatmap with gene expression values of the 200 most significant genes clusters the samples from both species according to brain part and shows a pronounced difference in gene expression in the cerebellum compared to all other brain parts (Figure S2). The heatmap also illustrates the high similarity in gene expression between telencephalon and olfactory bulbs (Figure S2).

The number of DE genes with |log_2_ FC| above 2 (i.e., at least a fourfold increase or decrease in gene expression) was below 8% in most brain parts for both species, except for the cerebellum, where 16% of the DE genes had a fourfold higher expression compared to the average expression in the other brain parts (Figure 2). The cerebellum also contained the highest proportion of DE genes that were only significant for the cerebellum (19 and 21% for *O. nasuta* and *O. ventralis*, respectively; Figure 2). DE genes with |log_2_ FC| > 2 are always upregulated in the brainstem compared to the average expression in the other brain parts (Figure S2, Table S1). A detailed list of the DE genes that are substantially up- or downregulated (i.e., |log_2_ FC| significantly > 2) in a particular brain part for both species along with their GO annotation is presented in Table S1.

**Figure 3:**
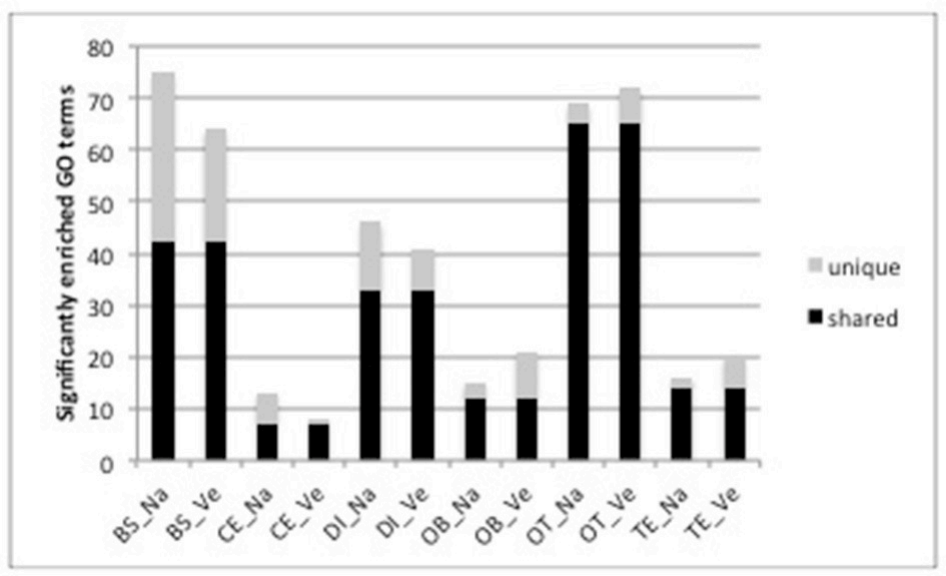
Number of significantly enriched GO terms for each brain part and each species determined by CAMERA. The height of the bar shows the total number significantly enriched GO terms. (BS, brainstem; CE, cerebellum; DI, diencephalon; OB, olfactory bulb; OT, optic tectum; TE, telencephalon; Na: *O. nasuta*; Ve: *O. ventralis*). The number of significantly enriched GO terms that are shared between the two species for each brain part are indicated in black. A large proportion (54–94%) of the significantly enriched GO terms was shared between both species.

For functional analysis, we focus on those genes that were uniquely DE in one brain part across both species since these are the genes most likely to contribute to the functional differences between brain parts. In total, genes that were uniquely DE in one brain part yielded 1,720 and 1,734 GO terms for *O. nasuta* and *O. ventralis* respectively. The brain stem and optic tectum contained a high number of significantly enriched GO terms (BS: 75 and 64 for *O. nasuta* and *O. ventralis*, respectively; OT: 69 and 72 for *O. nasuta* and *O. ventralis*, respectively, Figure 3). A large proportion (54–94%) of the significantly enriched GO terms were shared between both species (Figure 3). The direction of regulation, number of genes involved and the significance level of the enriched GO terms were highly similar between the two species, but differed substantially between brain parts (Figure S3). A small number of GO terms that were significantly enriched in both species were enriched in more than one brain part (BS: 31, CE: 4, DI: 26, OB: 3, OT: 49, TE: 14).

We subsequently looked at the top 5 upregulated biological processes that were significantly enriched in both species (Table 1). The brain stem and optic tectum contained biological processes that were mediated by a large number of genes (Table 1). For the brain stem, these were multicellular organism development and lipid metabolic process. For the optic tectum, these were cell adhesion, homophilic cell adhesion via plasma membrane adhesion molecules and ionotropic glutamate receptor signaling pathway. For the diencephalon, the ephrin receptor signaling pathway contained 17 DE genes, while the other enriched processes contained much less DE genes. The cerebellum, olfactory bulbs and telencephalon contained only enriched processes with a low number of DE genes (Table 1). The full list of biological processes enriched in each brain part for both species can be found in Table S2.

**Table 1:** Top 5 upregulated GO terms that were identified by CAMERA as enriched in both species.

***O. nasuta***					***O. ventralis***		
	**GO term**	**Biological Process**	**# DE genes**		**GO term**	**Biological process**	**# DE genes**
BS	GO:0007275	Multicellular organism development	62		GO:0007275	Multicellular organism development	63
	GO:0030902	Hindbrain development	12		GO:0006629	Lipid metabolic process	26
	GO:0006629	Lipid metabolic process	27		GO:0030902	Hindbrain development	12
	GO:0031018	Endocrine pancreas development	9		GO:0060215	Primitive hemopoiesis	11
	GO:0060215	Primitive hemopoiesis	9		GO: 0006935	Chemotaxis	7
OT	GO: 0007155	Cell adhesion	82		GO: 0007155	Cell adhesion	86
	GO:0007156	Homophilic cell adhesion via plasma membrane adhesion molecules	44		GO:0007156	Homophilic cell adhesion via plasma membrane adhesion molecules	42
	GO:0035235	Ionotropic glutamate receptor signaling pathway	23		GO:0035235	Ionotropic glutamate receptor signaling pathway	24
	GO:0008543	Fibroblast growth factor receptor signaling pathway	8		GO:0043049	Otic placode formation	6
	GO:0030902	Hindbrain development	12		GO:0030902	Hindbrain development	12
DI	GO:0048013	Ephrin receptor signaling pathway	17		GO:0048013	Ephrin receptor signaling pathway	17
	GO:0006182	cGMP biosynthetic process	5		GO:0009607	Response to biotic stimulus	5
	GO:0009607	Response to biotic stimulus	5		GO:0044319	Wound healing, spreading of cells	3
	GO:0044319	Wound healing, spreading of cells	3		GO:1902766	Skeletal muscle satellite cell migration	3
	GO:0060729	Intestinal epithelial structure maintenance	3		GO:0007205	Protein kinase C-activating G-protein coupled receptor signaling pathway	9
CE	GO:0001841	Neural tube formation	4		GO:0001841	Neural tube formation	4
	GO:0009880	Embryonic pattern specification	3		GO:0009880	Embryonic pattern specification	3
	GO:0007586	Digestion	1		GO:0007586	Digestion	1
	GO:0048739	Cardiac muscle fiber development	1		GO:0048739	Cardiac muscle fiber development	1
TE	GO:0038170	Somatostatin signaling pathway	4		GO:0038170	Somatostatin signaling pathway	4
	GO:0048268	Clathrin coat assembly	3		GO:0048915	Posterior lateral line system development	3
	GO:0048915	Posterior lateral line system development	3		GO:0001706	Endoderm formation	2
	GO:0001706	Endoderm formation	2		GO:0001714	Endodermal cell fate specification	2
	GO:0001714	Endodermal cell fate specification	2		GO:0038171	Cannabinoid signaling pathway	2
OB	GO:0038170	Somatostatin signaling pathway	4		GO:0038170	Somatostatin signaling pathway	4
	GO:0007195	Adenylate cyclase-inhibiting dopamine receptor signaling pathway	3		GO:0007195	Adenylate cyclase-inhibiting dopamine receptor signaling pathway	3
	GO:0048915	Posterior lateral line system development	3		GO:0051091	Positive regulation of sequence-specific DNA binding transcription factor activity	3
	GO:0051091	Positive regulation of sequence-specific DNA binding transcription factor activity	3		GO:0038171	Cannabinoid signaling pathway	2
	GO:0038171	Cannabinoid signaling pathway	2		GO:0043584	Nose development	2

*The cerebellum contained only four significantly upregulated GO terms. Upregulated is here determined from the z-score of the GOplot package, which subtracts the number of downregulated genes (log_2_ FC <0) from the number of upregulated genes (log_2_ FC >0) in each GO term*.

### Species specific regulation between brain parts

The stage-wise testing procedure identified 32 genes with a differential FC between two brain parts between the species (i.e., the difference in brain part x species interactions). At most seven genes were significant in the respective contrasts. Of the 32 genes, 14 genes were not annotated, and the remaining genes are involved in 18 biological processes that are mainly linked to basic mechanisms such as transport, transmembrane transport, transcription (and its regulation) and signal transduction (Table S3). Gene LOC100695791 is involved in the steroid hormone mediated signaling pathway. The expression patterns of each of the 32 genes across the brain parts in both species are presented in Figure S4.

### Conservative regulation between brainparts

Above we have shown that there are minor differences in specific brain part effects between species. Here, we test whether the expression differences are indeed equivalent between species. We consider an equivalence interval of [−2, 2] for the difference in log fold change between two brain parts between the species. Assessing the same contrasts as above, we find that on average 89% of the genes show equivalent expression between species, with a range of 85% to 94% of equivalent fold changes across the contrasts on a 5% FDR level. Moreover, we find evidence for 71% of the genes to be equivalent across all between-species comparisons. These results provide strong evidence for conserved expression differences between the species.

### Gene expression of genes linked to fish behavior and physiology

Of the five IEG that have been linked to behavior in previous studies (Table S4), expression of two genes was detected in the brain of *Ophthalmotilapia* under our control conditions*: egr1* was expressed in all brain parts (Figure S5) and significantly more so in the telencephalon (Table S4) while *bdnf* was nearly absent in the cerebellum (at most five reads, Figure S5) and was significantly more expressed in the telencephalon and the olfactory bulbs in both species (Table S4). We did not detect *cfos*, but two other IEG from the *fos* family were expressed in all brain regions: *fosb* and *fosl2* (Figure S5). No significant differences between brain parts were found for these two genes.

Of the 16 behavioral and reproductive genes, the expression of nine genes was detected in the female brain (Figure S5): *gnrh1* and *gnrh3* were consistently expressed in the olfactory bulbs and telencephalon, and were significantly more expressed in the olfactory bulbs compared to the average expression in the other brain parts in both species (Table S4); *Vip* was expressed in the brainstem, diencephalon and the olfactory bulbs and was significantly more expressed in the brainstem and the diencephalon compared to the average expression in the other brain parts in both species (Table S4); *oxt* was consistently and significantly overexpressed in the brainstem (Table S4). The remaining five genes were expressed in all brain parts (Figure S5): *avpi1* was significantly more expressed in the cerebellum, *serpini1* in the diencephalon, telencephalon and olfactory bulbs and *gabarap* in the telencephalon compared to the average expression in the other brain parts in both species (Table S4). Expression of the *kpna1* and *nlgn3* genes were similar in all brain parts (Figure S5, Table S4).

Of the 17 receptor genes that we screened, only four were expressed in the brain of female *Ophthalmotilapia* under our control setting (Figure S5): the adrenergic receptor *adrb1* was highly expressed in the cerebellum (Figure S5, Table S4). The serotonergic receptor *htr1a* was significantly more expressed in the optic tectum and *htr2a* was significantly more expressed in the telencephalon, optic tectum and olfactory bulbs compared to the average gene expression in the remaining brain parts in both species (Table S4). The dopaminergic receptor *drd2* was significantly more expressed in the brainstem and the olfactory bulbs compared to the average gene expression in the remaining brain parts in both species (Table S4).

## Discussion

The Quantseq approach (Moll et al., 2014) and the two-stage statistical testing procedure (Van den Berge et al., 2017) allow to simultaneously investigate expression of more than 11,500 genes in a large number of samples (here: 60) and revealed more than 8,000 genes that were differentially expressed between brain parts while providing false positive control on the level of the gene. The Quantseq method generates a single sequence for each transcript of the last exon thereby eliminating the need to correct for exon number and transcript length differences between genes and greatly reduces sequencing depth per sample (Moll et al., 2014). Consequently, Quantseq is more cost-effective than classical RNAseq when only differential gene expression analysis is of interest; it does not allow to investigate differential splicing of exons or to build a de novo transcriptome. On average 53% of our data was uniquely mapped against the *O. niloticus* reference genome, and on average 84% of the uniquely mapped data was used for differential expression analyses.

### All six brain parts show pronounced differences in gene expression

The MDS plot showed a clear difference in gene expression between all six brain parts (Figure 1) and our statistical analysis showed that 75% of the genes are differentially expressed between brain parts. We argue that these differences are linked to functional differences of the brain parts rather than to their dissection order for the following reasons: (1) RNA quality of all samples, including the diencephalon which was dissected last, was high (RIN values ranged between 9.3 and 10 for DI samples) implying that mRNA degradation was limited. Even in metabolic highly active tissue such as liver, RNA quality did not decline at room temperature within the first 2 h (van Maldegem et al., 2008); (2) the prefixation time (i.e. the time between the death of the individual and placing the tissue in RNAlater) may significantly alter gene expression due to anoxia, pH changes and other biochemical changes in the tissues (Srinivasan et al., 2002). The prefixation time of our tissues was at most 5 min which is within the maximum prefixation time of 10 min suggested by Srinivasan et al. (2002); (3) none of the genes with |log_2_FC| > 2 had GO terms linked to catabolic processes, mRNA decay, mRNA stability, apoptosis or regulation of pH (acidification) (Table S1). In addition, the enriched GO terms for each brain part also did not point to mRNA decay (Table S2); (4) we did not observe a decrease of gene expression levels of the 200 most DE genes from the cerebellum, optic tectum, telencephalon, olfactory bulbs, brain stem to the diencephalon (Figure S2).

Similar to our results, pronounced differences in gene expression between four human brain regions (Myers et al., 2015), between forebrain, midbrain and hindbrain regions in female sticklebacks in a social context (Greenwood and Peichel, 2015) and in the telencephalon, diencephalon, cerebellum and brainstem of male sticklebacks in response to a territorial intrusion (Sanogo et al., 2012) have been observed. Our results demonstrate that also the olfactory bulbs and the optic tectum show clear differences in gene expression and suggest that subdividing the brain in macroanatomical regions is preferable over whole brain gene expression when a characterisation of gene networks underlying behavioral responses is of interest. Moreover, the macroanatomical division applied here may provide a framework to define microanatomical subdivisions and the component cell types for future more fine scale resolution of gene expression patterns related to behavior.

### Brain part specific functional annotation

The cerebellum is the most distinct brain part in terms of gene expression (Figure 1, Figure S2), contained the highest number of DE genes and 70% of the genes that showed a substantial DE (|log_2_ FC| > 2) in both species were uniquely DE in the cerebellum. Yet, the 1512 DE genes in the cerebellum in both species yielded only seven significantly enriched GO terms which were related to the development of cardiac muscle fibers, development of the lateral line and development of the neural tube, and to digestion and self-proteolysis (Table S2). This brain part is known to be involved in motory responses and emotions, and the development of cardiac muscle fibers and the lateral line can be linked to motory movement in fish. Our results also point to a role of the cerebellum in the physical, chemical, and biochemical processes to break down ingested nutrients into components that may be easily absorbed and directed into metabolism. Digestion of glycine, glutamine, glycerol and beta-hydroxybutyrate for energie production in the cerebellum has been documented in rats (Rotta et al., 2002).

The telencephalon is a region involved in learning and memory, and the molecular mechanism underlying these functions typically involve rewiring of the neuronal connections through changes in signaling pathways and cell communication, and the development of new and apoptosis of old cells (Cardoso et al., 2015). Five out of the 14 enriched GO terms in the telecephalon were linked to signaling pathways, and four were linked to cell formation (including clathrin coat assembly and endoderm formation). These four GO terms were uniquely enriched in the telencephalon.

The olfactory bulbs in *Ophthalmotilapia* are very small which complicated their dissection and yielded small quantities of RNA for sequencing. This technical issue may explain the larger variation across the replicates for this brain part (Figure 1). Nevertheless, the most upregulated enriched GO terms in the olfactory bulbs are involved in nose development, the cannabinoid signaling pathway and the positive regulation of feeding behavior which point to an important role of this brain part in detecting environmental cues related to obtaining food.

The brainstem, optic tectum and diencephalon contained a large number of enriched GO terms typically with a large number of DE genes (Table 1). The optic tectum plays an important role in sensory detection, and we found four GO terms related to placode formation, two GO terms related to neuromast deposition and two GO terms related to retina morphogenesis (Table S2). The optic tectum also contains many GO terms linked to signaling pathways, cell formation, communication and apoptosis suggesting that the machinery for rewiring is also highly active in this brain part under a social setting. The GO term neuromast deposition was also present in the diencephalon, and the GO term formation of eye tissue was also detected in the brain stem, suggesting that both brain parts are also involved in sensory perception. Amongst the most highly DE genes in the brainstem were the hox genes (Table S1) which play an important role during the formation of the bodyplan in embryos. These genes remain expressed in the hindbrain of adult mouse (Zapala et al., 2005) and were also the most upregulated genes in the cerebellum/hindbrain of sticklebacks under a social setting (Greenwood and Peichel, 2015). We show that the hox genes are 70–191 times upregulated in the brain stem compared to the average expression in the remaining brain parts (Table S1) while no significant differential regulation of these genes is present in the cerebellum. Moreover, the number of reads mapped to these genes was between 0 and 2 in the non brain stem samples, which shows that they are mainly expressed in the brain stem and not in the cerebellum.

### Gene expression differences between brain parts are conserved across species

Our analyses show concordant patterns between the two species in terms of differences in fold changes (Figure 1, Figure S2), number of DE genes (Figure 2), and enriched biological processes (Figure 3, Figure S3). Our statistical analyses identified only 32 genes with a significant difference in fold change between species. These genes were involved in basic biological processes related to transcription and transport of molecules between cells (Table S3) and indicate that the neural networks and functioning of the brain in both species are consistent. In addition to the small number of significant interaction genes, we found a large proportion of genes (on average 86% genes) with an equivalent logFC in each contrast in both species, showing that gene expression differences between brain parts are conserved across species.

### Expression of genes linked to fish behavior across the female brain under a social setting

Immediate early genes (IEG) play an important role in gene x environment interactions because they rapidly respond to environmental cues and provide the possibility of lasting adaptation to new environmental conditions through the regulation of genes involved in neuronal activity and plasticity (Herdegen and Leah, 1998). IEG show a basal expression in many brain regions, and particularly so where neurons recieve ongoing synaptic input (Herdegen and Leah, 1998). Our results indeed show that *egr1, bdnf*, *fosb*, and *fosl2* are expressed throughout the female brain and obtain high gene expression in the olfactory bulbs (Figure S5). *Egr1* is a transcription factor encoding gene that allows a rapid response to social opportunities in birds (Mello et al., 1992) and cichlids. In addition, *egr1* and *bdfn* have been linked to learning and performing a spatial task and were significantly more expressed in the telencephalon in the cichlid *A. burtoni* (Wood et al., 2011) which agrees with our results. *egr1* mediates the transcription of the *gnrh1* gene in the gonadotropin-releasing-containing neurons located in the hypothalamic–preoptic area of the brain (Fernald and Maruska, 2012). Our results show that *gnrh1* and *gnrh3* are expressed in the olfactory bulbs and to a lesser extent in the telencephalon. This is in agreement with the forebrain localisation of both hormones in sea bass (Gonzalez-Martinez et al., 2001).

Two hormones are known to play an important role in social behavior in fishes and other vertebrates: arginine vasotocin (*avpi1*) and isotocin (*oxt*). These are both expressed in the preoptic area of the anterior hypothalamus (located in the diencephalon) in fish (Godwin and Thompson, 2012) and in the hypothalamus of human (Sukhov et al., 1993). Our results show that the expression of vasotocin (*avpi1*) in *Ophthalmotilapia* occurs at low levels in all brain parts, and significantly more in the cerebellum than in the other brain parts (Figure S5). Isotocin on the other hand was highly and consistently expressed only in the brainstem of both species (Figure S5). This is different than gene expression of oxytocin (the vertebrate homolog of fish isotocin) in the homolog of the telencephalon and diencephalon in rodents (Bosch, 2013) and in the homolog of the diencephalon in humans (Sukhov et al., 1993; Bosch, 2013). *Vip* (vasoactive intestinal peptide) has shown differential expression in the cerebellum of stickleback males and its expression is regulated in opposite directions during courtship and a territorial challenge (Sanogo and Bell, 2016). In contrast, *Vip* was not expressed in the cerebellum of chick but was detected in several other brain parts, with highest expression levels in the diencephalon (Kuenzel et al., 1997). Our results show that *Vip* was also not expressed in the cerebellum of *Ophthalmotilapia* females, and was significantly more expressed in the brainstem and the diencephalon. Neuroserpin and neuroligin modulate synaptic plasticity and synaptogenesis in the human brain (Dean and Dresbach, 2006; Miranda and Lomas, 2006), and play a role in female mate choice of swordtails (Cummings et al., 2008). Neuroserpin is especially expressed in the human homologs of the fish telencephalon, diencephalon and cerebellum (Miranda and Lomas, 2006) and is believed to play a key role in memory and learning. We find that both genes are expressed in all brain regions of the two ciclid species, and that neuroserpin was significantly more expressed in the telencephalon, diencephalon and olfactory bulbs.

Since behavioral patterns and adaptation to new environments may be linked to receptor activity in the brain rather than to signaling activity (Di Poi et al., 2016), we screened the expression of 16 receptor genes involved in four physiological regulatory networks. Two serotonergic receptors (*htr1a* and *htr2a*), one dopaminergic receptor (*drd2*) and one adrenergic receptor (*adrb1*) are expressed in all parts of the female brain, except for the cerebellum. In this brain part, there was a strong expression of the *adrb1* gene, but the other receptors were not expressed (Figure S4). The adrenergic system regulates anxiety and social behavior through adrenergic receptors, which form the bridge between the sympathic nervous system and the cardivascular system and with many endocrine and parenchymal tissues in animal *in vitro* systems (Hein and Kobilka, 1997). In agreement with our results, a high density of the adrenergic beta receptors have been documented in the cerebellum and diencephalon of the zebrafish (Ampatzis and Dermon, 2016).

## Conclusion

The results of our study demonstrate substantial differential gene expression between all six brain parts which are consistent between two closely related cichlid species that have similar morphology and behavior. The detection of differential gene expression in the different brain parts of closely related species greatly improves our understanding of the genetic basis of the functional differences of different brain regions. We also detected several genes linked to fish behavior under our control setting. The pronounced differences between brain regions indicate that understanding the interactions between genes and behavioral responses in *Ophthalmotilapia* will be enhanced by neurogenomic profiling of the separate brain regions.

## Data accesibility

RNA sequences: the data presented in this publication have been deposited in NCBI's Gene Expression Omnibus (Edgar et al., 2002) and are accessible through GEO Series accession number GSE109106 (https://www.ncbi.nlm.nih.gov/geo/query/acc.cgi?acc=GSE106109). R script: see Datasheet 1 in online Supporting Information.

## Author contributions

EV, SD, JV, KH, EP, PP, LK. and MV concieved the idea, SD, KH, JV, and LK optimized the methodology, JV and KH generated the sequencing data and optimized the bioinformatic pipeline for filtering and mapping the reads, SD, KV, and LC analyzed the data, KV and LC performed the statistical analyses, SD wrote the manuscript. All authors contributed critically to the drafts and gave final approval for publication.

### Conflict of interest statement

The authors declare that the research was conducted in the absence of any commercial or financial relationships that could be construed as a potential conflict of interest.

## Supplementary material

Table S1 List of genes that are DE in both species with a |log2FC| > 2. Both the locus name (Locus) and gene ID (GeneID) from Oreochromis niloticus of each gene is provided, along with the gene ontology ID (GoID) and Biological Process. LogFC and adjusted p-values after the stage-wise statistical analyses for O. nasuta (Na) and O. ventralis (Ve) are provided. ‘Brainpart’ refers to the brainpart in which the DE of the gene was found.

Table S2 GO terms for each brainpart identified by CAMERA as significantly enriched. For each brainpart (BS, CE, TE, OT, OB, DI), the GO term, Biological process, adjusted P-value for O. nasuta (adj Pvalue ON) and for O. ventralis (adj Pvalue OV) are provided. GO terms and Biological processes in bold are uniquely enriched in that particular brainpart.

Table S3 Genes that are significantly different for the interaction brain part ^*^ species after the stage wise testing procedure. Gene, Locus name Oreochromis niloticus; GeneID, GeneID Oreochromis niloticus; log2 FC, log fold chance; adj P value, adjusted P value according to Holm; contrast, contrast compared between O. nasuta and O. ventralis; go_id, go_id retrieved using Biomart; Biological Process, biological process the gene is involved in; NA, not available.

Table S4 Log2FC represent the gene expression in each brainpart compared to the average gene expression in the remaining 5 brainparts. Genes that are significantly differentially expressed in both species are indicated with LogFC and AdjP values in bold. Values in bold indicate genes that are significantly more expressed compared to the average gene expression in the other brainparts in both species. not expressed' means that the genes were not detected at all in the current dataset. blank lines indicate that the gene was not DE or was not expressed in that particular brainpart/species.

Figure S1 STAR alignment scores of the reads for each of the 60 samples. All reads were mapped against the Oreochromis nilo-cus genome version ASM185804v2. Green: % of reads uniquely mapped; Blue: % of reads mapped to mulJple loci; orange: % of reads not mapped because the read was too short; Red: % of reads not mapped for other reasons (eg because they were too different from the reference genome). On average, 53.4% of the reads uniquely mapped against the O. nilo-cus genome and only a small fracJon (2.5 – 5.3 %) of the reads were mapped to mulJple loci.

Figure S2 Heatmap of log2 values of cpm from the top 200 genes that are differentially expressed in one brain part compared to the average expression in the other five brain parts for O. nasuta and O. ventralis. Rows represent genes, columns represent brain parts. (Na: O. nasuta; Ve: O. ventralis; BS, brainstem; CE, cerebellum; DI, diencephalon; OB, olfactory bulb; OT, optic tectum; TE, telencephalon; 1-5: biological replicates).

Figure S3 Bubble plots of significant GO terms determined by CAMERA. Plots for each brainpart (BS, OB, CE, OT, DI and TE) are side by side for the two species (Na or Ve). Only significant GO terms are labelled. The Y-axis represents the significance of the GO term (the higher the value in the plot, the more significant the term is), the X-axis shows the Z-score, a measure that indicates whether the biological process is more likely to be decreased (negaSve values) or increased (posiSve values), circles represent GO terms and the size of the circle illustrates the number of genes included in the GO term. The yellow line demarcates the 5% significance level.

Figure S4 Log2 cpm values of the 32 interaction genes in each brain part for O. nasuta (Black) and O. ventralis (Red).

Figure S5 Number of reads in each of the 59 sample safter quality filtering for four immediate early genes (bdnf, egr1, fosb, fosl2), nine behavioral genes (avpi1, gabarap, gnrh1, gnrh3, kpna1, nlgn3, oxt, serpini1, vip) and four receptor genes (adrb1, drd2, htr1a, htr2a) reported in previous studies (see references in Table S4). Note that the Y-axis has different limits between genes to enhance visualisation.
