# Supplementary material for: Neurogenomic Profiling Reveals Distinct Gene Expression Profiles Between Brain Parts That Are Consistent in Ophthalmotilapia Cichlids
Source: Front Neurosci. 2018 Mar 9;12:136. doi: 10.3389/fnins.2018.00136 (PMC5855355; doi:10.3389/fnins.2018.00136)
Supplement: Figure S1 — STAR alignment scores of the reads for each of the 60 samples. All reads were mapped against the Oreochromis nilo-cus genome version ASM185804v2. Green: % of reads uniquely mapped; Blue: % of reads mapped to mulJple loci; orange: % of reads not mapped because the read was too short; Red: % of reads not mapped for other reasons (eg because they were too different from the reference genome). On average, 53.4% of the reads uniquely mapped against the O. nilo-cus genome and only a small fracJon (2.5 – 5.3 %) of the reads were mapped to mulJple loci. [file Image1.PDF]

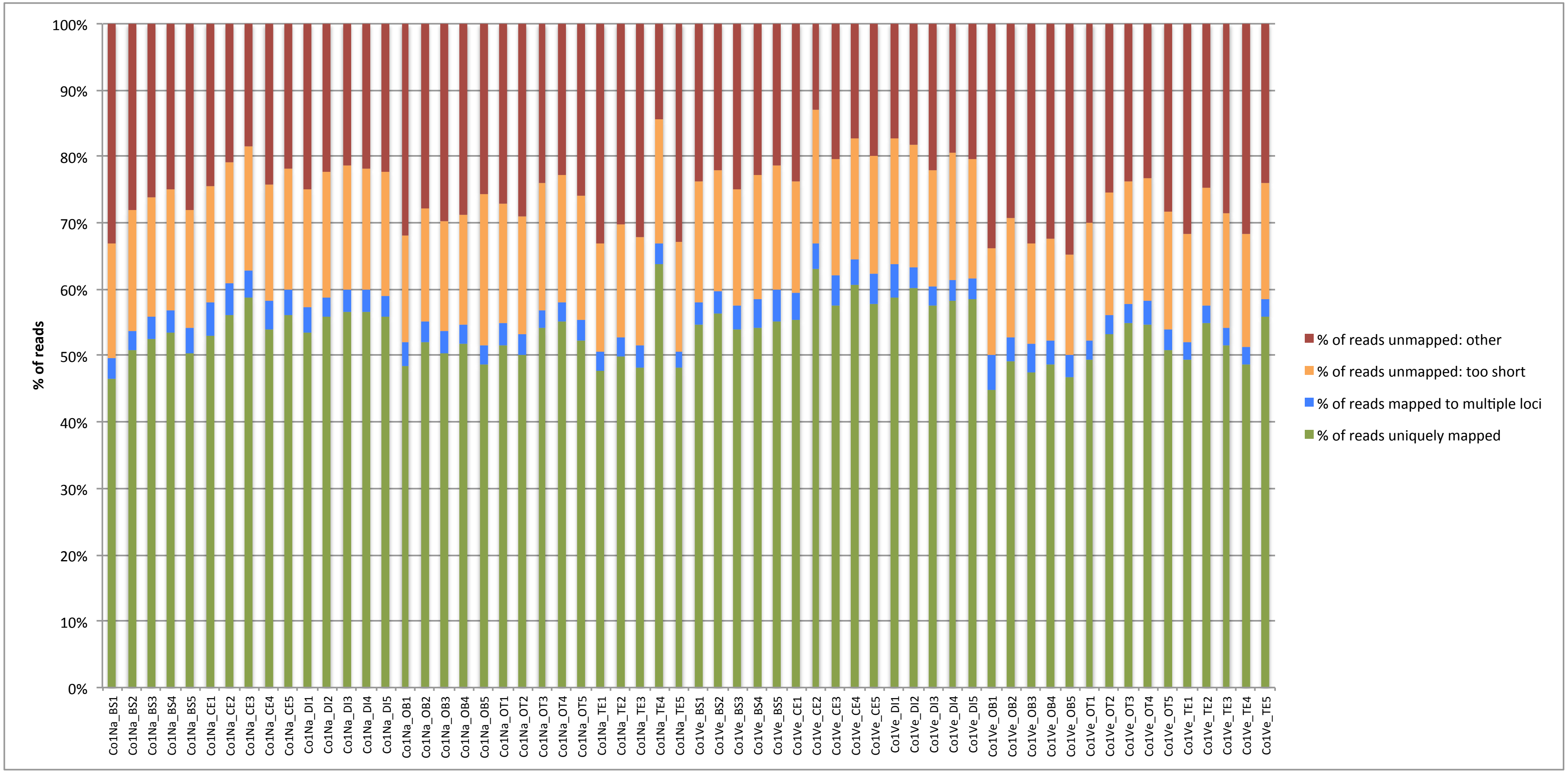

Fig S1: STAR alignment scores of the reads for each of the 60 samples. All reads were mapped against the *Oreochromis niloticus* genome version ASM185804v2. Green: % of reads uniquely mapped; Blue: % of reads mapped to multiple loci; orange: % of reads not mapped because the read was too short; Red: % of reads not mapped for other reasons (eg because they were too different from the reference genome). On average, 53.4% of the reads uniquely mapped against the *O. niloticus* genome and only a small fraction (2.5 – 5.3 %) of the reads were mapped to multiple loci
