# Supplementary figures and images for: Neurogenomic Profiling Reveals Distinct Gene Expression Profiles Between Brain Parts That Are Consistent in Ophthalmotilapia Cichlids
Source: Front Neurosci. 2018 Mar 9;12:136. doi: 10.3389/fnins.2018.00136 (PMC5855355; doi:10.3389/fnins.2018.00136)

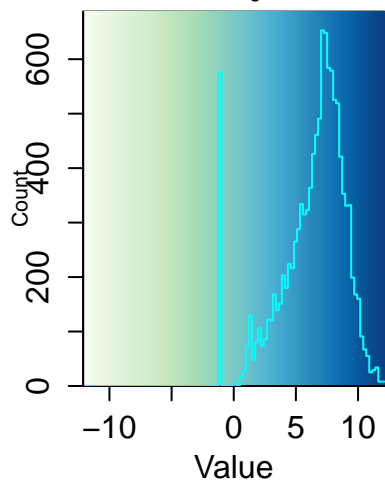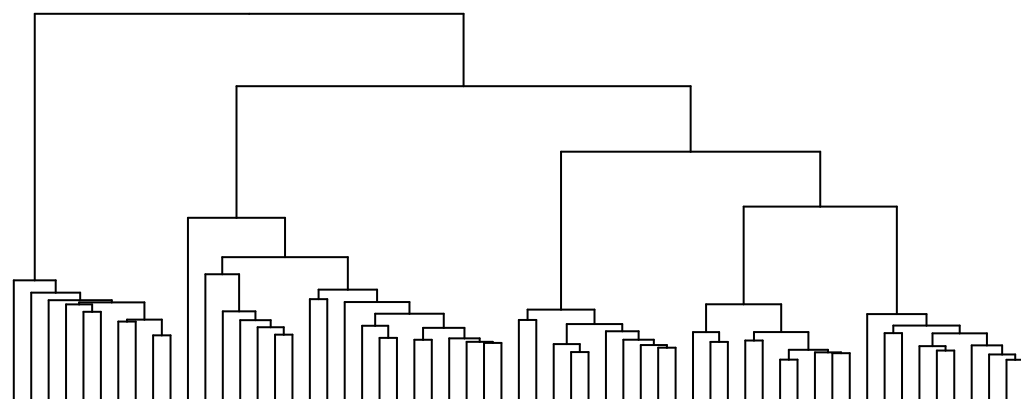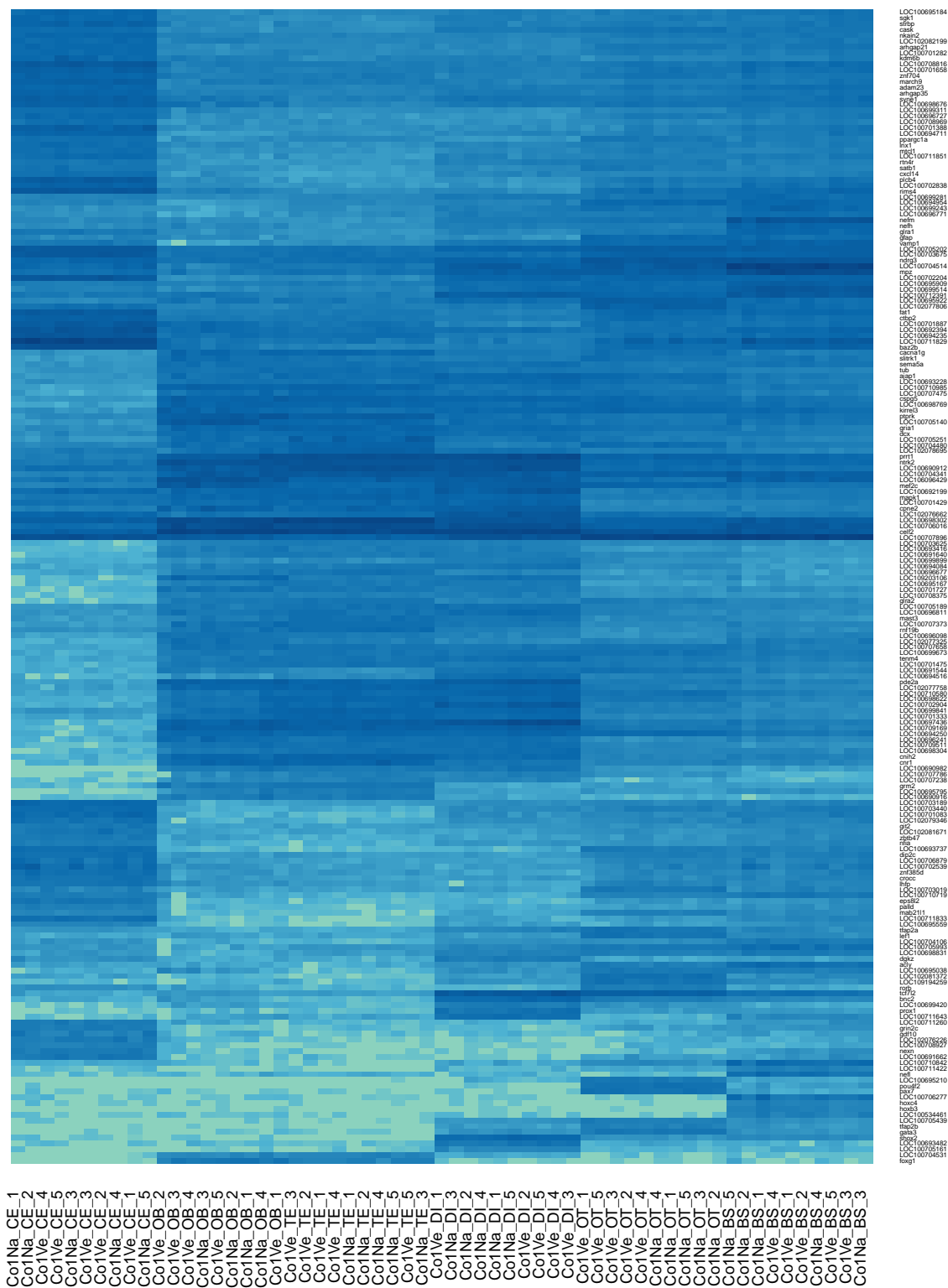

Supplement: Figure S2 — Heatmap of log2 values of cpm from the top 200 genes that are differentially expressed in one brain part compared to the average expression in the other five brain parts for O. nasuta and O. ventralis. Rows represent genes, columns represent brain parts. (Na: O. nasuta; Ve: O. ventralis; BS, brainstem; CE, cerebellum; DI, diencephalon; OB, olfactory bulb; OT, optic tectum; TE, telencephalon; 1-5: biological replicates). [file Image2.PDF]
