# Supplementary material for: Neurogenomic Profiling Reveals Distinct Gene Expression Profiles Between Brain Parts That Are Consistent in Ophthalmotilapia Cichlids
Source: Front Neurosci. 2018 Mar 9;12:136. doi: 10.3389/fnins.2018.00136 (PMC5855355; doi:10.3389/fnins.2018.00136)
Supplement: Figure S3 — Bubble plots of significant GO terms determined by CAMERA. Plots for each brainpart (BS, OB, CE, OT, DI and TE) are side by side for the two species (Na or Ve). Only significant GO terms are labelled. The Y-axis represents the significance of the GO term (the higher the value in the plot, the more significant the term is), the X-axis shows the Z-score, a measure that indicates whether the biological process is more likely to be decreased (negaSve values) or increased (posiSve values), circles represent GO terms and the size of the circle illustrates the number of genes included in the GO term. The yellow line demarcates the 5% significance level. [file Image3.PDF]

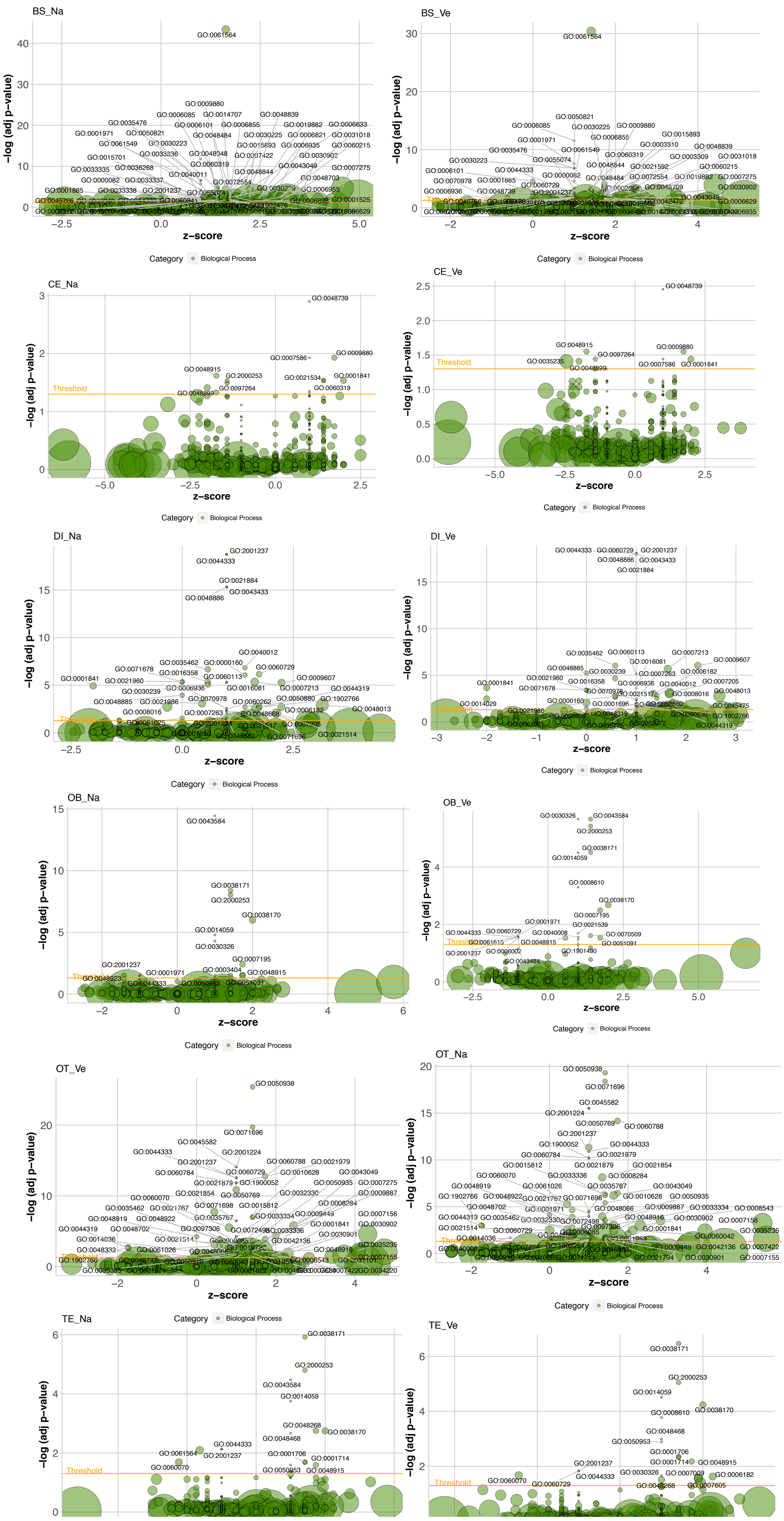

Fig 3. Bubble plots of significant GO terms determined by CAMERA. Plots for each brainpart (BS, OB, CE, OT, DI and TE) are side by side for the two species (Na or Ve). Only significant GO terms are labelled. The Y-axis represents the significance of the GO term (the higher the value in the plot, the more significant the term is), the X-axis shows the Z-score, a measure that indicates whether the biological process is more likely to be decreased (negative values) or increased (positive values), circles represent GO terms and the size of the circle illustrates the number of genes included in the GO term. The yellow line demarcates the 5% significance level.
