# Supplementary material for: Neurogenomic Profiling Reveals Distinct Gene Expression Profiles Between Brain Parts That Are Consistent in Ophthalmotilapia Cichlids
Source: Front Neurosci. 2018 Mar 9;12:136. doi: 10.3389/fnins.2018.00136 (PMC5855355; doi:10.3389/fnins.2018.00136)
Supplement: Figure S4 — Log2 cpm values of the 32 interaction genes in each brain part for O. nasuta (Black) and O. ventralis (Red). [file Image4.PDF]

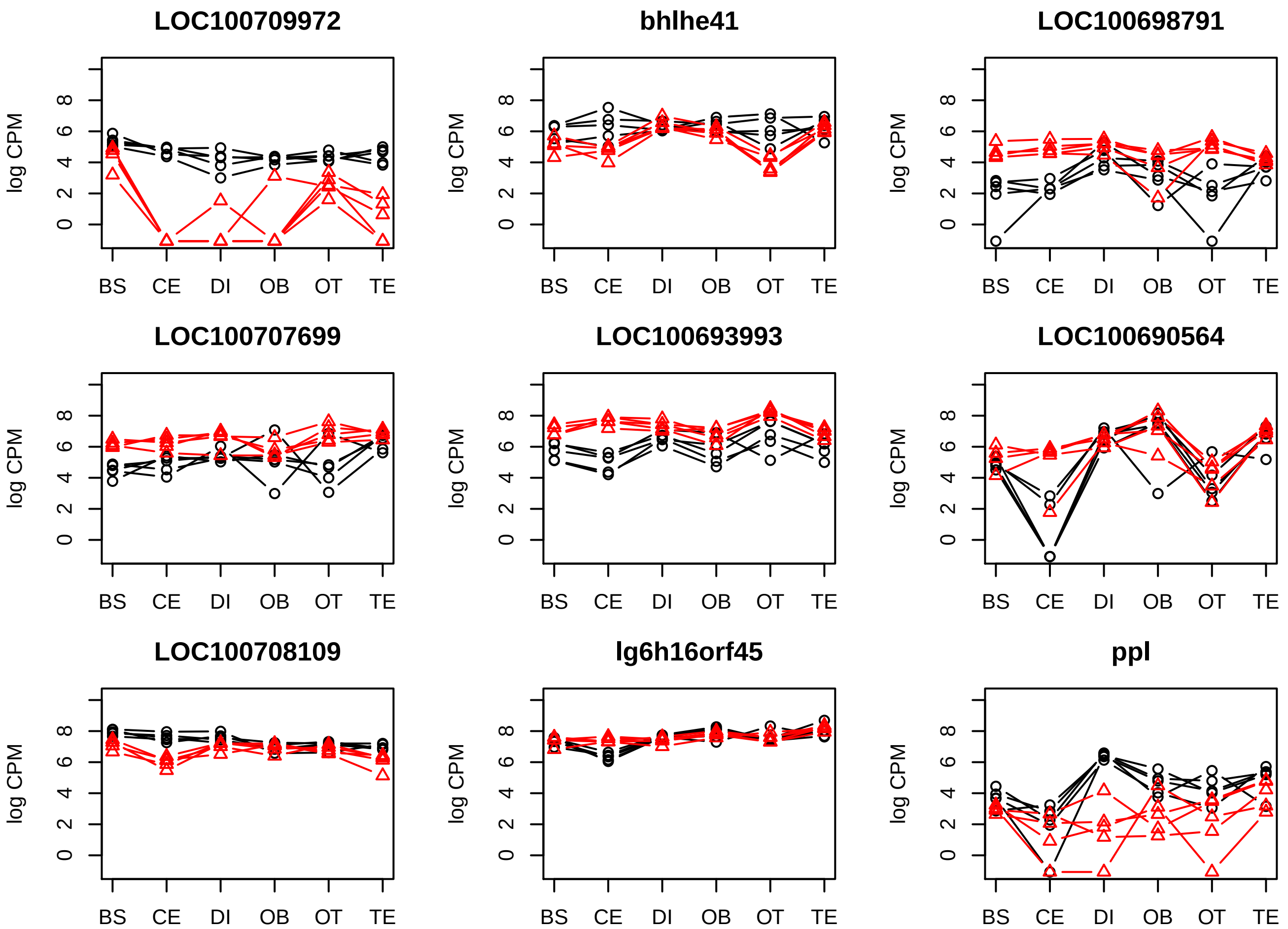

LOC100709972
bhlhe41
LOC100698791
LOC100707699
LOC100693993
LOC100690564
LOC100708109
lg6h16orf45
ppl
log CPM
0
2
4
6
8
BS
CE
DI
OB
OT
TE

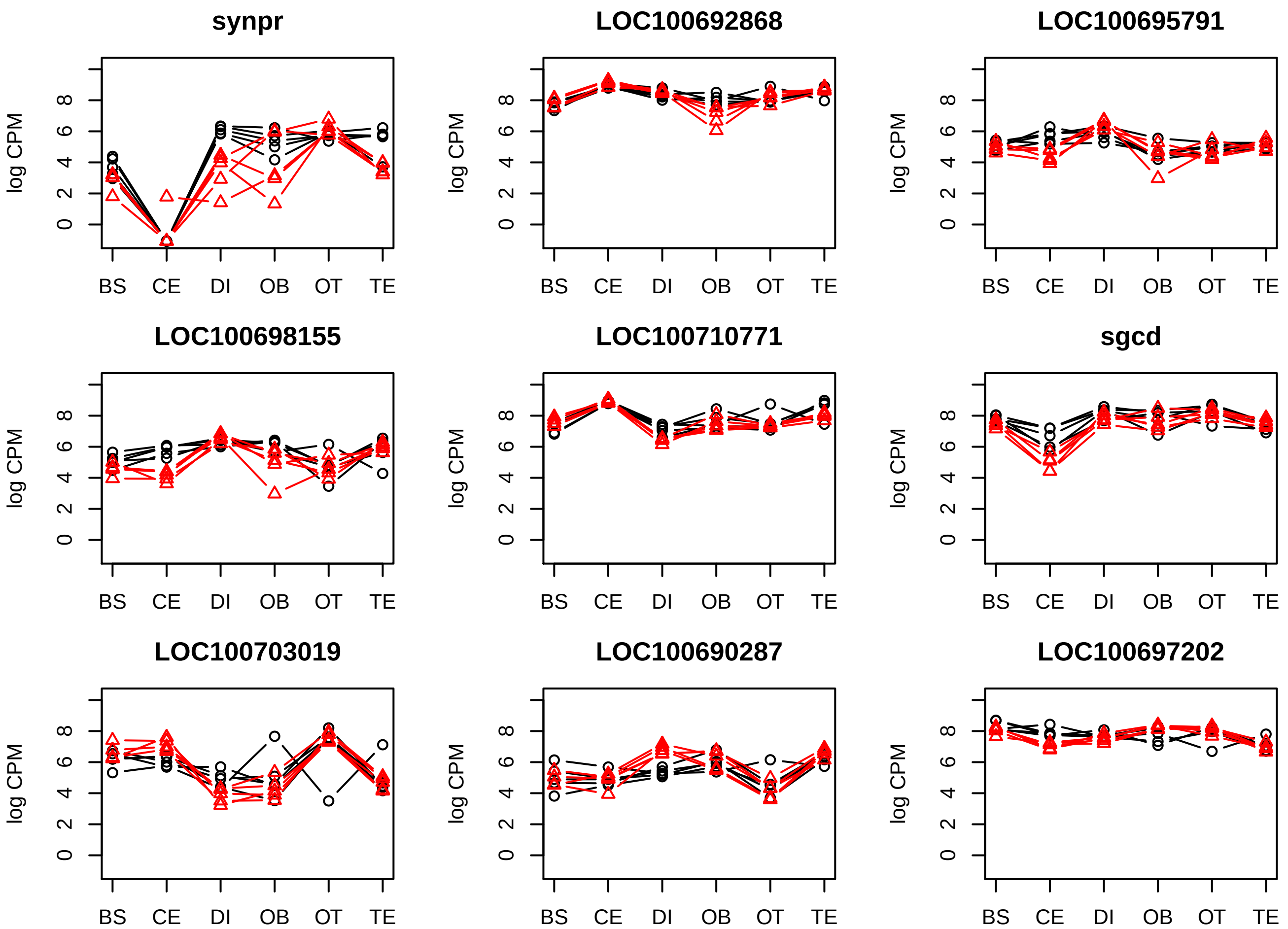
synpr
LOC100692868
LOC100695791
LOC100698155
LOC100710771
sgcd
LOC100703019
LOC100690287
LOC100697202
log CPM
0 2 4 6 8
BS CE DI OB OT TE

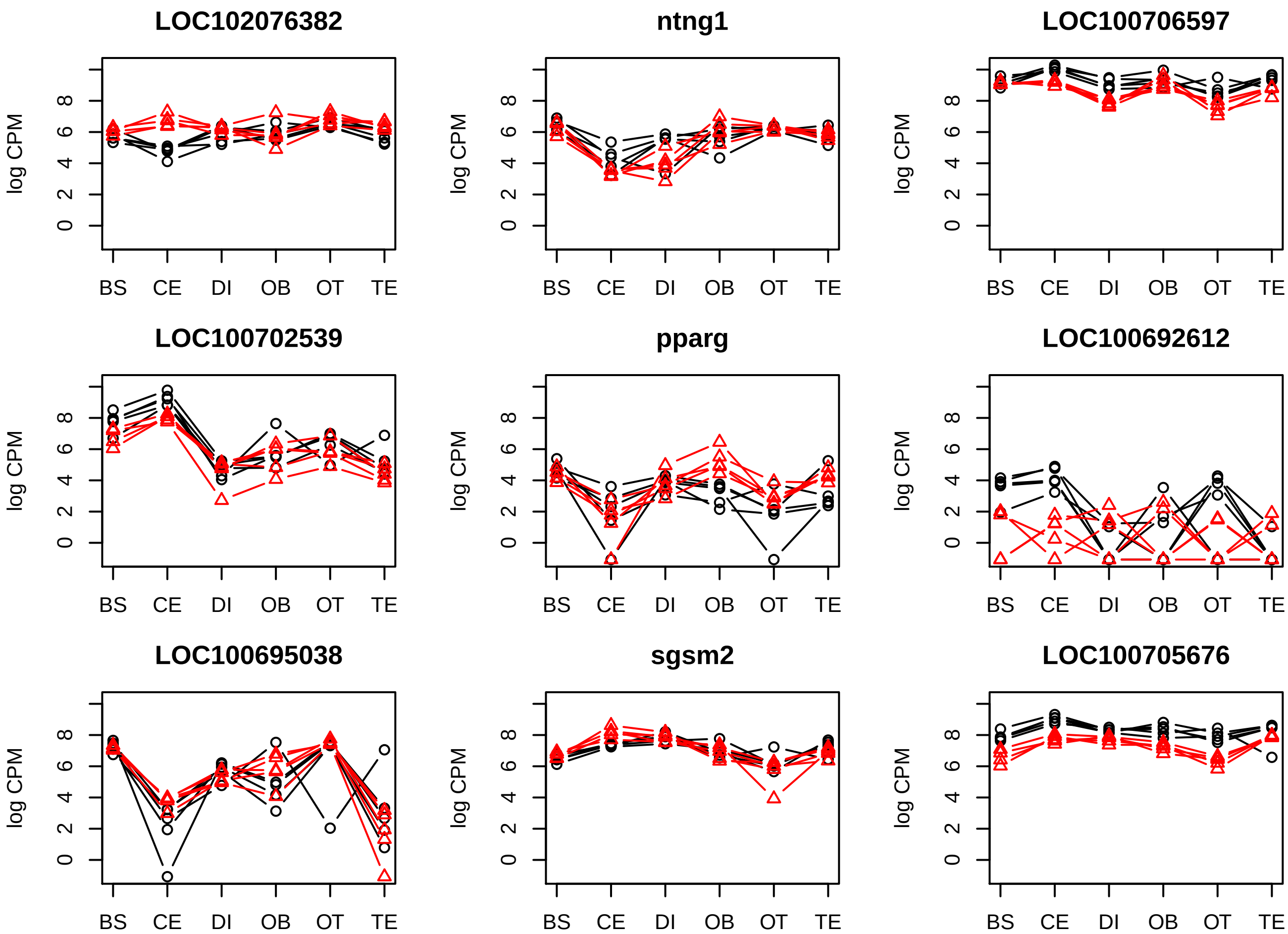

LOC102076382
ntng1
LOC100706597
LOC100702539
pparg
LOC100692612
LOC100695038
sgsm2
LOC100705676
log CPM
0
2
4
6
8
BS
CE
DI
OB
OT
TE

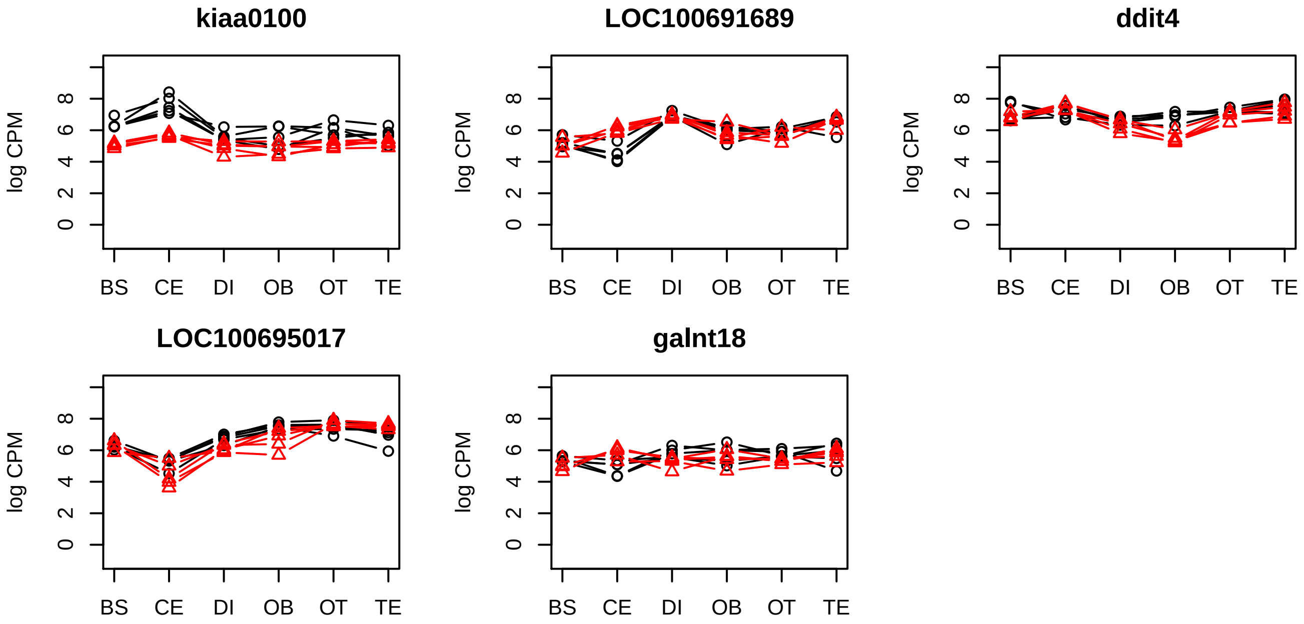

kiaa0100
LOC100691689
ddit4
LOC100695017
galnt18
log CPM
0
2
4
6
8
BS
CE
DI
OB
OT
TE
