# Supplementary material for: Neurogenomic Profiling Reveals Distinct Gene Expression Profiles Between Brain Parts That Are Consistent in Ophthalmotilapia Cichlids
Source: Front Neurosci. 2018 Mar 9;12:136. doi: 10.3389/fnins.2018.00136 (PMC5855355; doi:10.3389/fnins.2018.00136)
Supplement: Figure S5 — Number of reads in each of the 59 sample safter quality filtering for four immediate early genes (bdnf, egr1, fosb, fosl2), nine behavioral genes (avpi1, gabarap, gnrh1, gnrh3, kpna1, nlgn3, oxt, serpini1, vip) and four receptor genes (adrb1, drd2, htr1a, htr2a) reported in previous studies (see references in Table S4). Note that the Y-axis has different limits between genes to enhance visualisation. [file Image5.pdf]

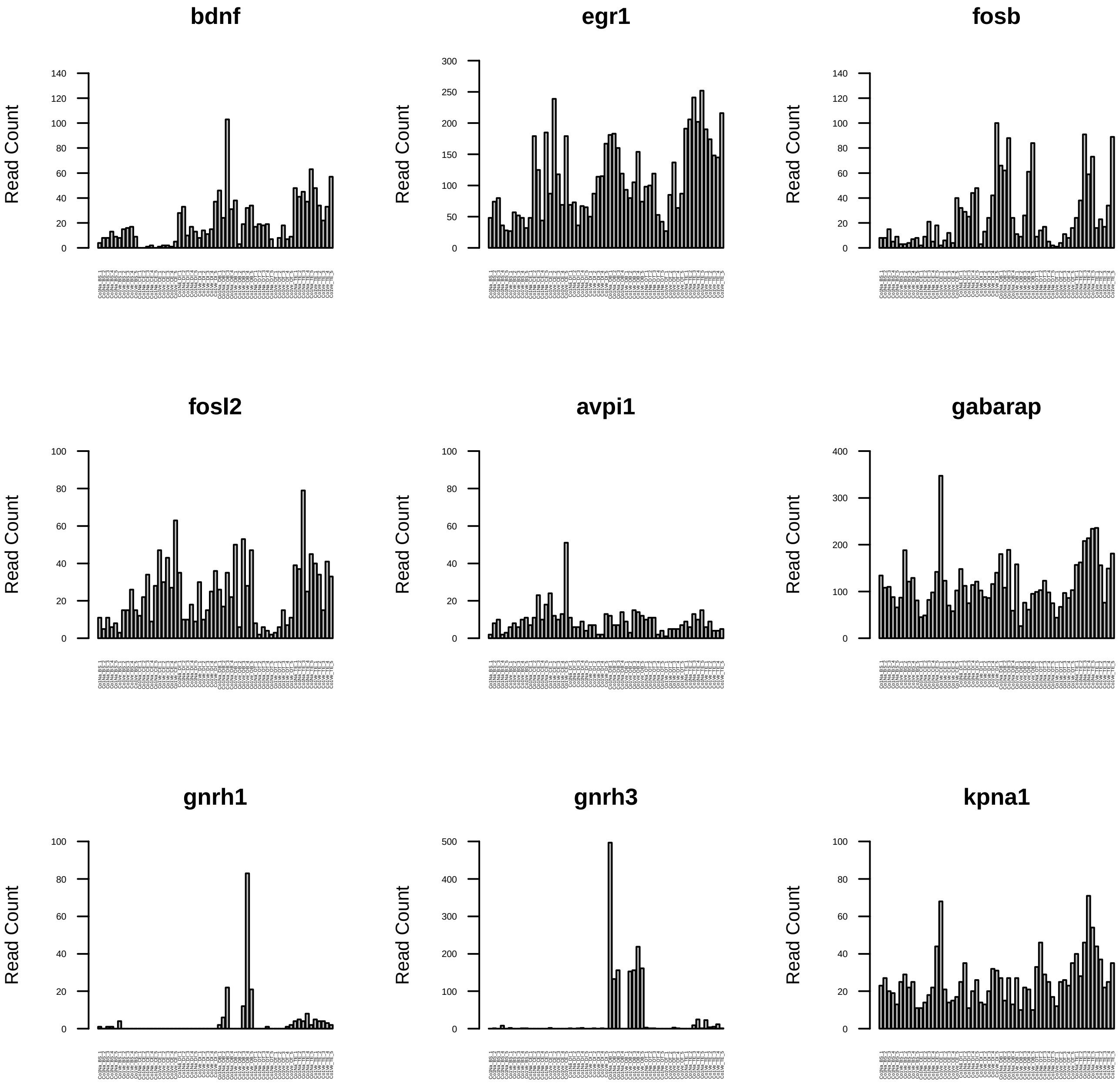
bdnf
egr1
fosb
fosl2
avpi1
gabarap
gnrh1
gnrh3
kpna1
Read Count
0
20
40
60
80
100
120
140
50
150
200
250
300
400
500

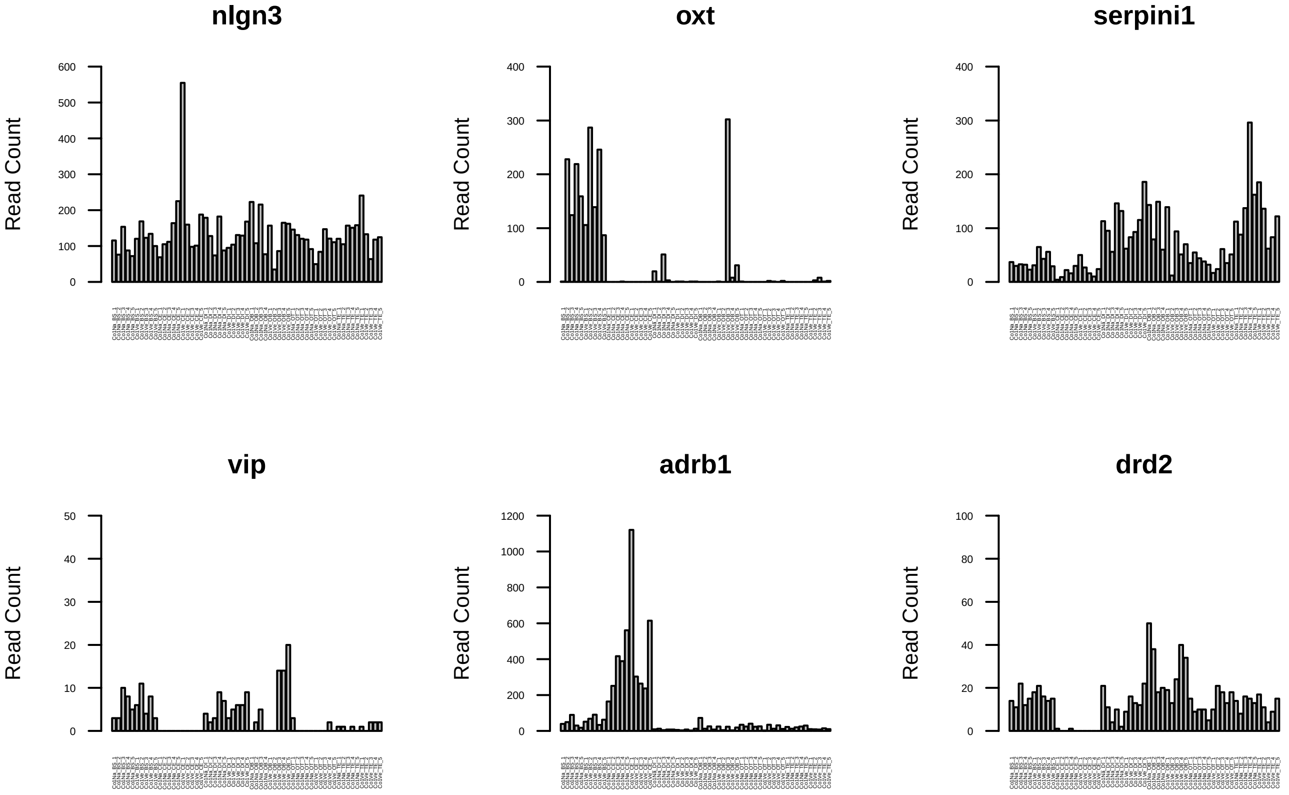

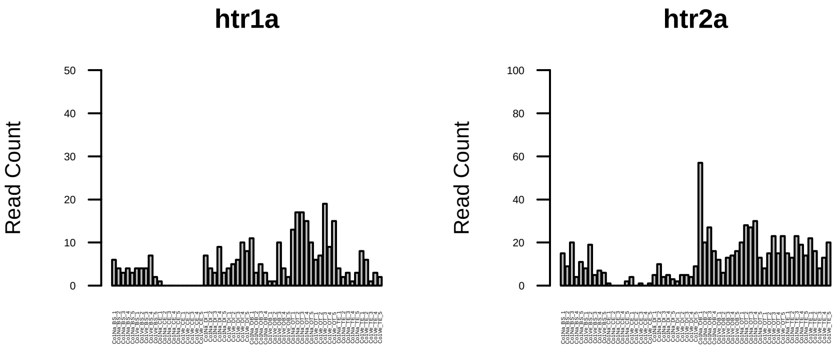

Fig S5. Number of reads in each of the 59 samples after quality filtering for four immediate early genes (bdnf, egr1, fosb, fosl2), nine behavioral genes (avpi1, gabarap, gnrh1, gnrh3, kpna1, nlgn3, oxt, serpini1, vip) and four receptor genes (adrb1, drd2, htr1a, htr2a) reported in previous studies (see references in Table S4). Note that the Y-axis has different limits between genes to enhance visualisation.
