# Supplementary material for: Neurogenomic Profiling Reveals Distinct Gene Expression Profiles Between Brain Parts That Are Consistent in Ophthalmotilapia Cichlids
Source: Front Neurosci. 2018 Mar 9;12:136. doi: 10.3389/fnins.2018.00136 (PMC5855355; doi:10.3389/fnins.2018.00136)
Supplement: Supplementary file 10 [file DataSheet1.DOCX]

This script has been used in EdgeR v 3.12

The dataset consists of 2 cichlid fish species (0. nasuta, O. ventralis) from which 6 parts of the brain had been sequenced over 5 biological replicates. This provides us with 60 samples.

We address two hypotheses:

H0: there is no DE of genes between a brainpart and the average expression in the five other brainparts within each species

H0: there is no difference in expression pattern across brainparts between the two species

We generate a contrastmatrix for each of these two hypotheses.

```{R,echo=FALSE}

library(edgeR)

library(gplots)

library(RColorBrewer)

library(stageR)

library(limma)

library(VennDiagram)

```

# Read in data

sessionInfo()

```{r}

dir="/Users/sderycke/Documents/Genbas/RNA/Control_Females/OniloticusUMD1_103/O_niloticus/count_files/"

files=list.files(dir)

files=files[grep(x=files,pattern="^Co")]

d=readDGE(paste0(dir,files), nrows=36499,header=FALSE)

sampleNames=unlist(lapply(strsplit(colnames(d),split="/"),function(x) x[11]))

species=factor(substr(sampleNames,4,5))

brain=factor(unlist(lapply(strsplit(sampleNames,split="_"),function(x) x[2])))

specimen=as.numeric(substr(unlist(lapply(strsplit(sampleNames,split="_"),function(x) x[3])),1,1))

specimen[31:60]=specimen[31:60]+5

specimen=factor(specimen)

colnames(d)=unlist(lapply(strsplit(sampleNames,split=".",fixed=TRUE),function(x) x[1]))

```

# Remove outlying sample

One sample (Co1Na_OB_5) has an extremely low library size: the olfactory bulb from the fifth specimen of the O. nasuta species. We remove this sample, and accordingly change the metadata.

```{r}

barplot(d$samples$lib.size/1e6, ylab="Library size (millions)", names.arg = colnames(d), cex.names = 0.4, las=2)

keepSample=!colnames(d)=="Co1Na_OB_5"

species=species[keepSample]

brain=brain[keepSample]

specimen=specimen[keepSample]

d=d[,keepSample]

dim(d)

```

# Filtering, normalization, exploratory data analysis

We filter genes that do not have a CPM of 15 in at least 4 samples. We picked 4 samples because this is the lowest number of samples for any treatment group (brain). For the lowest library size of 137528, a CPM of 15 corresponds to a count of (15/1e6)*137528 = ca 2 in the lowest sample.After this filter, 11577 genes are kept.

```{r}

min(d$samples$lib.size)

keep <- rowSums(cpm(d)>15)>=4

table(keep)

d=d[keep,,keep.lib.sizes=FALSE]

barplot(d$samples$lib.size/1e6, ylab="Library size (millions)", names.arg = colnames(d), cex.names = 0.4, las=2)

d=calcNormFactors(d)

```

## Gene expression data exploration

to count the number of genes expressed per sample, shared between all samples and occurring in only one sample;

```{r}

NumberGenes = colSums(d$counts != 0)

median(NumberGenes)

setEPS()

postscript(paste("Number of Genes.eps", sep = ""))

barplot(NumberGenes, ylab="Number of genes", ylim = c(0, 12000),names.arg = colnames(NumberGenes), cex.names = 0.4, las=2, )

dev.off()

NumberGenesShared = as.data.frame(rowSums(d$counts != 0))

sum(NumberGenesShared == 59) #7224

sum(NumberGenesShared == 1) #0

```

##The MDS plot clearly shows that the samples cluster by brain, suggesting the largest variability in expression can be explained by brain part.

```{r}

setEPS()

pch = c(8,19)

cols = c("firebrick", "skyblue", "pink3", "darkseagreen", "darkgoldenrod", "mediumpurple")

postscript(paste("MDSplot.eps", sep = ""))

plotMDS(d, pch = pch[species] , col= cols[as.double(brain)], cex = 1.5)

legend('topright', legend = c("O. nasuta", "O. ventralis", "BS", "CE", "DI", "OB", "OT", "TE"), pch = c(8,19,1,1,1,1,1,1), col=c("black", "black", "white", "white", "white", "white", "white", "white"), text.col = c("black", "black", "firebrick", "skyblue", "pink3", "darkseagreen", "darkgoldenrod", "mediumpurple"), cex = 0.8)

dev.off()

```

# Differential expression analysis

We use the Negative binomial model which takes care of multiple factors by fitting generalized linear models (GLM) with a design matrix. We first have to create the design matrix based on our samples.

## Design matrix

We have specimen effects (brainparts within a specimen are linked to each other) and brainpart effects. We first make a matrix with the parameters for the specimens, and then we make a matrix with the parameters for the brainparts.Columns are specimens and brainparts, rows are the samples. Both matrices are then merged to generate the design matrix

```{r}

specimenHlp=model.matrix(~-1+specimen)

colnames(specimenHlp)=paste0("s",1:10)

rownames(specimenHlp)=colnames(d)

specimenHlp

brainSpecies=factor(paste(brain,species,sep="_"))

brainHlp=model.matrix(~-1+brainSpecies)

colnames(brainHlp)=levels(brainSpecies)

brainHlp

```

#We have separate specimen effect so we can only estimate 4 brain parameters within species. We drop first two brain parameters, BS in Na and BS in Ve. The intercept in our model will be the average expression in BS over the specimens (5 for Na, 5 for Ve). Note, that we can estimate the BS effect in Ve if we take the average over all Ve specimen parameters.

```{r}

design_2way= cbind(specimenHlp,brainHlp[,-(1:2)])

design_2way

```

## Dispersion estimation and GLM fitting

The negative binomial dispersion is estimated using the estimateDisp function. This returns the DGEList object with additional entries for the estimated NB dispersions for all genes. These estimates include the common, trended and tagwise dispersion estimates, which can be visualized with plotBCV, which shows the root-estimate, i.e., the biological coefficient of variation for each gene.

```{r}

d=estimateDisp(d,design_2way,robust=TRUE)

d$common.dispersion

plotBCV(d)

fit_2way = glmQLFit(d, design_2way, robust=TRUE)

head(fit_2way$coefficients)

plotQLDisp(fit_2way)

```

## III.4. Testing for differential expression

## III.4.2. Hypothesis 1: There is no differential expression between a brain part and the average of all other brain parts in O. nasuta and O. ventralis.

### III.4.2.1. Screening stage

We will first make a contrast matrix containing all contrasts of interest.The rows in the contrastmatrix are the colnames of the designmatrix, and the columns are the contrasts of interest.

Then we use this matrix to perform the screening step, testing whether any of the contrasts of interest are false.

In the first block of code we build a contrast matrix that involves all contrasts within one species. Note, that the average expression of the BS brain part for a species is obtained by averaging the specimen effects for the specimens belonging to that species.

```{r}

## all contrasts within one species.

LBrain=matrix(0,nrow=ncol(fit_2way$coefficients),ncol=12)

rownames(LBrain)=colnames(fit_2way$coefficients)

colnames(LBrain)=c("BS-avg_Na","BS-avg_Ve",

"CE-avg_Na","CE-avg_Ve",

"DI-avg_Na","DI-avg_Ve",

"OB-avg_Na","OB-avg_Ve",

"OT-avg_Na","OT-avg_Ve",

"TE-avg_Na","TE-avg_Ve")

#O. nasuta

LBrain[c("CE_Na","DI_Na","OB_Na","OT_Na","TE_Na"),"BS-avg_Na"]=-1/5

LBrain[c("CE_Na","DI_Na","OB_Na","OT_Na","TE_Na"),"CE-avg_Na"]=c(1,rep(-1/5,4))

LBrain[c("DI_Na","CE_Na","OB_Na","OT_Na","TE_Na"),"DI-avg_Na"]=c(1,rep(-1/5,4))

LBrain[c("OB_Na","CE_Na","DI_Na","OT_Na","TE_Na"),"OB-avg_Na"]=c(1,rep(-1/5,4))

LBrain[c("OT_Na","CE_Na","DI_Na","OB_Na","TE_Na"),"OT-avg_Na"]=c(1,rep(-1/5,4))

LBrain[c("TE_Na","CE_Na","DI_Na","OB_Na","OT_Na"),"TE-avg_Na"]=c(1,rep(-1/5,4))

#O. ventralis

LBrain[c("CE_Ve","DI_Ve","OB_Ve","OT_Ve","TE_Ve"),"BS-avg_Ve"]=-1/5

LBrain[c("CE_Ve","DI_Ve","OB_Ve","OT_Ve","TE_Ve"),"CE-avg_Ve"]=c(1,rep(-1/5,4))

LBrain[c("DI_Ve","CE_Ve","OB_Ve","OT_Ve","TE_Ve"),"DI-avg_Ve"]=c(1,rep(-1/5,4))

LBrain[c("OB_Ve","CE_Ve","DI_Ve","OT_Ve","TE_Ve"),"OB-avg_Ve"]=c(1,rep(-1/5,4))

LBrain[c("OT_Ve","CE_Ve","DI_Ve","OB_Ve","TE_Ve"),"OT-avg_Ve"]=c(1,rep(-1/5,4))

LBrain[c("TE_Ve","CE_Ve","DI_Ve","OB_Ve","OT_Ve"),"TE-avg_Ve"]=c(1,rep(-1/5,4))

```

#In the screening stage, a global Ftest across all contrasts is performed for each gene to see whether any of the contrasts is significant.This results in a vector pScreen with one pvalue per gene.WARNING: these pvalues are not corrected for multiple testing and can thus not be used!!

```{r}

alpha=0.05

screenTest = glmQLFTest(fit_2way, contrast=LBrain)

pScreen <- screenTest$table$PValue

names(pScreen) <- rownames(screenTest$table)

```

#In the confirmation stage, the Ftest is performed for each contrast. Pvalues generated here are then corrected with the holm method. This tests for genes with logFC > O

```{r}

confirmationResultsHyp1 <- sapply(1:ncol(LBrain),function(i) glmQLFTest(fit_2way, contrast = LBrain[,i]), simplify=FALSE) #calculates Ftest for each contrast

confirmationPList <- lapply(confirmationResultsHyp1, function(x) x$table$PValue)#takes the p-values from all genes for each contrast and puts them in a list

confirmationP <- as.matrix(Reduce(f=cbind,confirmationPList))

rownames(confirmationP) <- rownames(confirmationResultsHyp1[[1]]$table)

colnames(confirmationP) <- colnames(LBrain)

stageRObj <- stageR(pScreen=pScreen, pConfirmation=confirmationP) #constructs an object

stageRAdj <- stageWiseAdjustment(object=stageRObj, method="holm", alpha=0.05) # adjusts the p-values using FWER correction; here we choose the holm method

resBrain <- getResults(stageRAdj)

resBrain

SignifGenesBrain = colSums(resBrain) #number of DE genes in every contrast.

SignifGenesBrain

adjusted_p = getAdjustedPValues(stageRAdj) #p-values to report in manuscript.pAdjScreen = pvalue of the global test, other columns are then the posthoc tests

```

#To find unique DE genes for each brainpart and shared DE between brainparts (global), we first remove genes for which the global test was not significant.Then, we extract the names of the genes that were significant for each contrast.

```{r}

resbrain_df = as.data.frame(resBrain) #resBrain is a matrix, to be able to subset, change it to a dataframe

resbrain_df2 = resbrain_df

resbrain_df2$gene = rownames(resbrain_df2)

OnlySignGenes = resbrain_df[resbrain_df$padjScreen == 1,] #removes rows for which global test was non significant

dim(OnlySignGenes) # 8748 significant after correction; still includes genes for which all posthoc tests were 0

setEPS()

postscript(paste("SignGenesBrainpart.eps", sep = ""))

barplot(as.matrix(OnlySignGenes), ylab="Number of significant genes", ylim = c(0,9000), names.arg = colnames(OnlySignGenes), cex.names = 0.8, las=2)

dev.off()

#counting unique values:

sum(OnlySignGenes$`BS-avg_Na`== 1 & OnlySignGenes$`CE-avg_Na` == 0 & OnlySignGenes$`DI-avg_Na` == 0 & OnlySignGenes$`OB-avg_Na` == 0 & OnlySignGenes$`OT-avg_Na` == 0 & OnlySignGenes$`TE-avg_Na`== 0) # 340

sum(OnlySignGenes$`BS-avg_Na`== 0 & OnlySignGenes$`CE-avg_Na` == 1 & OnlySignGenes$`DI-avg_Na` == 0 & OnlySignGenes$`OB-avg_Na` == 0 & OnlySignGenes$`OT-avg_Na` == 0 & OnlySignGenes$`TE-avg_Na`== 0) #806

sum(OnlySignGenes$`BS-avg_Na`== 0 & OnlySignGenes$`CE-avg_Na` == 0 & OnlySignGenes$`DI-avg_Na` == 1 & OnlySignGenes$`OB-avg_Na` == 0 & OnlySignGenes$`OT-avg_Na` == 0 & OnlySignGenes$`TE-avg_Na`== 0) #152

sum(OnlySignGenes$`BS-avg_Na`== 0 & OnlySignGenes$`CE-avg_Na` == 0 & OnlySignGenes$`DI-avg_Na` == 0 & OnlySignGenes$`OB-avg_Na` == 1 & OnlySignGenes$`OT-avg_Na` == 0 & OnlySignGenes$`TE-avg_Na`== 0) #236

sum(OnlySignGenes$`BS-avg_Na`== 0 & OnlySignGenes$`CE-avg_Na` == 0 & OnlySignGenes$`DI-avg_Na` == 0 & OnlySignGenes$`OB-avg_Na` == 0 & OnlySignGenes$`OT-avg_Na` == 1 & OnlySignGenes$`TE-avg_Na`== 0) #324

sum(OnlySignGenes$`BS-avg_Na`== 0 & OnlySignGenes$`CE-avg_Na` == 0 & OnlySignGenes$`DI-avg_Na` == 0 & OnlySignGenes$`OB-avg_Na` == 0 & OnlySignGenes$`OT-avg_Na` == 0 & OnlySignGenes$`TE-avg_Na`== 1) #410

sum(OnlySignGenes$`BS-avg_Ve`== 1 & OnlySignGenes$`CE-avg_Ve` == 0 & OnlySignGenes$`DI-avg_Ve` == 0 & OnlySignGenes$`OB-avg_Ve` == 0 & OnlySignGenes$`OT-avg_Ve` == 0 & OnlySignGenes$`TE-avg_Ve`== 0) # 291

sum(OnlySignGenes$`BS-avg_Ve`== 0 & OnlySignGenes$`CE-avg_Ve` == 1 & OnlySignGenes$`DI-avg_Ve` == 0 & OnlySignGenes$`OB-avg_Ve` == 0 & OnlySignGenes$`OT-avg_Ve` == 0 & OnlySignGenes$`TE-avg_Ve`== 0) #1004

sum(OnlySignGenes$`BS-avg_Ve`== 0 & OnlySignGenes$`CE-avg_Ve` == 0 & OnlySignGenes$`DI-avg_Ve` == 1 & OnlySignGenes$`OB-avg_Ve` == 0 & OnlySignGenes$`OT-avg_Ve` == 0 & OnlySignGenes$`TE-avg_Ve`== 0) #197

sum(OnlySignGenes$`BS-avg_Ve`== 0 & OnlySignGenes$`CE-avg_Ve` == 0 & OnlySignGenes$`DI-avg_Ve` == 0 & OnlySignGenes$`OB-avg_Ve` == 1 & OnlySignGenes$`OT-avg_Ve` == 0 & OnlySignGenes$`TE-avg_Ve`== 0) #349

sum(OnlySignGenes$`BS-avg_Ve`== 0 & OnlySignGenes$`CE-avg_Ve` == 0 & OnlySignGenes$`DI-avg_Ve` == 0 & OnlySignGenes$`OB-avg_Ve` == 0 & OnlySignGenes$`OT-avg_Ve` == 1 & OnlySignGenes$`TE-avg_Ve`== 0) #183

sum(OnlySignGenes$`BS-avg_Ve`== 0 & OnlySignGenes$`CE-avg_Ve` == 0 & OnlySignGenes$`DI-avg_Ve` == 0 & OnlySignGenes$`OB-avg_Ve` == 0 & OnlySignGenes$`OT-avg_Ve` == 0 & OnlySignGenes$`TE-avg_Ve`== 1) #370

```

# to make venn diagrams of shared DE genes between the species for each brainpart

```{r}

#to get names of all DE genes unique for each brainpart, and for all brainparts in nasuta:

BS_avgNa = c(rownames (subset (OnlySignGenes, OnlySignGenes$`BS-avg_Na`== 1 & OnlySignGenes$`CE-avg_Na` == 0 & OnlySignGenes$`DI-avg_Na` == 0 & OnlySignGenes$`OB-avg_Na` == 0 & OnlySignGenes$`OT-avg_Na` == 0 & OnlySignGenes$`TE-avg_Na`== 0)))

CE_avgNa = c(rownames (subset (OnlySignGenes, OnlySignGenes$`BS-avg_Na`== 0 & OnlySignGenes$`CE-avg_Na` == 1 & OnlySignGenes$`DI-avg_Na` == 0 & OnlySignGenes$`OB-avg_Na` == 0 & OnlySignGenes$`OT-avg_Na` == 0 & OnlySignGenes$`TE-avg_Na`== 0)))

DI_avgNa = c(rownames (subset (OnlySignGenes, OnlySignGenes$`BS-avg_Na`== 0 & OnlySignGenes$`CE-avg_Na` == 0 & OnlySignGenes$`DI-avg_Na` == 1 & OnlySignGenes$`OB-avg_Na` == 0 & OnlySignGenes$`OT-avg_Na` == 0 & OnlySignGenes$`TE-avg_Na`== 0)))

OB_avgNa = c(rownames (subset (OnlySignGenes, OnlySignGenes$`BS-avg_Na`== 0 & OnlySignGenes$`CE-avg_Na` == 0 & OnlySignGenes$`DI-avg_Na` == 0 & OnlySignGenes$`OB-avg_Na` == 1 & OnlySignGenes$`OT-avg_Na` == 0 & OnlySignGenes$`TE-avg_Na`== 0)))

OT_avgNa = c(rownames (subset (OnlySignGenes, OnlySignGenes$`BS-avg_Na`== 0 & OnlySignGenes$`CE-avg_Na` == 0 & OnlySignGenes$`DI-avg_Na` == 0 & OnlySignGenes$`OB-avg_Na` == 0 & OnlySignGenes$`OT-avg_Na` == 1 & OnlySignGenes$`TE-avg_Na`== 0)))

TE_avgNa = c(rownames (subset (OnlySignGenes, OnlySignGenes$`BS-avg_Na`== 0 & OnlySignGenes$`CE-avg_Na` == 0 & OnlySignGenes$`DI-avg_Na` == 0 & OnlySignGenes$`OB-avg_Na` == 0 & OnlySignGenes$`OT-avg_Na` == 0 & OnlySignGenes$`TE-avg_Na`== 1)))

allSignGenes_Na = c(rownames (subset (OnlySignGenes, OnlySignGenes$`BS-avg_Na`== 1 | OnlySignGenes$`CE-avg_Na` == 1 | OnlySignGenes$`DI-avg_Na` == 1 | OnlySignGenes$`OB-avg_Na` == 1 | OnlySignGenes$`OT-avg_Na` == 1 | OnlySignGenes$`TE-avg_Na`== 1)))

#to get names of all DE genes unique for each brainpart, and for all brainparts in ventralis:

BS_avgVe = c(rownames (subset (OnlySignGenes, OnlySignGenes$`BS-avg_Ve`== 1 & OnlySignGenes$`CE-avg_Ve` == 0 & OnlySignGenes$`DI-avg_Ve` == 0 & OnlySignGenes$`OB-avg_Ve` == 0 & OnlySignGenes$`OT-avg_Ve` == 0 & OnlySignGenes$`TE-avg_Ve`== 0)))

CE_avgVe = c(rownames (subset (OnlySignGenes, OnlySignGenes$`BS-avg_Ve`== 0 & OnlySignGenes$`CE-avg_Ve` == 1 & OnlySignGenes$`DI-avg_Ve` == 0 & OnlySignGenes$`OB-avg_Ve` == 0 & OnlySignGenes$`OT-avg_Ve` == 0 & OnlySignGenes$`TE-avg_Ve`== 0)))

DI_avgVe = c(rownames (subset (OnlySignGenes, OnlySignGenes$`BS-avg_Ve`== 0 & OnlySignGenes$`CE-avg_Ve` == 0 & OnlySignGenes$`DI-avg_Ve` == 1 & OnlySignGenes$`OB-avg_Ve` == 0 & OnlySignGenes$`OT-avg_Ve` == 0 & OnlySignGenes$`TE-avg_Ve`== 0)))

OB_avgVe = c(rownames (subset (OnlySignGenes, OnlySignGenes$`BS-avg_Ve`== 0 & OnlySignGenes$`CE-avg_Ve` == 0 & OnlySignGenes$`DI-avg_Ve` == 0 & OnlySignGenes$`OB-avg_Ve` == 1 & OnlySignGenes$`OT-avg_Ve` == 0 & OnlySignGenes$`TE-avg_Ve`== 0)))

OT_avgVe = c(rownames (subset (OnlySignGenes, OnlySignGenes$`BS-avg_Ve`== 0 & OnlySignGenes$`CE-avg_Ve` == 0 & OnlySignGenes$`DI-avg_Ve` == 0 & OnlySignGenes$`OB-avg_Ve` == 0 & OnlySignGenes$`OT-avg_Ve` == 1 & OnlySignGenes$`TE-avg_Ve`== 0)))

TE_avgVe = c(rownames (subset (OnlySignGenes, OnlySignGenes$`BS-avg_Ve`== 0 & OnlySignGenes$`CE-avg_Ve` == 0 & OnlySignGenes$`DI-avg_Ve` == 0 & OnlySignGenes$`OB-avg_Ve` == 0 & OnlySignGenes$`OT-avg_Ve` == 0 & OnlySignGenes$`TE-avg_Ve`== 1)))

allSignGenes_Ve = c(rownames (subset (OnlySignGenes, OnlySignGenes$`BS-avg_Ve`== 1 | OnlySignGenes$`CE-avg_Ve` == 1 | OnlySignGenes$`DI-avg_Ve` == 1 | OnlySignGenes$`OB-avg_Ve` == 1 | OnlySignGenes$`OT-avg_Ve` == 1 | OnlySignGenes$`TE-avg_Ve`== 1)))

NamesOnlySignGenes= names(which(resBrain[,"padjScreen"]==1))

NamesOnlySignGenes_BS_Na= names(which(resBrain[,"BS-avg_Na"]==1))

NamesOnlySignGenes_BS_Ve= names(which(resBrain[,"BS-avg_Ve"]==1))

NamesOnlySignGenes_CE_Na= names(which(resBrain[,"CE-avg_Na"]==1))

NamesOnlySignGenes_CE_Ve= names(which(resBrain[,"CE-avg_Ve"]==1))

NamesOnlySignGenes_DI_Na= names(which(resBrain[,"DI-avg_Na"]==1))

NamesOnlySignGenes_DI_Ve= names(which(resBrain[,"DI-avg_Ve"]==1))

NamesOnlySignGenes_OB_Na= names(which(resBrain[,"OB-avg_Na"]==1))

NamesOnlySignGenes_OB_Ve= names(which(resBrain[,"OB-avg_Ve"]==1))

NamesOnlySignGenes_OT_Na= names(which(resBrain[,"OT-avg_Na"]==1))

NamesOnlySignGenes_OT_Ve= names(which(resBrain[,"OT-avg_Ve"]==1))

NamesOnlySignGenes_TE_Na= names(which(resBrain[,"TE-avg_Na"]==1))

NamesOnlySignGenes_TE_Ve= names(which(resBrain[,"TE-avg_Ve"]==1))

write.table (NamesOnlySignGenes_TE_Ve, "NamesOnlySignGenes_TE_Ve.txt", col.names = FALSE, row.names = FALSE, quote = FALSE)

write.table (NamesOnlySignGenes_TE_Na, "NamesOnlySignGenes_TE_Na.txt", col.names = FALSE, row.names = FALSE, quote = FALSE)

write.table (NamesOnlySignGenes_CE_Ve, "NamesOnlySignGenes_CE_Ve.txt", col.names = FALSE, row.names = FALSE, quote = FALSE)

write.table (NamesOnlySignGenes_CE_Na, "NamesOnlySignGenes_CE_Na.txt", col.names = FALSE, row.names = FALSE, quote = FALSE)

write.table (NamesOnlySignGenes_OB_Ve, "NamesOnlySignGenes_OB_Ve.txt", col.names = FALSE, row.names = FALSE, quote = FALSE)

write.table (NamesOnlySignGenes_OB_Na, "NamesOnlySignGenes_OB_Na.txt", col.names = FALSE, row.names = FALSE, quote = FALSE)

write.table (NamesOnlySignGenes_OT_Ve, "NamesOnlySignGenes_OT_Ve.txt", col.names = FALSE, row.names = FALSE, quote = FALSE)

write.table (NamesOnlySignGenes_OT_Na, "NamesOnlySignGenes_OT_Na.txt", col.names = FALSE, row.names = FALSE, quote = FALSE)

write.table (NamesOnlySignGenes_BS_Ve, "NamesOnlySignGenes_BS_Ve.txt", col.names = FALSE, row.names = FALSE, quote = FALSE)

write.table (NamesOnlySignGenes_BS_Na, "NamesOnlySignGenes_BS_Na.txt", col.names = FALSE, row.names = FALSE, quote = FALSE)

write.table (NamesOnlySignGenes_DI_Ve, "NamesOnlySignGenes_DI_Ve.txt", col.names = FALSE, row.names = FALSE, quote = FALSE)

write.table (NamesOnlySignGenes_DI_Na, "NamesOnlySignGenes_DI_Na.txt", col.names = FALSE, row.names = FALSE, quote = FALSE)

pdf("venn_BS.pdf")

venn_BS <- venn.diagram(list(NamesOnlySignGenes_BS_Na, NamesOnlySignGenes_BS_Ve), NULL, alpha=c(0.5,0.5), cex = 2, cat.fontface=1, category.names=c("BS_Na", "BS_Ve"), cat.pos = c(0,0), cat.cex = c(2,2))

grid.draw(venn_BS)

dev.off()

pdf("venn_CE.pdf")

venn_CE <- venn.diagram(list(NamesOnlySignGenes_CE_Na, NamesOnlySignGenes_CE_Ve), NULL, alpha=c(0.5,0.5), cex = 2, cat.fontface=1, category.names=c("CE_Na", "CE_Ve"),cat.pos = c(0,0), cat.cex = c(2,2), inverted = TRUE)

grid.draw(venn_CE)

dev.off()

pdf("venn_TE.pdf")

venn_TE <- venn.diagram(list(NamesOnlySignGenes_TE_Na, NamesOnlySignGenes_TE_Ve), NULL, alpha=c(0.5,0.5), cex = 2, cat.fontface=1, category.names=c("TE_Na", "TE_Ve"), cat.pos = c(0,0), cat.cex = c(2,2))

grid.draw(venn_TE)

dev.off()

pdf("venn_OT.pdf")

venn_OT <- venn.diagram(list(NamesOnlySignGenes_OT_Na, NamesOnlySignGenes_OT_Ve), NULL, alpha=c(0.5,0.5), cex = 2, cat.fontface=1, category.names=c("OT_Na", "OT_Ve"), cat.pos = c(0,0), cat.cex = c(2,2))

grid.draw(venn_OT)

dev.off()

pdf("venn_OB.pdf")

venn_OB <- venn.diagram(list(NamesOnlySignGenes_OB_Na, NamesOnlySignGenes_OB_Ve), NULL, alpha=c(0.5,0.5), cex = 2, cat.fontface=1, category.names=c("OB_Na", "OB_Ve"), cat.pos = c(0,0), cat.cex = c(2,2), inverted = TRUE)

grid.draw(venn_OB)

dev.off()

pdf("venn_DI.pdf")

venn_DI <- venn.diagram(list(NamesOnlySignGenes_DI_Na, NamesOnlySignGenes_DI_Ve), NULL, alpha=c(0.5,0.5), cex = 2, cat.fontface=1, category.names=c("DI_Na", "DI_Ve"), cat.pos = c(0,0), cat.cex = c(2,2), inverted = TRUE)

grid.draw(venn_DI)

dev.off()

```

#to get fold changes for the significant gene(s); adjusted P values from stage wise testing are added and only the significant genes are kept;

```{r}

##BSNa

Hyp1SignGenesBSNa=names(which(resBrain[,"BS-avg_Na"]==1))

logFC_BSavgNa = confirmationResultsHyp1[[1]]$table[Hyp1SignGenesBSNa,] #adjust column to select correct contrast

BSNa_adjustedP = subset(adjusted_p, rownames(adjusted_p)%in%Hyp1SignGenesBSNa)

logFC_BSavgNa_adjP = merge(logFC_BSavgNa,BSNa_adjustedP, by = 0, all = TRUE)

logFC_BSavgNa_adjP = logFC_BSavgNa_adjP[, c(1,2,7)] #adjust column to select correct contrast

colnames(logFC_BSavgNa_adjP)[3] = "adjusted Pvalue"

##CENa

Hyp1SignGenesCENa=names(which(resBrain[,"CE-avg_Na"]==1))

logFC_CEavgNa = confirmationResultsHyp1[[3]]$table[Hyp1SignGenesCENa,]

CENa_adjustedP = subset(adjusted_p, rownames(adjusted_p)%in%Hyp1SignGenesCENa)

logFC_CEavgNa_adjP = merge(logFC_CEavgNa,CENa_adjustedP, by = 0, all = TRUE)

logFC_CEavgNa_adjP = logFC_CEavgNa_adjP[, c(1,2,9)]

colnames(logFC_CEavgNa_adjP)[3] = "adjusted Pvalue"

##DINa

Hyp1SignGenesDINa=names(which(resBrain[,"DI-avg_Na"]==1))

logFC_DIavgNa = confirmationResultsHyp1[[5]]$table[Hyp1SignGenesDINa,]

DINa_adjustedP = subset(adjusted_p, rownames(adjusted_p)%in%Hyp1SignGenesDINa)

logFC_DIavgNa_adjP = merge(logFC_DIavgNa,DINa_adjustedP, by = 0, all = TRUE)

logFC_DIavgNa_adjP = logFC_DIavgNa_adjP[, c(1,2,11)]

colnames(logFC_DIavgNa_adjP)[3] = "adjusted Pvalue"

##OBNa

Hyp1SignGenesOBNa=names(which(resBrain[,"OB-avg_Na"]==1))

logFC_OBavgNa = confirmationResultsHyp1[[7]]$table[Hyp1SignGenesOBNa,]

OBNa_adjustedP = subset(adjusted_p, rownames(adjusted_p)%in%Hyp1SignGenesOBNa)

logFC_OBavgNa_adjP = merge(logFC_OBavgNa,OBNa_adjustedP, by = 0, all = TRUE)

logFC_OBavgNa_adjP = logFC_OBavgNa_adjP[, c(1,2,13)]

colnames(logFC_OBavgNa_adjP)[3] = "adjusted Pvalue"

##OTNa

Hyp1SignGenesOTNa=names(which(resBrain[,"OT-avg_Na"]==1))

logFC_OTavgNa = confirmationResultsHyp1[[9]]$table[Hyp1SignGenesOTNa,]

OTNa_adjustedP = subset(adjusted_p, rownames(adjusted_p)%in%Hyp1SignGenesOTNa)

logFC_OTavgNa_adjP = merge(logFC_OTavgNa,OTNa_adjustedP, by = 0, all = TRUE)

logFC_OTavgNa_adjP = logFC_OTavgNa_adjP[, c(1,2,15)]

colnames(logFC_OTavgNa_adjP)[3] = "adjusted Pvalue"

##TENa

Hyp1SignGenesTENa=names(which(resBrain[,"TE-avg_Na"]==1))

logFC_TEavgNa = confirmationResultsHyp1[[11]]$table[Hyp1SignGenesTENa,]

TENa_adjustedP = subset(adjusted_p, rownames(adjusted_p)%in%Hyp1SignGenesTENa)

logFC_TEavgNa_adjP = merge(logFC_TEavgNa,TENa_adjustedP, by = 0, all = TRUE)

logFC_TEavgNa_adjP = logFC_TEavgNa_adjP[, c(1,2,17)]

colnames(logFC_TEavgNa_adjP)[3] = "adjusted Pvalue"

##BSVe

Hyp1SignGenesBSVe=names(which(resBrain[,"BS-avg_Ve"]==1))

logFC_BSavgVe = confirmationResultsHyp1[[2]]$table[Hyp1SignGenesBSVe,] #adjust column to select correct contrast

BSVe_adjustedP = subset(adjusted_p, rownames(adjusted_p)%in%Hyp1SignGenesBSVe)

logFC_BSavgVe_adjP = merge(logFC_BSavgVe,BSVe_adjustedP, by = 0, all = TRUE)

logFC_BSavgVe_adjP = logFC_BSavgVe_adjP[, c(1,2,8)] #adjust column to select correct contrast

colnames(logFC_BSavgVe_adjP)[3] = "adjusted Pvalue"

##CEVe

Hyp1SignGenesCEVe=names(which(resBrain[,"CE-avg_Ve"]==1))

logFC_CEavgVe = confirmationResultsHyp1[[4]]$table[Hyp1SignGenesCEVe,]

CEVe_adjustedP = subset(adjusted_p, rownames(adjusted_p)%in%Hyp1SignGenesCEVe)

logFC_CEavgVe_adjP = merge(logFC_CEavgVe,CEVe_adjustedP, by = 0, all = TRUE)

logFC_CEavgVe_adjP = logFC_CEavgVe_adjP[, c(1,2,10)]

colnames(logFC_CEavgVe_adjP)[3] = "adjusted Pvalue"

##DIVe

Hyp1SignGenesDIVe=names(which(resBrain[,"DI-avg_Ve"]==1))

logFC_DIavgVe = confirmationResultsHyp1[[6]]$table[Hyp1SignGenesDIVe,]

DIVe_adjustedP = subset(adjusted_p, rownames(adjusted_p)%in%Hyp1SignGenesDIVe)

logFC_DIavgVe_adjP = merge(logFC_DIavgVe,DIVe_adjustedP, by = 0, all = TRUE)

logFC_DIavgVe_adjP = logFC_DIavgVe_adjP[, c(1,2,12)]

colnames(logFC_DIavgVe_adjP)[3] = "adjusted Pvalue"

##OBVe

Hyp1SignGenesOBVe=names(which(resBrain[,"OB-avg_Ve"]==1))

logFC_OBavgVe = confirmationResultsHyp1[[8]]$table[Hyp1SignGenesOBVe,]

OBVe_adjustedP = subset(adjusted_p, rownames(adjusted_p)%in%Hyp1SignGenesOBVe)

logFC_OBavgVe_adjP = merge(logFC_OBavgVe,OBVe_adjustedP, by = 0, all = TRUE)

logFC_OBavgVe_adjP = logFC_OBavgVe_adjP[, c(1,2,14)]

colnames(logFC_OBavgVe_adjP)[3] = "adjusted Pvalue"

##OTVe

Hyp1SignGenesOTVe=names(which(resBrain[,"OT-avg_Ve"]==1))

logFC_OTavgVe = confirmationResultsHyp1[[10]]$table[Hyp1SignGenesOTVe,]

OTVe_adjustedP = subset(adjusted_p, rownames(adjusted_p)%in%Hyp1SignGenesOTVe)

logFC_OTavgVe_adjP = merge(logFC_OTavgVe,OTVe_adjustedP, by = 0, all = TRUE)

logFC_OTavgVe_adjP = logFC_OTavgVe_adjP[, c(1,2,16)]

colnames(logFC_OTavgVe_adjP)[3] = "adjusted Pvalue"

##TEVe

Hyp1SignGenesTEVe=names(which(resBrain[,"TE-avg_Ve"]==1))

logFC_TEavgVe = confirmationResultsHyp1[[12]]$table[Hyp1SignGenesTEVe,]

TEVe_adjustedP = subset(adjusted_p, rownames(adjusted_p)%in%Hyp1SignGenesTEVe)

logFC_TEavgVe_adjP = merge(logFC_TEavgVe,TEVe_adjustedP, by = 0, all = TRUE)

logFC_TEavgVe_adjP = logFC_TEavgVe_adjP[, c(1,2,18)]

colnames(logFC_TEavgVe_adjP)[3] = "adjusted Pvalue"

```

# to find genes that have logFC significantly > 2 or significantly < -2 => for this, we have to test for DE relative to a threshold in the confirmation stage; this is done with the glmtreat function

```{r}

confirmationResultsFC2 <- sapply(1:ncol(LBrain),function(i) glmTreat(fit_2way, contrast = LBrain[,i], lfc=2),simplify = FALSE)

confirmationPListFC2 <- lapply(confirmationResultsFC2, function(x) x$table$PValue)#takes the p-values from all genes for each contrast and puts them in a list

confirmationPFC2 <- as.matrix(Reduce(f=cbind,confirmationPListFC2))

rownames(confirmationPFC2) <- rownames(fit_2way)

colnames(confirmationPFC2) <- colnames(LBrain)

stageRObjFC2 <- stageR(pScreen=pScreen, pConfirmation=confirmationPFC2) #constructs an object

stageRAdjFC2 <- stageWiseAdjustment(object=stageRObjFC2, method="holm", alpha=0.05) # adjusts the p-values using FWER correction; here we choose the holm method

resBrainFC2 <- getResults(stageRAdjFC2)

SignifGenesBrainFC2 = colSums(resBrainFC2) #number of DE genes in every contrast.

SignifGenesBrainFC2

adjusted_pFC2 = getAdjustedPValues(stageRAdjFC2) #p-values to report in manuscript.pAdjScreen = pvalue of the global test, other columns are then the posthoc tests

resbrainFC2_df = as.data.frame(resBrainFC2) #resBrain is a matrix, to be able to subset, change it to a dataframe

OnlySignGenesFC2 = resbrainFC2_df[resbrainFC2_df$padjScreen == 1,] #removes rows for which global test was non significant

##to get fold changes of the DE genes with logFC >2

##BSNa

Hyp1SignGenesBSNaFC2=names(which(resBrainFC2[,"BS-avg_Na"]==1))

logFC2_BSavgNa = confirmationResultsFC2[[1]]$table[Hyp1SignGenesBSNaFC2,] #adjust column to select correct contrast

BSNa_adjustedPFC2 = subset(adjusted_pFC2, rownames(adjusted_pFC2)%in%Hyp1SignGenesBSNaFC2)

logFC2_BSavgNa_adjP = merge(logFC2_BSavgNa,BSNa_adjustedPFC2, by = 0, all = TRUE)

logFC2_BSavgNa_adjP = logFC2_BSavgNa_adjP[, c(1,2,7)] #adjust column to select correct contrast

colnames(logFC2_BSavgNa_adjP)[3] = "adjusted Pvalue"

##CENa

Hyp1SignGenesCENaFC2=names(which(resBrainFC2[,"CE-avg_Na"]==1))

logFC2_CEavgNa = confirmationResultsFC2[[3]]$table[Hyp1SignGenesCENaFC2,] #adjust column to select correct contrast

CENa_adjustedPFC2 = subset(adjusted_pFC2, rownames(adjusted_pFC2)%in%Hyp1SignGenesCENaFC2)

logFC2_CEavgNa_adjP = merge(logFC2_CEavgNa,CENa_adjustedPFC2, by = 0, all = TRUE)

logFC2_CEavgNa_adjP = logFC2_CEavgNa_adjP[, c(1,2,9)] #adjust column to select correct contrast

colnames(logFC2_CEavgNa_adjP)[3] = "adjusted Pvalue"

##DINa

Hyp1SignGenesDINaFC2=names(which(resBrainFC2[,"DI-avg_Na"]==1))

logFC2_DIavgNa = confirmationResultsFC2[[5]]$table[Hyp1SignGenesDINaFC2,] #adjust column to select correct contrast

DINa_adjustedPFC2 = subset(adjusted_pFC2, rownames(adjusted_pFC2)%in%Hyp1SignGenesDINaFC2)

logFC2_DIavgNa_adjP = merge(logFC2_DIavgNa,DINa_adjustedPFC2, by = 0, all = TRUE)

logFC2_DIavgNa_adjP = logFC2_DIavgNa_adjP[, c(1,2,11)] #adjust column to select correct contrast

colnames(logFC2_DIavgNa_adjP)[3] = "adjusted Pvalue"

##OBNa

Hyp1SignGenesOBNaFC2=names(which(resBrainFC2[,"OB-avg_Na"]==1))

logFC2_OBavgNa = confirmationResultsFC2[[7]]$table[Hyp1SignGenesOBNaFC2,] #adjust column to select correct contrast

OBNa_adjustedPFC2 = subset(adjusted_pFC2, rownames(adjusted_pFC2)%in%Hyp1SignGenesOBNaFC2)

logFC2_OBavgNa_adjP = merge(logFC2_OBavgNa,OBNa_adjustedPFC2, by = 0, all = TRUE)

logFC2_OBavgNa_adjP = logFC2_OBavgNa_adjP[, c(1,2,13)] #adjust column to select correct contrast

colnames(logFC2_OBavgNa_adjP)[3] = "adjusted Pvalue"

##OTNa

Hyp1SignGenesOTNaFC2=names(which(resBrainFC2[,"OT-avg_Na"]==1))

logFC2_OTavgNa = confirmationResultsFC2[[9]]$table[Hyp1SignGenesOTNaFC2,] #adjust column to select correct contrast

OTNa_adjustedPFC2 = subset(adjusted_pFC2, rownames(adjusted_pFC2)%in%Hyp1SignGenesOTNaFC2)

logFC2_OTavgNa_adjP = merge(logFC2_OTavgNa,OTNa_adjustedPFC2, by = 0, all = TRUE)

logFC2_OTavgNa_adjP = logFC2_OTavgNa_adjP[, c(1,2,15)] #adjust column to select correct contrast

colnames(logFC2_OTavgNa_adjP)[3] = "adjusted Pvalue"

##TENa

Hyp1SignGenesTENaFC2=names(which(resBrainFC2[,"TE-avg_Na"]==1))

logFC2_TEavgNa = confirmationResultsFC2[[11]]$table[Hyp1SignGenesTENaFC2,] #adjust column to select correct contrast

TENa_adjustedPFC2 = subset(adjusted_pFC2, rownames(adjusted_pFC2)%in%Hyp1SignGenesTENaFC2)

logFC2_TEavgNa_adjP = merge(logFC2_TEavgNa,TENa_adjustedPFC2, by = 0, all = TRUE)

logFC2_TEavgNa_adjP = logFC2_TEavgNa_adjP[, c(1,2,17)] #adjust column to select correct contrast

colnames(logFC2_TEavgNa_adjP)[3] = "adjusted Pvalue"

##BSVe

Hyp1SignGenesBSVeFC2=names(which(resBrainFC2[,"BS-avg_Ve"]==1))

logFC2_BSavgVe = confirmationResultsFC2[[2]]$table[Hyp1SignGenesBSVeFC2,] #adjust column to select correct contrast

BSVe_adjustedPFC2 = subset(adjusted_pFC2, rownames(adjusted_pFC2)%in%Hyp1SignGenesBSVeFC2)

logFC2_BSavgVe_adjP = merge(logFC2_BSavgVe,BSVe_adjustedPFC2, by = 0, all = TRUE)

logFC2_BSavgVe_adjP = logFC2_BSavgVe_adjP[, c(1,2,8)] #adjust column to select correct contrast

colnames(logFC2_BSavgVe_adjP)[3] = "adjusted Pvalue"

##CEVe

Hyp1SignGenesCEVeFC2=names(which(resBrainFC2[,"CE-avg_Ve"]==1))

logFC2_CEavgVe = confirmationResultsFC2[[4]]$table[Hyp1SignGenesCEVeFC2,] #adjust column to select correct contrast

CEVe_adjustedPFC2 = subset(adjusted_pFC2, rownames(adjusted_pFC2)%in%Hyp1SignGenesCEVeFC2)

logFC2_CEavgVe_adjP = merge(logFC2_CEavgVe,CEVe_adjustedPFC2, by = 0, all = TRUE)

logFC2_CEavgVe_adjP = logFC2_CEavgVe_adjP[, c(1,2,10)] #adjust column to select correct contrast

colnames(logFC2_CEavgVe_adjP)[3] = "adjusted Pvalue"

##DIVe

Hyp1SignGenesDIVeFC2=names(which(resBrainFC2[,"DI-avg_Ve"]==1))

logFC2_DIavgVe = confirmationResultsFC2[[6]]$table[Hyp1SignGenesDIVeFC2,] #adjust column to select correct contrast

DIVe_adjustedPFC2 = subset(adjusted_pFC2, rownames(adjusted_pFC2)%in%Hyp1SignGenesDIVeFC2)

logFC2_DIavgVe_adjP = merge(logFC2_DIavgVe,DIVe_adjustedPFC2, by = 0, all = TRUE)

logFC2_DIavgVe_adjP = logFC2_DIavgVe_adjP[, c(1,2,12)] #adjust column to select correct contrast

colnames(logFC2_DIavgVe_adjP)[3] = "adjusted Pvalue"

##OBVe

Hyp1SignGenesOBVeFC2=names(which(resBrainFC2[,"OB-avg_Ve"]==1))

logFC2_OBavgVe = confirmationResultsFC2[[8]]$table[Hyp1SignGenesOBVeFC2,] #adjust column to select correct contrast

OBVe_adjustedPFC2 = subset(adjusted_pFC2, rownames(adjusted_pFC2)%in%Hyp1SignGenesOBVeFC2)

logFC2_OBavgVe_adjP = merge(logFC2_OBavgVe,OBVe_adjustedPFC2, by = 0, all = TRUE)

logFC2_OBavgVe_adjP = logFC2_OBavgVe_adjP[, c(1,2,14)] #adjust column to select correct contrast

colnames(logFC2_OBavgVe_adjP)[3] = "adjusted Pvalue"

##OTVe

Hyp1SignGenesOTVeFC2=names(which(resBrainFC2[,"OT-avg_Ve"]==1))

logFC2_OTavgVe = confirmationResultsFC2[[10]]$table[Hyp1SignGenesOTVeFC2,] #adjust column to select correct contrast

OTVe_adjustedPFC2 = subset(adjusted_pFC2, rownames(adjusted_pFC2)%in%Hyp1SignGenesOTVeFC2)

logFC2_OTavgVe_adjP = merge(logFC2_OTavgVe,OTVe_adjustedPFC2, by = 0, all = TRUE)

logFC2_OTavgVe_adjP = logFC2_OTavgVe_adjP[, c(1,2,16)] #adjust column to select correct contrast

colnames(logFC2_OTavgVe_adjP)[3] = "adjusted Pvalue"

##TEVe

Hyp1SignGenesTEVeFC2=names(which(resBrainFC2[,"TE-avg_Ve"]==1))

logFC2_TEavgVe = confirmationResultsFC2[[12]]$table[Hyp1SignGenesTEVeFC2,] #adjust column to select correct contrast

TEVe_adjustedPFC2 = subset(adjusted_pFC2, rownames(adjusted_pFC2)%in%Hyp1SignGenesTEVeFC2)

logFC2_TEavgVe_adjP = merge(logFC2_TEavgVe,TEVe_adjustedPFC2, by = 0, all = TRUE)

logFC2_TEavgVe_adjP = logFC2_TEavgVe_adjP[, c(1,2,18)] #adjust column to select correct contrast

colnames(logFC2_TEavgVe_adjP)[3] = "adjusted Pvalue"

##merge files

logFC2_BS = merge(logFC2_BSavgNa_adjP, logFC2_BSavgVe_adjP, by.x = "Row.names", by.y = "Row.names")

logFC2_CE = merge(logFC2_CEavgNa_adjP, logFC2_CEavgVe_adjP, by.x = "Row.names", by.y = "Row.names")

logFC2_DI = merge(logFC2_DIavgNa_adjP, logFC2_DIavgVe_adjP, by.x = "Row.names", by.y = "Row.names")

logFC2_OB = merge(logFC2_OBavgNa_adjP, logFC2_OBavgVe_adjP, by.x = "Row.names", by.y = "Row.names")

logFC2_OT = merge(logFC2_OTavgNa_adjP, logFC2_OTavgVe_adjP, by.x = "Row.names", by.y = "Row.names")

logFC2_TE = merge(logFC2_TEavgNa_adjP, logFC2_TEavgVe_adjP, by.x = "Row.names", by.y = "Row.names")

```

```{r}

## names of genes with logFC >2 and only DE in one brain part in one species

BS_avgNaFC2 = c(rownames (subset (OnlySignGenesFC2, OnlySignGenesFC2$`BS-avg_Na`== 1 & OnlySignGenesFC2$`CE-avg_Na` == 0 & OnlySignGenesFC2$`DI-avg_Na` == 0 & OnlySignGenesFC2$`OB-avg_Na` == 0 & OnlySignGenesFC2$`OT-avg_Na` == 0 & OnlySignGenesFC2$`TE-avg_Na`== 0)))

CE_avgNaFC2 = c(rownames (subset (OnlySignGenesFC2, OnlySignGenesFC2$`BS-avg_Na`== 0 & OnlySignGenesFC2$`CE-avg_Na` == 1 & OnlySignGenesFC2$`DI-avg_Na` == 0 & OnlySignGenesFC2$`OB-avg_Na` == 0 & OnlySignGenesFC2$`OT-avg_Na` == 0 & OnlySignGenesFC2$`TE-avg_Na`== 0)))

DI_avgNaFC2 = c(rownames (subset (OnlySignGenesFC2, OnlySignGenesFC2$`BS-avg_Na`== 0 & OnlySignGenesFC2$`CE-avg_Na` == 0 & OnlySignGenesFC2$`DI-avg_Na` == 1 & OnlySignGenesFC2$`OB-avg_Na` == 0 & OnlySignGenesFC2$`OT-avg_Na` == 0 & OnlySignGenesFC2$`TE-avg_Na`== 0)))

OB_avgNaFC2 = c(rownames (subset (OnlySignGenesFC2, OnlySignGenesFC2$`BS-avg_Na`== 0 & OnlySignGenesFC2$`CE-avg_Na` == 0 & OnlySignGenesFC2$`DI-avg_Na` == 0 & OnlySignGenesFC2$`OB-avg_Na` == 1 & OnlySignGenesFC2$`OT-avg_Na` == 0 & OnlySignGenesFC2$`TE-avg_Na`== 0)))

OT_avgNaFC2 = c(rownames (subset (OnlySignGenesFC2, OnlySignGenesFC2$`BS-avg_Na`== 0 & OnlySignGenesFC2$`CE-avg_Na` == 0 & OnlySignGenesFC2$`DI-avg_Na` == 0 & OnlySignGenesFC2$`OB-avg_Na` == 0 & OnlySignGenesFC2$`OT-avg_Na` == 1 & OnlySignGenesFC2$`TE-avg_Na`== 0)))

TE_avgNaFC2 = c(rownames (subset (OnlySignGenesFC2, OnlySignGenesFC2$`BS-avg_Na`== 0 & OnlySignGenesFC2$`CE-avg_Na` == 0 & OnlySignGenesFC2$`DI-avg_Na` == 0 & OnlySignGenesFC2$`OB-avg_Na` == 0 & OnlySignGenesFC2$`OT-avg_Na` == 0 & OnlySignGenesFC2$`TE-avg_Na`== 1)))

BS_avgVeFC2 = c(rownames (subset (OnlySignGenesFC2, OnlySignGenesFC2$`BS-avg_Ve`== 1 & OnlySignGenesFC2$`CE-avg_Ve` == 0 & OnlySignGenesFC2$`DI-avg_Ve` == 0 & OnlySignGenesFC2$`OB-avg_Ve` == 0 & OnlySignGenesFC2$`OT-avg_Ve` == 0 & OnlySignGenesFC2$`TE-avg_Ve`== 0)))

CE_avgVeFC2 = c(rownames (subset (OnlySignGenesFC2, OnlySignGenesFC2$`BS-avg_Ve`== 0 & OnlySignGenesFC2$`CE-avg_Ve` == 1 & OnlySignGenesFC2$`DI-avg_Ve` == 0 & OnlySignGenesFC2$`OB-avg_Ve` == 0 & OnlySignGenesFC2$`OT-avg_Ve` == 0 & OnlySignGenesFC2$`TE-avg_Ve`== 0)))

DI_avgVeFC2 = c(rownames (subset (OnlySignGenesFC2, OnlySignGenesFC2$`BS-avg_Ve`== 0 & OnlySignGenesFC2$`CE-avg_Ve` == 0 & OnlySignGenesFC2$`DI-avg_Ve` == 1 & OnlySignGenesFC2$`OB-avg_Ve` == 0 & OnlySignGenesFC2$`OT-avg_Ve` == 0 & OnlySignGenesFC2$`TE-avg_Ve`== 0)))

OB_avgVeFC2 = c(rownames (subset (OnlySignGenesFC2, OnlySignGenesFC2$`BS-avg_Ve`== 0 & OnlySignGenesFC2$`CE-avg_Ve` == 0 & OnlySignGenesFC2$`DI-avg_Ve` == 0 & OnlySignGenesFC2$`OB-avg_Ve` == 1 & OnlySignGenesFC2$`OT-avg_Ve` == 0 & OnlySignGenesFC2$`TE-avg_Ve`== 0)))

OT_avgVeFC2 = c(rownames (subset (OnlySignGenesFC2, OnlySignGenesFC2$`BS-avg_Ve`== 0 & OnlySignGenesFC2$`CE-avg_Ve` == 0 & OnlySignGenesFC2$`DI-avg_Ve` == 0 & OnlySignGenesFC2$`OB-avg_Ve` == 0 & OnlySignGenesFC2$`OT-avg_Ve` == 1 & OnlySignGenesFC2$`TE-avg_Ve`== 0)))

TE_avgVeFC2 = c(rownames (subset (OnlySignGenesFC2, OnlySignGenesFC2$`BS-avg_Ve`== 0 & OnlySignGenesFC2$`CE-avg_Ve` == 0 & OnlySignGenesFC2$`DI-avg_Ve` == 0 & OnlySignGenesFC2$`OB-avg_Ve` == 0 & OnlySignGenesFC2$`OT-avg_Ve` == 0 & OnlySignGenesFC2$`TE-avg_Ve`== 1)))

## III.4.3. Hypothesis 2: There is no differential expression between a brain part and another brain part between species (i.e. the interaction brain part x species)

## III.4.3.1. Screening stage

```{r}

## contrasts between species.

LBetween=matrix(0,nrow=ncol(fit_2way$coefficients),ncol=15)

rownames(LBetween)=colnames(fit_2way$coefficients)

colnames(LBetween)=c("BSvCE_Na-BSvCE_Ve","BSvDI_Na-BSvDI_Ve","BSvOB_Na-BSvOB_Ve","BSvOT_Na-BSvOT_Ve","BSvTE_Na-BSvTE_Ve",

"CEvDI_Na-CEvDI_Ve","CEvOB_Na-CEvOB_Ve","CEvOT_Na-CEvOT_Ve","CEvTE_Na-CEvTE_Ve",

"DIvOB_Na-DIvOB_Ve","DIvOT_Na-DIvOT_Ve","DIvTE_Na-DIvTE_Ve",

"OBvOT_Na-OBvOT_Ve","OBvTE_Na-OBvTE_Ve",

"OTvTE_Na-OTvTE_Ve")

LBetween[11:12,1]=c(-1,1)

LBetween[13:14,2]=c(-1,1)

LBetween[15:16,3]=c(-1,1)

LBetween[17:18,4]=c(-1,1)

LBetween[19:20,5]=c(-1,1)

LBetween[c("CE_Na", "DI_Na", "CE_Ve", "DI_Ve"),"CEvDI_Na-CEvDI_Ve"]= c(1, -1, -1, 1)

LBetween[c("CE_Na", "OB_Na", "CE_Ve", "OB_Ve"),"CEvOB_Na-CEvOB_Ve"]= c(1, -1, -1, 1)

LBetween[c("CE_Na", "OT_Na", "CE_Ve", "OT_Ve"),"CEvOT_Na-CEvOT_Ve"]= c(1, -1, -1, 1)

LBetween[c("CE_Na", "TE_Na", "CE_Ve", "TE_Ve"),"CEvTE_Na-CEvTE_Ve"]= c(1, -1, -1, 1)

LBetween[c("DI_Na", "OB_Na", "DI_Ve", "OB_Ve"),"DIvOB_Na-DIvOB_Ve"]= c(1, -1, -1, 1)

LBetween[c("DI_Na", "OT_Na", "DI_Ve", "OT_Ve"),"DIvOT_Na-DIvOT_Ve"]= c(1, -1, -1, 1)

LBetween[c("DI_Na", "TE_Na", "DI_Ve", "TE_Ve"),"DIvTE_Na-DIvTE_Ve"]= c(1, -1, -1, 1)

LBetween[c("OB_Na", "OT_Na", "OB_Ve", "OT_Ve"),"OBvOT_Na-OBvOT_Ve"]= c(1, -1, -1, 1)

LBetween[c("OB_Na", "TE_Na", "OB_Ve", "TE_Ve"),"OBvTE_Na-OBvTE_Ve"]= c(1, -1, -1, 1)

LBetween[c("OT_Na", "TE_Na", "OT_Ve", "TE_Ve"),"OTvTE_Na-OTvTE_Ve"]= c(1, -1, -1, 1)

alpha=0.05

screenTestBetween = glmQLFTest(fit_2way, contrast=LBetween)

pScreenBetween <- screenTestBetween$table$PValue

names(pScreenBetween) <- rownames(screenTestBetween$table)

sum(p.adjust(pScreenBetween, method="BH")<=0.05) #32

```

## III.4.3.2. Confirmation stage

```{r}

confirmationResultsHyp2 <- sapply(1:ncol(LBetween),function(i) glmQLFTest(fit_2way, contrast = LBetween[,i]), simplify=FALSE)

confirmationPListBetween <- lapply(confirmationResultsHyp2, function(x) x$table$PValue)

confirmationPBetween <- as.matrix(Reduce(f=cbind,confirmationPListBetween))

rownames(confirmationPBetween) <- rownames(confirmationResultsHyp2[[1]]$table)

colnames(confirmationPBetween) <- colnames(LBetween)

stageRObjBetween <- stageR(pScreen=pScreenBetween, pConfirmation=confirmationPBetween)

stageRAdjBetween <- stageWiseAdjustment(object=stageRObjBetween, method="holm", alpha=0.05)

resBetween <- getResults(stageRAdjBetween)

SignGenesBetween = colSums(resBetween) #number of DE genes in every contrast.

SignGenesBetween

adjusted_p_Between = getAdjustedPValues(stageRAdjBetween) #p-values to report in manuscript.pAdjScreen = pvalue of the global test, other columns are then the posthoc tests

resBetween_df = as.data.frame(resBetween) #resBrain is a matrix, to be able to subset, change it to a dataframe

OnlySignGenesBetween = resBetween_df[resBetween_df$padjScreen == 1,] #removes rows for which global test was non significant

### get fold changes for the significant gene(s)

Hyp2SignGenesBSvsCE=names(which(resBetween[,2]==1))

logFC_BSvsCE = confirmationResultsHyp2[[1]]$table[Hyp2SignGenesBSvsCE,] #adjust column to select correct contrast

BSvsCE_adjustedP = subset(adjusted_p_Between, rownames(adjusted_p_Between)%in%Hyp2SignGenesBSvsCE)

logFC_BSvsCE_adjP = merge(logFC_BSvsCE,BSvsCE_adjustedP, by = 0, all = TRUE)

logFC_BSvsCE_adjP$contrast = "BSvsCE" #add column to be able to differentiate after merging

logFC_BSvsCE_adjP = logFC_BSvsCE_adjP[,c(1,2,7,22)]

colnames(logFC_BSvsCE_adjP)[3] = "adj Pvalue"

Hyp2SignGenesBSvsDI=names(which(resBetween[,3]==1))

logFC_BSvsDI = confirmationResultsHyp2[[2]]$table[Hyp2SignGenesBSvsDI,] #adjust column to select correct contrast

BSvsDI_adjustedP = subset(adjusted_p_Between, rownames(adjusted_p_Between)%in%Hyp2SignGenesBSvsDI)

logFC_BSvsDI_adjP = merge(logFC_BSvsDI,BSvsDI_adjustedP, by = 0, all = TRUE)

logFC_BSvsDI_adjP$contrast = "BSvsDI"

logFC_BSvsDI_adjP = logFC_BSvsDI_adjP[,c(1,2,8,22)]

colnames(logFC_BSvsDI_adjP)[3] = "adj Pvalue"

Hyp2SignGenesBSvsOB=names(which(resBetween[,4]==1))

logFC_BSvsOB = confirmationResultsHyp2[[3]]$table[Hyp2SignGenesBSvsOB,] #adjust column to select correct contrast

BSvsOB_adjustedP = subset(adjusted_p_Between, rownames(adjusted_p_Between)%in%Hyp2SignGenesBSvsOB)

logFC_BSvsOB_adjP = merge(logFC_BSvsOB,BSvsOB_adjustedP, by = 0, all = TRUE)

logFC_BSvsOB_adjP$contrast = "BSvsOB"

logFC_BSvsOB_adjP = logFC_BSvsOB_adjP[,c(1,2,9,22)]

colnames(logFC_BSvsOB_adjP)[3] = "adj Pvalue"

Hyp2SignGenesBSvsTE=names(which(resBetween[,6]==1))

logFC_BSvsTE = confirmationResultsHyp2[[5]]$table[Hyp2SignGenesBSvsTE,] #adjust column to select correct contrast

BSvsTE_adjustedP = subset(adjusted_p_Between, rownames(adjusted_p_Between)%in%Hyp2SignGenesBSvsTE)

logFC_BSvsTE_adjP = merge(logFC_BSvsTE,BSvsTE_adjustedP, by = 0, all = TRUE)

logFC_BSvsTE_adjP$contrast = "BSvsTE"

logFC_BSvsTE_adjP = logFC_BSvsTE_adjP[,c(1,2,11,22)]

colnames(logFC_BSvsTE_adjP)[3] = "adj Pvalue"

Hyp2SignGenesCEvsDI=names(which(resBetween[,7]==1))

logFC_CEvsDI = confirmationResultsHyp2[[6]]$table[Hyp2SignGenesCEvsDI,] #adjust column to select correct contrast

CEvsDI_adjustedP = subset(adjusted_p_Between, rownames(adjusted_p_Between)%in%Hyp2SignGenesCEvsDI)

logFC_CEvsDI_adjP = merge(logFC_CEvsDI,CEvsDI_adjustedP, by = 0, all = TRUE)

logFC_CEvsDI_adjP$contrast = "CEvsDI"

logFC_CEvsDI_adjP = logFC_CEvsDI_adjP[,c(1,2,12,22)]

colnames(logFC_CEvsDI_adjP)[3] = "adj Pvalue"

Hyp2SignGenesCEvsOB=names(which(resBetween[,8]==1))

logFC_CEvsOB = confirmationResultsHyp2[[7]]$table[Hyp2SignGenesCEvsOB,] #adjust column to select correct contrast

CEvsOB_adjustedP = subset(adjusted_p_Between, rownames(adjusted_p_Between)%in%Hyp2SignGenesCEvsOB)

logFC_CEvsOB_adjP = merge(logFC_CEvsOB,CEvsOB_adjustedP, by = 0, all = TRUE)

logFC_CEvsOB_adjP$contrast = "CEvsOB"

logFC_CEvsOB_adjP = logFC_CEvsOB_adjP[,c(1,2,13,22)]

colnames(logFC_CEvsOB_adjP)[3] = "adj Pvalue"

Hyp2SignGenesCEvsOT=names(which(resBetween[,9]==1))

logFC_CEvsOT = confirmationResultsHyp2[[8]]$table[Hyp2SignGenesCEvsOT,] #adjust column to select correct contrast

CEvsOT_adjustedP = subset(adjusted_p_Between, rownames(adjusted_p_Between)%in%Hyp2SignGenesCEvsOT)

logFC_CEvsOT_adjP = merge(logFC_CEvsOT,CEvsOT_adjustedP, by = 0, all = TRUE)

logFC_CEvsOT_adjP$contrast = "CEvsOT"

logFC_CEvsOT_adjP = logFC_CEvsOT_adjP[,c(1,2,14,22)]

colnames(logFC_CEvsOT_adjP)[3] = "adj Pvalue"

Hyp2SignGenesCEvsTE=names(which(resBetween[,10]==1))

logFC_CEvsTE = confirmationResultsHyp2[[9]]$table[Hyp2SignGenesCEvsTE,] #adjust column to select correct contrast

CEvsTE_adjustedP = subset(adjusted_p_Between, rownames(adjusted_p_Between)%in%Hyp2SignGenesCEvsTE)

logFC_CEvsTE_adjP = merge(logFC_CEvsTE,CEvsTE_adjustedP, by = 0, all = TRUE)

logFC_CEvsTE_adjP$contrast = "CEvsTE"

logFC_CEvsTE_adjP = logFC_CEvsTE_adjP[,c(1,2,15,22)]

colnames(logFC_CEvsTE_adjP)[3] = "adj Pvalue"

Hyp2SignGenesDIvsOB=names(which(resBetween[,11]==1))

logFC_DIvsOB = confirmationResultsHyp2[[10]]$table[Hyp2SignGenesDIvsOB,] #adjust column to select correct contrast

DIvsOB_adjustedP = subset(adjusted_p_Between, rownames(adjusted_p_Between)%in%Hyp2SignGenesDIvsOB)

logFC_DIvsOB_adjP = merge(logFC_DIvsOB,DIvsOB_adjustedP, by = 0, all = TRUE)

logFC_DIvsOB_adjP$contrast = "DIvsOB"

logFC_DIvsOB_adjP = logFC_DIvsOB_adjP[,c(1,2,16,22)]

colnames(logFC_DIvsOB_adjP)[3] = "adj Pvalue"

Hyp2SignGenesDIvsOT=names(which(resBetween[,12]==1))

logFC_DIvsOT = confirmationResultsHyp2[[11]]$table[Hyp2SignGenesDIvsOT,] #adjust column to select correct contrast

DIvsOT_adjustedP = subset(adjusted_p_Between, rownames(adjusted_p_Between)%in%Hyp2SignGenesDIvsOT)

logFC_DIvsOT_adjP = merge(logFC_DIvsOT,DIvsOT_adjustedP, by = 0, all = TRUE)

logFC_DIvsOT_adjP$contrast = "DIvsOT"

logFC_DIvsOT_adjP = logFC_DIvsOT_adjP[,c(1,2,17,22)]

colnames(logFC_DIvsOT_adjP)[3] = "adj Pvalue"

Hyp2SignGenesDIvsTE=names(which(resBetween[,13]==1))

logFC_DIvsTE = confirmationResultsHyp2[[12]]$table[Hyp2SignGenesDIvsTE,] #adjust column to select correct contrast

DIvsTE_adjustedP = subset(adjusted_p_Between, rownames(adjusted_p_Between)%in%Hyp2SignGenesDIvsTE)

logFC_DIvsTE_adjP = merge(logFC_DIvsTE,DIvsTE_adjustedP, by = 0, all = TRUE)

logFC_DIvsTE_adjP$contrast = "DIvsTE"

logFC_DIvsTE_adjP = logFC_DIvsTE_adjP[,c(1,2,18,22)]

colnames(logFC_DIvsTE_adjP)[3] = "adj Pvalue"

Hyp2SignGenesOBvsOT=names(which(resBetween[,14]==1))

logFC_OBvsOT = confirmationResultsHyp2[[13]]$table[Hyp2SignGenesOBvsOT,] #adjust column to select correct contrast

OBvsOT_adjustedP = subset(adjusted_p_Between, rownames(adjusted_p_Between)%in%Hyp2SignGenesOBvsOT)

logFC_OBvsOT_adjP = merge(logFC_OBvsOT,OBvsOT_adjustedP, by = 0, all = TRUE)

logFC_OBvsOT_adjP$contrast = "OBvsOT"

logFC_OBvsOT_adjP = logFC_OBvsOT_adjP[,c(1,2,19,22)]

colnames(logFC_OBvsOT_adjP)[3] = "adj Pvalue"

Hyp2SignGenesOTvsTE=names(which(resBetween[,16]==1))

logFC_OTvsTE = confirmationResultsHyp2[[15]]$table[Hyp2SignGenesOTvsTE,] #adjust column to select correct contrast

OTvsTE_adjustedP = subset(adjusted_p_Between, rownames(adjusted_p_Between)%in%Hyp2SignGenesOTvsTE)

logFC_OTvsTE_adjP = merge(logFC_OTvsTE,OTvsTE_adjustedP, by = 0, all = TRUE)

logFC_OTvsTE_adjP$contrast = "OTvsTE"

logFC_OTvsTE_adjP = logFC_OTvsTE_adjP[,c(1,2,21,22)]

colnames(logFC_OTvsTE_adjP)[3] = "adj Pvalue"

logFC_adjP_interactiongenes = rbind(logFC_OTvsTE_adjP,logFC_OBvsOT_adjP, logFC_DIvsTE_adjP, logFC_DIvsOT_adjP, logFC_DIvsOB_adjP, logFC_CEvsTE_adjP, logFC_CEvsOT_adjP, logFC_CEvsOB_adjP, logFC_CEvsDI_adjP, logFC_BSvsTE_adjP, logFC_BSvsOB_adjP, logFC_BSvsDI_adjP, logFC_BSvsCE_adjP)

```

## III.4.3.2. Equivalence testing

```{r}

# credit to Aaron Lun for the equivalence test, at https://support.bioconductor.org/p/66283/

### reparametrizing the design matrix

contrastList = list()

for(i in 1:5) contrastList[[i]] = c(1:5)

for(i in 6:9) contrastList[[i]] = c(1:5)+(i-4)-1

contrastList[[10]] = c(1,4,7,9,10)

contrastList[[11]] = c(1,4,7,9,11)

contrastList[[12]] = c(1,4,7,9,12)

contrastList[[13]] = c(1,4,6,9,13)

contrastList[[14]] = c(1,4,6,9,14)

contrastList[[15]] = c(1,4,6,7,15)

fit=fit_2way

pEquivalence=matrix(NA,nrow=nrow(d),ncol=15)

for(i in 1:15){

cat(i)

# prepare design matrix

hlp=contrastAsCoef(design_2way, contrast=LBetween[,contrastList[[i]]])

design=hlp$design

y=d

orig.offs = log(y$samples$lib.size*y$samples$norm.factors)

if(i %in% 1:5){

y$offset <- as.vector(orig.offs + log(4)*design[,i])

fit.up <- glmFit(y, design)

lrt.up <- glmLRT(fit.up, coef=i)

y$offset <- as.vector(orig.offs - log(4)*design[,i])

fit.down <- glmFit(y, design)

lrt.down <- glmLRT(fit.down, coef=i)

p.up <- ifelse(lrt.up$table$logFC < 0, lrt.up$table$PValue/2, 1)

p.down <- ifelse(lrt.down$table$logFC > 0, lrt.down$table$PValue/2, 1)

p.tost <- pmax(p.up, p.down)

} else if(i %in% 6:15){

#for these contrasts, always the last of the rank 5 contrast matrix is the contrast of interest.

y$offset <- as.vector(orig.offs + log(4)*design[,5])

fit.up <- glmFit(y, design)

lrt.up <- glmLRT(fit.up, coef=5)

y$offset <- as.vector(orig.offs - log(4)*design[,5])

fit.down <- glmFit(y, design)

lrt.down <- glmLRT(fit.down, coef=5)

p.up <- ifelse(lrt.up$table$logFC < 0, lrt.up$table$PValue/2, 1)

p.down <- ifelse(lrt.down$table$logFC > 0, lrt.down$table$PValue/2, 1)

p.tost <- pmax(p.up, p.down)

}

pEquivalence[,i] = p.tost

}

## how many are equivalent in all contrasts

maxPEquivalence = apply(pEquivalence,1,max)

fdrAdjEquivalence = p.adjust(maxPEquivalence,"fdr")

mean(fdrAdjEquivalence<=0.05) #71% genes equivalent across all contrasts

#within sample significance

apply(pEquivalence,2,function(x) mean(p.adjust(x,"fdr")<=0.05))

```

#IV. Plots

##IV.2. Stage-wise analysis

## IV.2. Hypothesis 1: there is no differential expression between a brain part and the average of all other brain parts in O. nasuta and O. ventralis.

#the heatmap function requires the data to be a numerical matrix; we extract the DE genes from the DGE list, calculate cpm and return log2values

```{r}

nGenesToPlot=200

topGenes = rownames(getAdjustedPValues(stageRAdj,order=TRUE))[1:nGenesToPlot]

cpmBrain=cpm(d[topGenes,],log = TRUE) #subset the DGElist, calculate cpm, return log2 values (this makes comparison across orders of magnitude of expression possible)

hmcol <- colorRampPalette(brewer.pal(9, "GnBu"))(100)

heatmap.2(cpmBrain,dendrogram = "both",trace="none",col=hmcol,main="top 200 DE genes", cexRow = 0.2, cexCol = 0.5)

```

## top interaction genes

```{r}

topGenesInteraction = rownames(getAdjustedPValues(stageRAdjBetween,order=TRUE))[1:nGenesToPlot]

cpmBrain=cpm(d[topGenesInteraction,],log=TRUE)

hmcol <- colorRampPalette(brewer.pal(9, "GnBu"))(100)

heatmap.2(cpmBrain,trace="none",col=hmcol,main= "interaction genes")

nGenesToPlot=colSums(resBetween)[1] #nr of screening stage significant genes

topGenesInteraction = rownames(getAdjustedPValues(stageRAdjBetween))[1:nGenesToPlot]

cpmInter=cpm(d[topGenesInteraction,],log=TRUE)

plotHlp=cbind(cpmInter[,1:nGenesToPlot],NA,cpmInter[,-(1:nGenesToPlot)])

pdf("InteractionGenes.pdf", width = 7, height = 5)

par(mfrow=c(3,3),mar=c(2,5,3,1))

for(i in 1:nGenesToPlot)

{

plotHlp2=matrix(plotHlp[i,],nrow=5,ncol=12)

matplot(t(plotHlp2[,1:6]),type="b",ylim=range(plotHlp,na.rm=TRUE),xaxt="n",col=1,lty=1,pch=1,main=topGenesInteraction[i], ylab="log CPM")

matplot(t(plotHlp2[,-(1:6)]),type="b",ylim=range(plotHlp,na.rm=TRUE),xaxt="n",col=2,add=TRUE,lty=1,pch=2)

axis(at=1:6,labels=levels(brain),side=1)

}

dev.off()

plotHlp2

?gplots

```

#3. GO annotation

the count files contain the gene names, we obtained the GeneIDs from the genes using the genome annotation table from genbank (https://www.ncbi.nlm.nih.gov/genome/proteins/197?genome_assembly_id=293496).

```{r}

oreochromis = read.table ("Oniloticus_ProteinTable197_293496.txt", sep = "", comment.char="", quote = "\"", header = TRUE, fill = TRUE, stringsAsFactors = FALSE)

namesOnlySignGenes = rownames(OnlySignGenes) # all locus names of the significant genes

geneIDOnlySignGenesTable = oreochromis[oreochromis$Locus %in% namesOnlySignGenes,] #get only those that are DE

geneIDOnlySignGenesTableUnique = subset(geneIDOnlySignGenesTable, !duplicated(geneIDOnlySignGenesTable$GeneID)) # remove duplicated gene IDs

geneIDOnlySignGenes = c(geneIDOnlySignGenesTableUnique$GeneID) # put geneIDs in a vector to be used in biomart

library(biomaRt)

ensembl = useMart("ensembl",dataset="oniloticus_gene_ensembl")

filters = listFilters(ensembl) #to see which filters can be used

attributes = listAttributes(ensembl) #to see which parameters can be in the output table

go_all = getBM(attributes=c("refseq_peptide","refseq_peptide_predicted", "entrezgene", "go_id", "name_1006", "definition_1006", "namespace_1003"), filters = "entrezgene", values = geneIDOnlySignGenes, mart=ensembl)

go_all_goID = go_all[!go_all$go_id == "",] #remove rows with no go_id

go_all_goID_biologicalprocess = go_all_goID[go_all_goID$namespace_1003 == 'biological_process',] #get the biological function

write.table (go_all_goID_biologicalprocess, "GO_brainparts.txt", col.names = TRUE, row.names = FALSE, quote = FALSE, sep = ";")

go_all_goID_biologicalprocess_unique = go_all_goID_biologicalprocess[!duplicated(go_all_goID_biologicalprocess[,c(3:4)]),]

#get the GeneIDs from the loci DE only in each brainpart for O. nasuta:

geneID_BS_avgNa = oreochromis[oreochromis$Locus %in% BS_avgNa,]

geneID_BS_avgNa_unique = subset(geneID_BS_avgNa, !duplicated(geneID_BS_avgNa$GeneID))

geneID_CE_avgNa = oreochromis[oreochromis$Locus %in% CE_avgNa,]

geneID_CE_avgNa_unique = subset(geneID_CE_avgNa, !duplicated(geneID_CE_avgNa$GeneID))

geneID_TE_avgNa = oreochromis[oreochromis$Locus %in% TE_avgNa,]

geneID_TE_avgNa_unique = subset(geneID_TE_avgNa, !duplicated(geneID_TE_avgNa$GeneID))

geneID_OB_avgNa = oreochromis[oreochromis$Locus %in% OB_avgNa,]

geneID_OB_avgNa_unique = subset(geneID_OB_avgNa, !duplicated(geneID_OB_avgNa$GeneID))

geneID_OT_avgNa = oreochromis[oreochromis$Locus %in% OT_avgNa,]

geneID_OT_avgNa_unique = subset(geneID_OT_avgNa, !duplicated(geneID_OT_avgNa$GeneID))

geneID_DI_avgNa = oreochromis[oreochromis$Locus %in% DI_avgNa,]

geneID_DI_avgNa_unique = subset(geneID_DI_avgNa, !duplicated(geneID_DI_avgNa$GeneID))

#get the GeneIDs from the loci DE in each brainpart for O. ventralis:

geneID_BS_avgVe = oreochromis[oreochromis$Locus %in% BS_avgVe,]

geneID_BS_avgVe_unique = subset(geneID_BS_avgVe, !duplicated(geneID_BS_avgVe$GeneID))

geneID_CE_avgVe = oreochromis[oreochromis$Locus %in% CE_avgVe,]

geneID_CE_avgVe_unique = subset(geneID_CE_avgVe, !duplicated(geneID_CE_avgVe$GeneID))

geneID_TE_avgVe = oreochromis[oreochromis$Locus %in% TE_avgVe,]

geneID_TE_avgVe_unique = subset(geneID_TE_avgVe, !duplicated(geneID_TE_avgVe$GeneID))

geneID_OB_avgVe = oreochromis[oreochromis$Locus %in% OB_avgVe,]

geneID_OB_avgVe_unique = subset(geneID_OB_avgVe, !duplicated(geneID_OB_avgVe$GeneID))

geneID_OT_avgVe = oreochromis[oreochromis$Locus %in% OT_avgVe,]

geneID_OT_avgVe_unique = subset(geneID_OT_avgVe, !duplicated(geneID_OT_avgVe$GeneID))

geneID_DI_avgVe_unique = subset(geneID_DI_avgVe, !duplicated(geneID_DI_avgVe$GeneID))

geneID_DI_avgVe = oreochromis[oreochromis$Locus %in% DI_avgVe,]

#get the Gene ID for the DE genes with logFC > 2 in both species

gene_ID_logFC2_BS = oreochromis[oreochromis$Locus %in% logFC2_BS$Row.names,]

gene_ID_logFC2_BS_unique = subset(gene_ID_logFC2_BS, !duplicated(gene_ID_logFC2_BS$GeneID))

go_logFC2_BS = subset(go_all_goID_biologicalprocess_unique,go_all_goID_biologicalprocess_unique$entrezgene %in% gene_ID_logFC2_BS_unique$GeneID)

go_logFC2_BS = go_logFC2_BS[,3:5]

go_logFC2_BS = merge(gene_ID_logFC2_BS_unique, go_logFC2_BS, by.x = "GeneID", by.y = "entrezgene")

go_logFC2_BS = go_logFC2_BS[,c(1,6,9,10)]

go_logFC2_BS = merge(go_logFC2_BS,logFC2_BS, by.x = "Locus", by.y = "Row.names")

colnames(go_logFC2_BS) = c("Locus", "GeneID", "GoID", "Biological Process", "LogFC_Na", "AdjustedPvalue_Na", "LogFC_Ve", "AdjustedPvalue_Ve")

gene_ID_logFC2_CE = oreochromis[oreochromis$Locus %in% logFC2_CE$Row.names,]

gene_ID_logFC2_CE_unique = subset(gene_ID_logFC2_CE, !duplicated(gene_ID_logFC2_CE$GeneID))

go_logFC2_CE = subset(go_all_goID_biologicalprocess_unique,go_all_goID_biologicalprocess_unique$entrezgene %in% gene_ID_logFC2_CE_unique$GeneID)

go_logFC2_CE = go_logFC2_CE[,3:5]

go_logFC2_CE = merge(gene_ID_logFC2_CE_unique, go_logFC2_CE, by.x = "GeneID", by.y = "entrezgene")

go_logFC2_CE = go_logFC2_CE[,c(1,6,9,10)]

go_logFC2_CE = merge(go_logFC2_CE,logFC2_CE, by.x = "Locus", by.y = "Row.names")

colnames(go_logFC2_CE) = c("Locus", "GeneID", "GoID", "Biological Process", "LogFC_Na", "AdjustedPvalue_Na", "LogFC_Ve", "AdjustedPvalue_Ve")

gene_ID_logFC2_DI = oreochromis[oreochromis$Locus %in% logFC2_DI$Row.names,]

gene_ID_logFC2_DI_unique = subset(gene_ID_logFC2_DI, !duplicated(gene_ID_logFC2_DI$GeneID))

go_logFC2_DI = subset(go_all_goID_biologicalprocess_unique,go_all_goID_biologicalprocess_unique$entrezgene %in% gene_ID_logFC2_DI_unique$GeneID)

go_logFC2_DI = go_logFC2_DI[,3:5]

go_logFC2_DI = merge(gene_ID_logFC2_DI_unique, go_logFC2_DI, by.x = "GeneID", by.y = "entrezgene")

go_logFC2_DI = go_logFC2_DI[,c(1,6,9,10)]

go_logFC2_DI = merge(go_logFC2_DI,logFC2_DI, by.x = "Locus", by.y = "Row.names")

colnames(go_logFC2_DI) = c("Locus", "GeneID", "GoID", "Biological Process", "LogFC_Na", "AdjustedPvalue_Na", "LogFC_Ve", "AdjustedPvalue_Ve")

gene_ID_logFC2_OB = oreochromis[oreochromis$Locus %in% logFC2_OB$Row.names,]

gene_ID_logFC2_OB_unique = subset(gene_ID_logFC2_OB, !duplicated(gene_ID_logFC2_OB$GeneID))

go_logFC2_OB = subset(go_all_goID_biologicalprocess_unique,go_all_goID_biologicalprocess_unique$entrezgene %in% gene_ID_logFC2_OB_unique$GeneID)

go_logFC2_OB = go_logFC2_OB[,3:5]

go_logFC2_OB = merge(gene_ID_logFC2_OB_unique, go_logFC2_OB, by.x = "GeneID", by.y = "entrezgene")

go_logFC2_OB = go_logFC2_OB[,c(1,6,9,10)]

go_logFC2_OB = merge(go_logFC2_OB,logFC2_OB, by.x = "Locus", by.y = "Row.names")

colnames(go_logFC2_OB) = c("Locus", "GeneID", "GoID", "Biological Process", "LogFC_Na", "AdjustedPvalue_Na", "LogFC_Ve", "AdjustedPvalue_Ve")

gene_ID_logFC2_OT = oreochromis[oreochromis$Locus %in% logFC2_OT$Row.names,]

gene_ID_logFC2_OT_unique = subset(gene_ID_logFC2_OT, !duplicated(gene_ID_logFC2_OT$GeneID))

go_logFC2_OT = subset(go_all_goID_biologicalprocess_unique,go_all_goID_biologicalprocess_unique$entrezgene %in% gene_ID_logFC2_OT_unique$GeneID)

go_logFC2_OT = go_logFC2_OT[,3:5]

go_logFC2_OT = merge(gene_ID_logFC2_OT_unique, go_logFC2_OT, by.x = "GeneID", by.y = "entrezgene")

go_logFC2_OT = go_logFC2_OT[,c(1,6,9,10)]

go_logFC2_OT = merge(go_logFC2_OT,logFC2_OT, by.x = "Locus", by.y = "Row.names")

colnames(go_logFC2_OT) = c("Locus", "GeneID", "GoID", "Biological Process", "LogFC_Na", "AdjustedPvalue_Na", "LogFC_Ve", "AdjustedPvalue_Ve")

gene_ID_logFC2_TE = oreochromis[oreochromis$Locus %in% logFC2_TE$Row.names,]

gene_ID_logFC2_TE_unique = subset(gene_ID_logFC2_TE, !duplicated(gene_ID_logFC2_TE$GeneID))

go_logFC2_TE = subset(go_all_goID_biologicalprocess_unique,go_all_goID_biologicalprocess_unique$entrezgene %in% gene_ID_logFC2_TE_unique$GeneID)

go_logFC2_TE = go_logFC2_TE[,3:5]

go_logFC2_TE = merge(gene_ID_logFC2_TE_unique, go_logFC2_TE, by.x = "GeneID", by.y = "entrezgene")

go_logFC2_TE = go_logFC2_TE[,c(1,6,9,10)]

go_logFC2_TE = merge(go_logFC2_TE,logFC2_TE, by.x = "Locus", by.y = "Row.names")

colnames(go_logFC2_TE) = c("Locus", "GeneID", "GoID", "Biological Process", "LogFC_Na", "AdjustedPvalue_Na", "LogFC_Ve", "AdjustedPvalue_Ve")

write.table (go_logFC2_BS, "logFC2_BS_all.txt", col.names = TRUE, row.names = FALSE, quote = FALSE, sep = ";")

write.table (go_logFC2_CE, "logFC2_CE_all.txt", col.names = TRUE, row.names = FALSE, quote = FALSE, sep = ";")

write.table (go_logFC2_DI, "logFC2_DI_all.txt", col.names = TRUE, row.names = FALSE, quote = FALSE, sep = ";")

write.table (go_logFC2_OB, "logFC2_OB_all.txt", col.names = TRUE, row.names = FALSE, quote = FALSE, sep = ";")

write.table (go_logFC2_OT, "logFC2_OT_all.txt", col.names = TRUE, row.names = FALSE, quote = FALSE, sep = ";")

write.table (go_logFC2_TE, "logFC2_TE_all.txt", col.names = TRUE, row.names = FALSE, quote = FALSE, sep = ";")

##geneID and GO annotation interaction genes

geneID_interactiongenes = oreochromis[oreochromis$Locus %in% logFC_adjP_interactiongenes$Row.names,]

geneID_interactiongenes_unique = subset(geneID_interactiongenes, !duplicated(geneID_interactiongenes$GeneID))

go_interactiongenes = subset(go_all_goID_biologicalprocess_unique,go_all_goID_biologicalprocess_unique$entrezgene %in% geneID_interactiongenes_unique$GeneID)

go_interactiongenes = go_interactiongenes[,3:5]

go_geneID_interactiongenes = merge(geneID_interactiongenes_unique, go_interactiongenes, by.x = "GeneID", by.y = "entrezgene", all=TRUE)

go_logFC_interactiongenes = merge(logFC_adjP_interactiongenes, go_geneID_interactiongenes, by.x = "Row.names", by.y = "Locus", all=TRUE)

go_logFC_interactiongenes = go_logFC_interactiongenes[,-(6:11)]

write.table (go_logFC_interactiongenes, "go_interactiongenes.txt", col.names = TRUE, row.names = FALSE, quote = FALSE, sep = ";")

```

# GO enrichment analysis

Make a set of DE genes for every contrast and compare with all DE genen in all contrasts. We use CAMERA to perform the enrichment analysis.

```{r}

#enrichment analysis for O. nasuta

d_allDE_nasuta = d[allSignGenes_Na,] #subset the DGElist to only the significant genes #6847

geneID_allDE_nasuta = oreochromis[oreochromis$Locus %in% allSignGenes_Na,]

geneID_allDE_nasuta_unique = subset(geneID_allDE_nasuta, !duplicated(geneID_allDE_nasuta$GeneID)) #6749

go_all_biologicalprocess = go_all[go_all$namespace_1003 == 'biological_process',]

go_allDE_nasuta = subset(go_all_goID_biologicalprocess_unique,go_all_goID_biologicalprocess_unique$entrezgene %in% geneID_allDE_nasuta_unique$GeneID)

#to add locus names to geneID and GO, merge two dataframes

LocusID_allDE_nasuta = geneID_allDE_nasuta_unique[,5:6] # get the colmuns with locus and geneID so that only the locus column is added to the GO dataframe

Locus_geneID_allDE_nasuta = merge(LocusID_allDE_nasuta, go_allDE_nasuta, by.x = "GeneID", by.y = "entrezgene")

write.table (Locus_geneID_allDE_nasuta, "Locus_geneID_allDE_nasuta.txt", col.names = TRUE, row.names = FALSE, quote = FALSE, sep = ";")

#create gene sets based on GO terms

UniqueGeneSets_nasuta=unique(Locus_geneID_allDE_nasuta$go_id)

length(UniqueGeneSets_nasuta)#1720

geneSetList_nasuta=list()

for(i in 1:length(UniqueGeneSets_nasuta)){

geneSetList_nasuta[[i]] = Locus_geneID_allDE_nasuta$Locus.x[Locus_geneID_allDE_nasuta$go_id==UniqueGeneSets_nasuta[i]]

}

names(geneSetList_nasuta)=UniqueGeneSets_nasuta

#CEREBELLUM (not subsetted)

c2.ind_nasuta <- ids2indices(geneSetList_nasuta, rownames(d))

resCAMERA_CENa = camera(d,index=c2.ind_nasuta,design=design_2way,contrast=LBrain[,3], inter.gene.cor = 0.05)

table(resCAMERA_CENa$FDR < 0.05) #13

write.table (resCAMERA_CENa, "resCAMERA_CENa.txt", col.names = TRUE, row.names = TRUE, quote = FALSE, sep = ";")

#BRAINSTEM (not subsetted)

resCAMERA_BSNa = camera(d,index=c2.ind_nasuta,design=design_2way,contrast=LBrain[,1], inter.gene.cor = 0.05)

table(resCAMERA_BSNa$FDR < 0.05) #75

#DIENCEPHALON (not subsetted)

resCAMERA_DINa = camera(d,index=c2.ind_nasuta,design=design_2way,contrast=LBrain[,5], inter.gene.cor = 0.05)

table(resCAMERA_DINa$FDR < 0.05) #46

#OlFACTORY BULBS (not subsetted)

resCAMERA_OBNa = camera(d,index=c2.ind_nasuta,design=design_2way,contrast=LBrain[,7], inter.gene.cor = 0.05)

table(resCAMERA_OBNa$FDR < 0.05) #15

#OPTIC TECTUM (not subsetted)

resCAMERA_OTNa = camera(d,index=c2.ind_nasuta,design=design_2way,contrast=LBrain[,9], inter.gene.cor = 0.05)

table(resCAMERA_OTNa$FDR < 0.05) #69

#TELENCEPHALON (not subsetted)

resCAMERA_TENa = camera(d,index=c2.ind_nasuta,design=design_2way,contrast=LBrain[,11], inter.gene.cor = 0.05)

table(resCAMERA_TENa$FDR < 0.05) #16

#enrichment analysis for 0. ventralis

d_allDE_ventralis = d[allSignGenes_Ve,] #subset the DGElist to only the significant genes #7098

geneID_allDE_ventralis = oreochromis[oreochromis$Locus %in% allSignGenes_Ve,]

geneID_allDE_ventralis_unique = subset(geneID_allDE_ventralis, !duplicated(geneID_allDE_ventralis$GeneID)) #6967

go_all_biologicalprocess = go_all[go_all$namespace_1003 == 'biological_process',]

go_allDE_ventralis = subset(go_all_goID_biologicalprocess_unique,go_all_goID_biologicalprocess_unique$entrezgene %in% geneID_allDE_ventralis_unique$GeneID)

#to add locus names to geneID and GO, merge two dataframes

LocusID_allDE_ventralis = geneID_allDE_ventralis_unique[,5:6] # get the colmuns with locus and geneID so that only the locus column is added to the GO dataframe

Locus_geneID_allDE_ventralis = merge(LocusID_allDE_ventralis, go_allDE_ventralis, by.x = "GeneID", by.y = "entrezgene")

write.table (Locus_geneID_allDE_ventralis, "Locus_geneID_allDE_ventralis.txt", col.names = TRUE, row.names = FALSE, quote = FALSE, sep = ";")

#create gene sets based on GO terms

UniqueGeneSets_ventralis=unique(Locus_geneID_allDE_ventralis$go_id)

length(UniqueGeneSets_ventralis) #1734

geneSetList_ventralis=list()

for(i in 1:length(UniqueGeneSets_ventralis)){

geneSetList_ventralis[[i]] = Locus_geneID_allDE_ventralis$Locus[Locus_geneID_allDE_ventralis$go_id==UniqueGeneSets_ventralis[i]]

}

names(geneSetList_ventralis)=UniqueGeneSets_ventralis

#CEREBELLUM (not subsetted)

c2.ind_ventralis <- ids2indices(geneSetList_ventralis, rownames(d))

resCAMERA_CEVe = camera(d,index=c2.ind_ventralis,design=design_2way,contrast=LBrain[,4], inter.gene.cor = 0.05)

table(resCAMERA_CEVe$FDR < 0.05) #8

write.table (resCAMERA_CEVe, "resCAMERA_CEVe.txt", col.names = TRUE, row.names = TRUE, quote = FALSE, sep = ";")

#BRAINSTEM (not subsetted)

resCAMERA_BSVe = camera(d,index=c2.ind_ventralis,design=design_2way,contrast=LBrain[,2], inter.gene.cor = 0.05)

table(resCAMERA_BSVe$FDR < 0.05) #64

#DIENCEPHALON (not subsetted)

resCAMERA_DIVe = camera(d,index=c2.ind_ventralis,design=design_2way,contrast=LBrain[,6], inter.gene.cor = 0.05)

table(resCAMERA_DIVe$FDR < 0.05) #41

#OlFACTORY BULBS (not subsetted)

resCAMERA_OBVe = camera(d,index=c2.ind_ventralis,design=design_2way,contrast=LBrain[,8], inter.gene.cor = 0.05)

table(resCAMERA_OBVe$FDR < 0.05) #21

#OPTIC TECTUM (not subsetted)

resCAMERA_OTVe = camera(d,index=c2.ind_ventralis,design=design_2way,contrast=LBrain[,10], inter.gene.cor = 0.05)

table(resCAMERA_OTVe$FDR < 0.05) #72

#TELENCEPHALON (not subsetted)

resCAMERA_TEVe = camera(d,index=c2.ind_ventralis,design=design_2way,contrast=LBrain[,12], inter.gene.cor = 0.05)

table(resCAMERA_TEVe$FDR < 0.05) #20

```

#GOplot: visualisation of the enrichment analysis

https://cran.r-project.org/web/packages/GOplot/vignettes/GOplot_vignette.html

```{r}

##for O. nasuta

geneSetList_nasuta_df = data.frame(GO_ID=names(geneSetList_nasuta),genes=unlist(lapply(geneSetList_nasuta,paste,collapse=","))) #convert the genesetlist two two columns

geneSetList_nasuta_df = geneSetList_nasuta_df[order(geneSetList_nasuta_df$GO_ID),]#contains all significant genes of all brain parts per GO term, but does not contain the GO term itself. It does contain GO ID

geneSetList_GO_nasuta = subset(go_all_goID_biologicalprocess_unique, go_all_goID_biologicalprocess_unique$go_id %in% geneSetList_nasuta_df$GO_ID)

geneSetList_GO_nasuta_unique = subset(geneSetList_GO_nasuta,!duplicated(geneSetList_GO_nasuta$go_id))

geneSetList_GO_nasuta_unique = geneSetList_GO_nasuta_unique[order(geneSetList_GO_nasuta_unique$go_id),]#sort based on GO_ID

#make input file for GOplot for TENa

resCAMERA_TENa$GO_ID = rownames(resCAMERA_TENa)

resCAMERA_TENa = resCAMERA_TENa[order(resCAMERA_TENa$GO_ID),] # sort based on GO_ID

goplot_TENa = cbind(resCAMERA_TENa,geneSetList_nasuta_df, geneSetList_GO_nasuta_unique$name_1006)#merges results from CAMERA for TE, genes in the GO lists and the GO terms (required for GO plot)

goplot_TENa$Category = "BP"

goplot_TENa = goplot_TENa[,c(5,7:10)]

goplot_TENa = goplot_TENa[,c(5,2,4,3,1)]

colnames(goplot_TENa) = c("category", "ID", "term", "genes", "adj_pval")

#get FC for all genes in TE

Hyp1AllGenes=rownames(resBrain)

colnames(LBrain)

logFC_All_TEavgNa = confirmationResultsHyp1[[11]]$table[Hyp1AllGenes,]

logFC_All_TEavgNa$ID = rownames(logFC_All_TEavgNa)

#make input file for GOplot for DINa

resCAMERA_DINa$GO_ID = rownames(resCAMERA_DINa)

resCAMERA_DINa = resCAMERA_DINa[order(resCAMERA_DINa$GO_ID),]

goplot_DINa = cbind(resCAMERA_DINa,geneSetList_nasuta_df, geneSetList_GO_nasuta_unique$name_1006)

goplot_DINa$Category = "BP"

goplot_DINa = goplot_DINa[,c(5,7:10)]

goplot_DINa = goplot_DINa[,c(5,2,4,3,1)]

colnames(goplot_DINa) = c("category", "ID", "term", "genes", "adj_pval")

colnames(LBrain)

logFC_All_DIavgNa = confirmationResultsHyp1[[5]]$table[Hyp1AllGenes,]

logFC_All_DIavgNa$ID = rownames(logFC_All_DIavgNa)

#make input file for GOplot for CENa

resCAMERA_CENa$GO_ID = rownames(resCAMERA_CENa)

resCAMERA_CENa = resCAMERA_CENa[order(resCAMERA_CENa$GO_ID),]

goplot_CENa = cbind(resCAMERA_CENa,geneSetList_nasuta_df, geneSetList_GO_nasuta_unique$name_1006)

goplot_CENa$Category = "BP"

goplot_CENa = goplot_CENa[,c(5,7:10)]

goplot_CENa = goplot_CENa[,c(5,2,4,3,1)]

colnames(goplot_CENa) = c("category", "ID", "term", "genes", "adj_pval")

colnames(LBrain)

logFC_All_CEavgNa = confirmationResultsHyp1[[3]]$table[Hyp1AllGenes,]

logFC_All_CEavgNa$ID = rownames(logFC_All_CEavgNa)

#make input file for GOplot for OBNa

resCAMERA_OBNa$GO_ID = rownames(resCAMERA_OBNa)

resCAMERA_OBNa = resCAMERA_OBNa[order(resCAMERA_OBNa$GO_ID),]

goplot_OBNa = cbind(resCAMERA_OBNa,geneSetList_nasuta_df, geneSetList_GO_nasuta_unique$name_1006)

goplot_OBNa$Category = "BP"

goplot_OBNa = goplot_OBNa[,c(5,7:10)]

goplot_OBNa = goplot_OBNa[,c(5,2,4,3,1)]

colnames(goplot_OBNa) = c("category", "ID", "term", "genes", "adj_pval")

colnames(LBrain)

logFC_All_OBavgNa = confirmationResultsHyp1[[7]]$table[Hyp1AllGenes,]

logFC_All_OBavgNa$ID = rownames(logFC_All_OBavgNa)

#make input file for GOplot for OTNa

resCAMERA_OTNa$GO_ID = rownames(resCAMERA_OTNa)

resCAMERA_OTNa = resCAMERA_OTNa[order(resCAMERA_OTNa$GO_ID),]

goplot_OTNa = cbind(resCAMERA_OTNa,geneSetList_nasuta_df, geneSetList_GO_nasuta_unique$name_1006)

goplot_OTNa$Category = "BP"

goplot_OTNa = goplot_OTNa[,c(5,7:10)]

goplot_OTNa = goplot_OTNa[,c(5,2,4,3,1)]

colnames(goplot_OTNa) = c("category", "ID", "term", "genes", "adj_pval")

colnames(LBrain)

logFC_All_OTavgNa = confirmationResultsHyp1[[9]]$table[Hyp1AllGenes,]

logFC_All_OTavgNa$ID = rownames(logFC_All_OTavgNa)

#make input file for GOplot for BSNa

resCAMERA_BSNa$GO_ID = rownames(resCAMERA_BSNa)

resCAMERA_BSNa = resCAMERA_BSNa[order(resCAMERA_BSNa$GO_ID),]

goplot_BSNa = cbind(resCAMERA_BSNa,geneSetList_nasuta_df, geneSetList_GO_nasuta_unique$name_1006)

goplot_BSNa$Category = "BP"

goplot_BSNa = goplot_BSNa[,c(5,7:10)]

goplot_BSNa = goplot_BSNa[,c(5,2,4,3,1)]

colnames(goplot_BSNa) = c("category", "ID", "term", "genes", "adj_pval")

colnames(LBrain)

logFC_All_BSavgNa = confirmationResultsHyp1[[1]]$table[Hyp1AllGenes,]

logFC_All_BSavgNa$ID = rownames(logFC_All_BSavgNa)

##for O. ventralis

geneSetList_ventralis_df = data.frame(GO_ID=names(geneSetList_ventralis),genes=unlist(lapply(geneSetList_ventralis,paste,collapse=","))) #convert the genesetlist two two columns

geneSetList_ventralis_df = geneSetList_ventralis_df[order(geneSetList_ventralis_df$GO_ID),]

geneSetList_GO_ventralis = subset(go_all_goID_biologicalprocess_unique, go_all_goID_biologicalprocess_unique$go_id %in% geneSetList_ventralis_df$GO_ID)

geneSetList_GO_ventralis_unique = subset(geneSetList_GO_ventralis,!duplicated(geneSetList_GO_ventralis$go_id))

geneSetList_GO_ventralis_unique = geneSetList_GO_ventralis_unique[order(geneSetList_GO_ventralis_unique$go_id),]#sort based on GO_ID

#make input file for GOplot for TENa

resCAMERA_TEVe$GO_ID = rownames(resCAMERA_TEVe)

resCAMERA_TEVe = resCAMERA_TEVe[order(resCAMERA_TEVe$GO_ID),] # sort based on GO_ID

goplot_TEVe = cbind(resCAMERA_TEVe,geneSetList_ventralis_df, geneSetList_GO_ventralis_unique$name_1006)

goplot_TEVe$Category = "BP"

goplot_TEVe = goplot_TEVe[,c(5,7:10)]

goplot_TEVe = goplot_TEVe[,c(5,2,4,3,1)]

colnames(goplot_TEVe) = c("category", "ID", "term", "genes", "adj_pval")

#get FC for all genes in TE

Hyp1AllGenes=rownames(resBrain)

colnames(LBrain)#om te zien welke lijst te selecteren

logFC_All_TEavgVe = confirmationResultsHyp1[[12]]$table[Hyp1AllGenes,]

logFC_All_TEavgVe$ID = rownames(logFC_All_TEavgVe)

#make input file for GOplot for DIVe

resCAMERA_DIVe$GO_ID = rownames(resCAMERA_DIVe)

resCAMERA_DIVe = resCAMERA_DIVe[order(resCAMERA_DIVe$GO_ID),]

goplot_DIVe = cbind(resCAMERA_DIVe,geneSetList_ventralis_df, geneSetList_GO_ventralis_unique$name_1006)

goplot_DIVe$Category = "BP"

goplot_DIVe = goplot_DIVe[,c(5,7:10)]

goplot_DIVe = goplot_DIVe[,c(5,2,4,3,1)]

colnames(goplot_DIVe) = c("category", "ID", "term", "genes", "adj_pval")

colnames(LBrain)

logFC_All_DIavgVe = confirmationResultsHyp1[[6]]$table[Hyp1AllGenes,]

logFC_All_DIavgVe$ID = rownames(logFC_All_DIavgVe)

#make input file for GOplot for CEVe

resCAMERA_CEVe$GO_ID = rownames(resCAMERA_CEVe)

resCAMERA_CEVe = resCAMERA_CEVe[order(resCAMERA_CEVe$GO_ID),]

goplot_CEVe = cbind(resCAMERA_CEVe,geneSetList_ventralis_df, geneSetList_GO_ventralis_unique$name_1006)

goplot_CEVe$Category = "BP"

goplot_CEVe = goplot_CEVe[,c(5,7:10)]

goplot_CEVe = goplot_CEVe[,c(5,2,4,3,1)]

colnames(goplot_CEVe) = c("category", "ID", "term", "genes", "adj_pval")

colnames(LBrain)

logFC_All_CEavgVe = confirmationResultsHyp1[[4]]$table[Hyp1AllGenes,]

logFC_All_CEavgVe$ID = rownames(logFC_All_CEavgVe)

#make input file for GOplot for OBVe

resCAMERA_OBVe$GO_ID = rownames(resCAMERA_OBVe)

resCAMERA_OBVe = resCAMERA_OBVe[order(resCAMERA_OBVe$GO_ID),]

goplot_OBVe = cbind(resCAMERA_OBVe,geneSetList_ventralis_df, geneSetList_GO_ventralis_unique$name_1006)

goplot_OBVe$Category = "BP"

goplot_OBVe = goplot_OBVe[,c(5,7:10)]

goplot_OBVe = goplot_OBVe[,c(5,2,4,3,1)]

colnames(goplot_OBVe) = c("category", "ID", "term", "genes", "adj_pval")

colnames(LBrain)

logFC_All_OBavgVe = confirmationResultsHyp1[[8]]$table[Hyp1AllGenes,]

logFC_All_OBavgVe$ID = rownames(logFC_All_OBavgVe)

#make input file for GOplot for OTVe

resCAMERA_OTVe$GO_ID = rownames(resCAMERA_OTVe)

resCAMERA_OTVe = resCAMERA_OTVe[order(resCAMERA_OTVe$GO_ID),]

goplot_OTVe = cbind(resCAMERA_OTVe,geneSetList_ventralis_df, geneSetList_GO_ventralis_unique$name_1006)

goplot_OTVe$Category = "BP"

goplot_OTVe = goplot_OTVe[,c(5,7:10)]

goplot_OTVe = goplot_OTVe[,c(5,2,4,3,1)]

colnames(goplot_OTVe) = c("category", "ID", "term", "genes", "adj_pval")

colnames(LBrain)

logFC_All_OTavgVe = confirmationResultsHyp1[[10]]$table[Hyp1AllGenes,]

logFC_All_OTavgVe$ID = rownames(logFC_All_OTavgVe)

#make input file for GOplot for BSVe

resCAMERA_BSVe$GO_ID = rownames(resCAMERA_BSVe)

resCAMERA_BSVe = resCAMERA_BSVe[order(resCAMERA_BSVe$GO_ID),]

goplot_BSVe = cbind(resCAMERA_BSVe,geneSetList_ventralis_df, geneSetList_GO_ventralis_unique$name_1006)

goplot_BSVe$Category = "BP"

goplot_BSVe = goplot_BSVe[,c(5,7:10)]

goplot_BSVe = goplot_BSVe[,c(5,2,4,3,1)]

colnames(goplot_BSVe) = c("category", "ID", "term", "genes", "adj_pval")

colnames(LBrain)

logFC_All_BSavgVe = confirmationResultsHyp1[[2]]$table[Hyp1AllGenes,]

logFC_All_BSavgVe$ID = rownames(logFC_All_BSavgVe)

library(GOplot)

circ_TENa = circle_dat(goplot_TENa, logFC_All_TEavgNa) #column names need to be exactly as defined above - no capital letter

circ_DINa = circle_dat(goplot_DINa, logFC_All_DIavgNa)

circ_CENa = circle_dat(goplot_CENa, logFC_All_CEavgNa)

circ_OBNa = circle_dat(goplot_OBNa, logFC_All_OBavgNa)

circ_OTNa = circle_dat(goplot_OTNa, logFC_All_OTavgNa)

circ_BSNa = circle_dat(goplot_BSNa, logFC_All_BSavgNa)

circ_TEVe = circle_dat(goplot_TEVe, logFC_All_TEavgVe)

circ_DIVe = circle_dat(goplot_DIVe, logFC_All_DIavgVe)

circ_CEVe = circle_dat(goplot_CEVe, logFC_All_CEavgVe)

circ_OBVe = circle_dat(goplot_OBVe, logFC_All_OBavgVe)

circ_OTVe = circle_dat(goplot_OTVe, logFC_All_OTavgVe)

circ_BSVe = circle_dat(goplot_BSVe, logFC_All_BSavgVe)

library(ggrepel)

pdf("GObubble_TENa.pdf", width = 7, height = 5)

GOBubble(circ_TENa, labels = 1.4, title = "TE_Na", table.legend = FALSE)

dev.off()

pdf("GObubble_DINa.pdf", width = 7, height = 5)

GOBubble(circ_DINa, labels = 1.4, title = "DI_Na", table.legend = FALSE)

dev.off()

pdf("GObubble_CENa.pdf", width = 7, height = 5)

GOBubble(circ_CENa, labels = 1.4, title = "CE_Na", table.legend = FALSE)

dev.off()

pdf("GObubble_OBNa.pdf", width = 7, height = 5)

GOBubble(circ_OBNa, labels = 1.4, title = "OB_Na", table.legend = FALSE)

dev.off()

pdf("GObubble_OTNa.pdf", width = 7, height = 5)

GOBubble(circ_OTNa, labels = 1.4, title = "OT_Na", table.legend = FALSE)

dev.off()

pdf("GObubble_BSVe.pdf", width = 7, height = 5)

GOBubble(circ_BSVe, labels = 1.4, title = "BS_Ve", table.legend = FALSE)

dev.off()

pdf("GObubble_TEVe.pdf", width = 7, height = 5)

GOBubble(circ_TEVe, labels = 1.4, title = "TE_Ve", table.legend = FALSE)

dev.off()

pdf("GObubble_DIVe.pdf", width = 7, height = 5)

GOBubble(circ_DIVe, labels = 1.4, title = "DI_Ve", table.legend = FALSE)

dev.off()

pdf("GObubble_CEVe.pdf", width = 7, height = 5)

GOBubble(circ_CEVe, labels = 1.4, title = "CE_Ve", table.legend = FALSE)

dev.off()

pdf("GObubble_OBVe.pdf", width = 7, height = 5)

GOBubble(circ_OBVe, labels = 1.4, title = "OB_Ve", table.legend = FALSE)

dev.off()

pdf("GObubble_OTVe.pdf", width = 7, height = 5)

GOBubble(circ_OTVe, labels = 1.4, title = "OT_Ve", table.legend = FALSE)

dev.off()

pdf("GObubble_BSVe.pdf", width = 7, height = 5)

GOBubble(circ_BSVe, labels = 1.4, title = "BS_Ve", table.legend = FALSE)

dev.off()

#to sort the circ files to get top upregulated go terms:

circ_BSNa_ordered = subset(circ_BSNa, circ_BSNa$adj_pval < 0.05)

circ_OTNa_ordered = subset(circ_OTNa, circ_OTNa$adj_pval < 0.05)

circ_DINa_ordered = subset(circ_DINa, circ_DINa$adj_pval < 0.05)

circ_CENa_ordered = subset(circ_CENa, circ_CENa$adj_pval < 0.05)

circ_TENa_ordered = subset(circ_TENa, circ_TENa$adj_pval < 0.05)

circ_OBNa_ordered = subset(circ_OBNa, circ_OBNa$adj_pval < 0.05)

circ_BSVe_ordered = subset(circ_BSVe, circ_BSVe$adj_pval < 0.05)

circ_OTVe_ordered = subset(circ_OTVe, circ_OTVe$adj_pval < 0.05)

circ_DIVe_ordered = subset(circ_DIVe, circ_DIVe$adj_pval < 0.05)

circ_CEVe_ordered = subset(circ_CEVe, circ_CEVe$adj_pval < 0.05)

circ_TEVe_ordered = subset(circ_TEVe, circ_TEVe$adj_pval < 0.05)

circ_OBVe_ordered = subset(circ_OBVe, circ_OBVe$adj_pval < 0.05)

```

#Summary table CAMERA

```{r}

summary_camera_nasuta = data.frame(geneSetList_GO_nasuta_unique$name_1006, geneSetList_GO_nasuta_unique$go_id, resCAMERA_BSNa$GO_ID, resCAMERA_BSNa$FDR, resCAMERA_TENa$FDR, resCAMERA_OTNa$FDR, resCAMERA_OBNa$FDR, resCAMERA_DINa$FDR, resCAMERA_CENa$FDR)

colnames(summary_camera_nasuta) = c("Term", "go_ID", "GO_ID", "BSNa", "TENa", "OTNa", "OBNa", "DINa"," CENa")

write.table (summary_nasuta_camera, "Summary_CAMERA_Nasuta.txt", col.names = TRUE,row.names = FALSE, quote = FALSE, sep = ";")

summary_camera_ventralis = data.frame(geneSetList_GO_ventralis_unique$name_1006, geneSetList_GO_ventralis_unique$go_id, resCAMERA_BSVe$GO_ID, resCAMERA_BSVe$FDR, resCAMERA_TEVe$FDR, resCAMERA_OTVe$FDR, resCAMERA_OBVe$FDR, resCAMERA_DIVe$FDR, resCAMERA_CEVe$FDR)

colnames(summary_camera_ventralis) = c("Term", "go_ID", "GO_ID", "BSVe", "TEVe", "OTVe", "OBVe", "DIVe"," CEVe")

write.table (summary_camera_ventralis, "Summary_CAMERA_Ventralis.txt", col.names = TRUE,row.names = FALSE, quote = FALSE, sep = ";")

summary_camera = merge(summary_camera_nasuta, summary_camera_ventralis, by.x = "GO_ID", by.y = "GO_ID", all.x = TRUE, all.y = TRUE)

write.table (summary_camera, "Summary_CAMERA.txt", col.names = TRUE,row.names = FALSE, quote = FALSE, sep = ";")

summary_camera_sign = subset(summary_camera, summary_camera$BSNa < 0.05 | summary_camera$TENa < 0.05 | summary_camera$OTNa < 0.05 | summary_camera$OBNa < 0.05 | summary_camera$DINa < 0.05 | summary_camera$CENa < 0.05 | summary_camera$BSVe < 0.05 | summary_camera$TEVe < 0.05 | summary_camera$OTVe < 0.05 | summary_camera$OBVe < 0.05 | summary_camera$DIVe < 0.05 | summary_camera$CEVe < 0.05, na.rm = TRUE)

```
